# Innovative Insights into In Vitro Activity of Colloidal Platinum Nanoparticles against ESBL-Producing Strains of *Escherichia coli* and *Klebsiella pneumoniae*

**DOI:** 10.3390/pharmaceutics14081714

**Published:** 2022-08-17

**Authors:** Damir Vukoja, Josipa Vlainić, Vanja Ljolić Bilić, Lela Martinaga, Iva Rezić, Diana Brlek Gorski, Ivan Kosalec

**Affiliations:** 1 Internal Medicine Clinic, University Hospital Dubrava, 10000 Zagreb, Croatia; 2 Institute for Microbiology, Faculty of Pharmacy and Biochemistry, University of Zagreb, 10000 Zagreb, Croatia; 3 Division of Molecular Medicine, Ruđer Bošković Institute, 10000 Zagreb, Croatia; 4 Department of Applied Chemistry, Faculty of Textile Technology, University of Zagreb, 10000 Zagreb, Croatia; 5 Croatian Institute of Public Health, Rockefeller Str. 7, 10000 Zagreb, Croatia

**Keywords:** platinum nanoparticles, antimicrobial nanoparticles, antibacterial agents, nanomedicine, nanopharmaceuticals, bacterial resistance, multidrug-resistant bacteria

## Abstract

Growing morbidity and mortality rates due to increase in the number of infections caused by MDR (multi-drug resistant) microorganisms are becoming some of the foremost global health issues. Thus, the need to search for and find novel approaches to fight AMR (antimicrobial resistance) has become obligatory. This study aimed to determine the antimicrobial properties of commercially purchased colloidal platinum nanoparticles by examining the existence and potency of their antibacterial effects and investigating the mechanisms by means of which they express these activities. Antimicrobial properties were investigated with respect to standard laboratory ATCC (American Type Cell Culture) and clinical *extended-spectrum beta-lactamase* (ESBL)-producing strains of *Escherichia* (*E*.) *coli* and *Klebsiella* (*K*.) *pneumoniae*. Standard microbiological methods of serial microdilution, modulation of microbial cell death kinetics (“time–kill” assays), and biofilm inhibition were used. Bacterial cell wall damage and ROS (reactive oxygen species) levels were assessed in order to explore the mechanisms of platinum nanoparticles’ antibacterial activities. Platinum nanoparticles showed strong antibacterial effects against all tested bacterial strains, though their antibacterial effects were found to succumb to time kinetics. Antibiofilm activity was modest overall and significantly effective only against *E. coli* strains. By measuring extracellular DNA/RNA and protein concentrations, induced bacterial cell wall damage could be assumed. The determination of ROS levels induced by platinum nanoparticles revealed their possible implication in antibacterial activity. We conclude that platinum nanoparticles exhibit potent antibacterial effects against standard laboratory and resistant strains of *E. coli* and *K. pneumoniae*. Both, cell wall damage and ROS induction could have important role as mechanisms of antibacterial activity, and, require further investigation.

## 1. Introduction

Antimicrobial resistance has emerged as one of the leading global health threats and public health concerns. Although a natural process, the rate at which pathogenic bacteria are becoming resistant to existing antibiotics and the occurrence of multidrug-resistant (MDR) strains has increased. Achieving control over infections is challenging and is connected to loss of health, lives, and resources. Therefore, it is unquestionable that new solutions, approaches, and therapeutic agents are needed [1,2,3,4,5]. Among the extensively researched alternatives to conventional antibiotics and other antimicrobial agents, considerable attention has been given to nanoparticles [2,3,4,5,6,7,8,9,10,11]. 

The antimicrobial effects of nanoparticles have been proven for several metal and metal oxide particles, which has prompted research into their use as antimicrobial agents, even in the context of action on intracellular bacteria and biofilms [2,3,6,7,8,12,13,14,15,16,17,18]. The antimicrobial properties of nanoparticles can be primarily attributed to their large surface-to-volume ratios, which allow them to achieve close interactions with microbial membranes [4,8,19]. Favorable properties of nanoparticles, such as the low concentrations at which they achieve their effects, their biocompatibility, synergism with antibiotics, and low tendency to induce resistance, make them ideal candidates for next-generation antimicrobial drugs [4,11,13,20].

Recently, there has been growing interest in platinum nanoparticles (nanoplatinum, nPt) [21,22,23,24], which are considered as antioxidants and appear to possess catalytic activities utilized in wound treatment and regenerative medicine [25,26]. Evidence suggests that nPt can damage DNA, which leads to their possible application in the treatment of malignancies [21,27,28]. However, their cytotoxicity, which is still a subject of debate [29,30,31], does not necessarily exclude the possibility of their use as antimicrobial agents. Several studies have shown a reduction in the cytotoxicity of platinum nanoparticles “coated” with biocompatible materials, with physicochemical modifications and/or surface functionalization. Additionally, there are options for their application to medical surfaces and materials, as well as in extracorporeal systems [25,29,32,33].

Evidence suggests an antibacterial and antifungal effect of nPt, as well as an inhibitory effect on biofilms, which depends on particle size and surface characteristics [19,29,30]. Proposed mechanisms of such action are increased production of intracellular ATP, which inhibits bacterial growth and leads to DNA damage [29], along with cell wall damage [34,35]. Moreover, the synergistic antibacterial effect of nPt with antibiotics and other nanoparticles has also been reported [19,29,35,36,37].

As compelling evidence suggests, Gram-negative bacteria from the family of enterobacteria (Enterobacteriaceae), *Escherichia (E.) coli* and *Klebsiella (K.) pneumoniae* are among the most common causes of infections in humans. This study’s particular emphasis is on *extended-spectrum beta-lactamase* (ESBL)-producing strains of these bacteria, as they have become a growing problem of epidemic proportions [38,39,40].

In this study, investigation of the antibacterial properties of colloidal platinum nanoparticles towards both clinically multidrug-resistant *E. coli* and *K. pneumoniae* has been performed for the first time. Thus, it provides broader insights into the antibacterial properties of nPt, with an emphasis on the potential mechanisms of such action. In light of this, the paper intends to advance current research on platinum nanoparticles as a potential antibacterial agent even against MDR bacteria. 

## 2. Materials and Methods

### 2.1. Bacterial Species, Growing Conditions, and Inoculum Preparation

In order to test the antibacterial effect of platinum nanoparticles, the following bacterial species were used in this study: *Escherichia coli* strain ATCC 10536 (according to the *American Type Culture Collection*) and *E. coli* ESBL+ MFBF 12795 (*extended-spectrum beta-lactamase*); *Klebsiella pneumoniae* strain ATCC 700603 and *K. pneumoniae* ESBL+ MFBF 10690. Bacterial species were obtained from the Collection of Microbial Cultures of the Institute for Microbiology, Faculty of Pharmacy and Biochemistry, University of Zagreb (MFBF).

Fresh bacterial cultures, previously grown on trypticase–soy agar (TSA), were incubated for 18–24 h at 37 °C under aerobic conditions in the dark (Sanyo MIR-533, Osaka, Japan). Inoculums were prepared by suspending bacterial colonies in appropriate solution (saline, 3% *w/v* glucose solution, M9 minimal medium, or their combination). The choice of solution was conditioned by the type of experiment performed. Bacterial suspensions were 1.5 × 10^8^ CFU/mL, 3.0 × 10^8^ CFU/mL, and 1.0 × 10^9^ CFU/mL, respectively [41].

### 2.2. Susceptibility Testing of ESBL-Producing Strains

Antimicrobial susceptibility tests were performed with VITEK^®^ 2 (BioMerieux, Marcy-l’Étoile, France), an automated instrument using a turbidimetric method. VITEK^®^ cards for susceptibility testing (AST) were inoculated with 0.5–0.63 McFarland units and incubated according to the manufacturer’s instructions (Vitek^®^ 2 AST-N379, BioMerieux, Marcy-l’Étoile, France). The instrument performs its susceptibility analyses by monitoring growth and activity. We analyzed the inhibitory effects of cefepime, cefotaxime, and ceftazidime with and without clavulanic acid (CA) for ESBL strains. If the MIC concentration for cephalosporin decreased with clavulanic acid compared to cephalosporin alone, this was considered as evidence of ESBL production [42].

### 2.3. Determination of MIC and MBC Values—Serial Microdilution Method

Minimum inhibitory (MIC) and minimum bactericidal (MBC) concentrations were determined using a modified double serial microdilution method according to EUCAST guidelines [43]. Instead of standard Müller–Hinton broth, saline was used as medium. The reason for the modification is the tendency of nanoparticles to show significantly weaker antibacterial activity in media containing components with which they can interact, such as broth proteins or serum components [4,44,45]. 

Dilutions of the tested colloid of platinum nanoparticles were prepared by adding 100 µL of saline to the wells of 96-well polystyrene microtiter plates (DeltaLab, Barcelona, Spain) and then adding 100 µL of a nPt colloid solution to the first well. In order to ensure the reproducibility of the methods, a commercially purchased platinum nanoparticle dispersion in water (colloid) was used (3 nm particle size, 1000 ppm; Sigma Aldrich, St. Louis, MO, USA). The nPt colloid from the original manufacturer’s stock was previously diluted in saline to a concentration of 200 ppm. A mixture of saline and inoculum was used as a positive control, while pure saline was used as a negative control. Incubation was performed for 18–24 h at 37 °C under aerobic conditions in the dark (Sanyo MIR-533, Osaka, Japan). After incubation of the microtiter plates, 10 μL of each dilution was applied by a sterile microbiological needle to the TSA sectors and incubated. MBC was determined as the lowest concentration at which there was no visible bacterial growth or 99.9% of bacteria was killed on a solid nutrient medium, while MIC was determined as the lowest concentration at which the growth of bacteria on a solid nutrient medium was reduced by 80% compared to the control [46,47].

### 2.4. Testing the Antibacterial Effect over Time—“Time–Kill” Method

Monitoring of the antimicrobial effect of one or more antimicrobial substances as a function of time (kinetics of death) was carried out using the “time–kill” method [46,47]. Saline was used as medium [45,48]. A mixture containing an inoculum (1.5 × 10^7^ CFU/mL) and nPt at a concentration twice the MIC was prepared. Inoculum was used as a control. Samples were stirred continuously for 24 h on an orbital shaker–incubator (Orbital Shaker–Incubator ES-20, Grant bio, Cambridge, UK) at a speed of 200 RPM and a temperature of 37 °C. The antibacterial effect of nPt was monitored at times t0 (immediately after preparation of the mixture), t1 (after 1 h), t3 (after 3 h), t6 (after 6 h), t18 (after 18 h), and t24 (after 24 h), while the control was monitored at times t0, t6, and t24. Sampling was performed in duplicate. After taking 100 µL, serial ten-fold dilutions of the test and control samples were performed. From each dilution obtained, 50 µL was taken and applied to the TSA’s surface, smeared evenly with a sterile L-stick, and then incubated for 18–24 h at 37 °C under aerobic conditions in the dark (Sanyo MIR-533, Osaka, Japan). The number of colonies was calculated and expressed as the logarithm of the number of CFUs (colony forming units) per milliliter:

log_10_ [number of colonies (CFU/mL)] = log_10_ (number of counted colonies × 10 number of dilutions + 1).

### 2.5. Biofilm Inhibition

To assess the activity of nPt against *E. coli* and *K. pneumoniae* biofilms, an inhibition of biofilm formation assay and an analysis of antibiofilm effect on preformed (mature) biofilms were performed. The M9 minimal medium with 3% glucose was chosen, since it does not contain proteins that could interfere with nanoparticle activity [45,49,50] and is nutritive to support bacterial growth [41].

#### 2.5.1. Biofilm Formation Assay

Inoculum (1.0 × 10^9^ CFU/mL) and appropriate dilutions were seeded and incubated for 72 h at 37 °C. After incubation, supernatants were collected from the wells and seeded to agar sectors in order to assess the viability of bacteria. In addition, the well contents were removed, and the plate was washed 3 times with sterile distilled water. The plate was then air-dried, and methanol (50 µL, 15 min) was added to fix the biofilm. This step was followed by drying and the addition of Crystal Violet stain (0.5% in 10% methanol; Clin-tech, Guildford, UK) for 15 min. The dye was then removed, and the sample plate was washed again 3 times with sterile distilled water and allowed to air-dry at room temperature. Finally, ethanol (100 µL) was added, and the plate was left to stir continuously for 10 min at medium speed to achieve biofilm dissolution. Optical density was measured photometrically at 570 nm (Multiskan EX, Thermo Fisher Scientific, Vantaa, Finland).

#### 2.5.2. Antibiofilm Analysis on Preformed (Mature) Biofilms

This experiment was carried out according to the previously described procedure, except that the nPt was added to plates preincubated for 48 h (biofilm was formed prior to nPt addition). An additional 24 h incubation followed. The experiments were carried out in triplicate.

### 2.6. Viability of Bacteria 48–72 h after Nanoparticle Exposure

With some modifications, the experiment was carried out as described in the available relevant literature [51,52]. In brief, after 48 h of incubation (samples prepared for antibiofilm activity assessment), the supernatants were carefully collected by laying down microbiological eyelets and then applied to agar plates. The plates were incubated overnight at 37 °C to allow the bacterial colonies to grow. The same procedure was repeated after another 24 h of incubation of the same samples and after a total of 72 h of incubation of the bacteria. After the incubation, the growth of bacterial colonies on particular agar plate sectors was evaluated and documented.

### 2.7. ROS Formation under the Action of Platinum Nanoparticles

Triplets for each bacterial strain tested were placed on a 96-well black microtiter plate for cell culture by adding an equal volume of colloidal platinum nanoparticle solution at the appropriate 2 × MIC, 1 × MIC, and ½ × MIC concentrations to the inoculum (0.5 McFarland units). The plate was incubated at 37 °C for 1, 3, 6, 12, and 24 h. After incubation, a peroxynitrite indicator, 2′-7′-dichlorodihydrofluorescein diacetate (DCFH-DA) (Sigma-Aldrich, Gillingham, UK) (final concentration: 5 µM) was added to the wells. In order to achieve better bonding of the dye to ROS, the light-protected plate was placed on an electric mixer for microtiter plates at medium speed (348/1 “ASSISTENT” Rocking shaker, CE type, Karl Hecht GmbH & Co., Sondheim vor der Rhön, Germany). Fluorescence intensity was measured at 485 nm excitation and 525 nm emission (Infinite 200 microplate reader, Tecan Group Ltd., Männedorf, Switzerland) [53,54].

### 2.8. Determination of Bacterial Cell Wall Permeability by the Action of Platinum Nanoparticles—DNA/RNA and Protein Leakage

To determine the effect of platinum nanoparticles on the bacterial wall, a standard method for testing cell membrane integrity was used [35,54]. To gain insights into the dynamics and time course, the measurements were performed after 0, 1, 3, 6, 18, and 24 h of incubation. Inoculum density was 0.5 McFarland units, and the platinum nanoparticle concentration was 2 × MIC, MIC, and ½ × MIC. As a negative control, inoculated M9 medium with 3% glucose was used, and for the positive control (a model of massive cell wall damage) inoculum samples were treated with ethanol. After appropriate incubation periods, the samples were centrifuged (1250 rpm, 2 min), followed by measurements selective for protein or DNA/RNA (Biospec Nano, Shimadzu, Torrance, CA, USA).

### 2.9. Nanoparticle Tracking Analysis of Original Colloidal Platinum Nanoparticle Samples

Colloidal stabilized samples were taken from the original stock of colloidal platinum nanoparticles for which the manufacturer had stated a nanoparticle size of 3 nm. The samples were homogenized in a vortex apparatus for 25–30 s before measurement. The instrument (Nanosight LM10, MalvernPanalytical, Malvern, UK) was rinsed to wash away any impurities or residues. Samples were taken with a sterile syringe, then ‘injected’, and the analysis was performed. All analyses were performed in triplicate, and mean values were determined from the obtained data [51]. 

### 2.10. Statistical Data Processing

Data were collected in an MS Excel database (version 11. Microsoft Corporation, Redmond, WA, USA), while the GraphPad Prism 9 software package (GraphPad Software, Inc., San Diego, CA, USA) and XLSTAT 2022 (MS Excel add-in software, Addinsoft, Paris, France) were used for statistical analysis. All values are expressed as means ± standard deviations of *n* independent measurements. Most data were processed using a variance analysis test (ANOVA) or the Student’s *t*-test, whilst the Friedman test was used for ROS detection and cell leakage. Statistical significance was considered with *p* < 0.05. 

## 3. Results

### 3.1. Susceptibility of Clinical ESBL-Positive Strains and the MIC and MBC Achieved by Colloidal Platinum Nanoparticles

The results of an antimicrobial susceptibility analysis are shown in Table 1. Susceptibility of these ESBL-producing strains to various antimicrobials is expressed as MIC values and characterized as: susceptible (S), intermediate (I), and resistant (R), respectively. 

MIC (minimum inhibitory concentration) and MBC (minimum bactericidal concentration) of colloidal nPt were determined in two media, saline and M9 minimal salts with 3% glucose. As explained in the Materials and Methods section, these media were chosen because they do not contain protein components which could interact with nanoparticles and diminish their activity. 

MIC and MBC values of nPt for *E. coli* ATCC 10536, *E. coli* ESBL+ MFBF 12795, *K. pneumoniae* ATCC 700603, and *K. pneumoniae* ESBL+ MFBF 10690 in saline are shown in Table 2. The values are expressed as concentrations (ppm = parts per million, equal to µg/mL) with corresponding mean values and standard deviations (SDs). As shown in Table 2, nPt has an antibacterial effect against all the tested bacterial strains in vitro.

The lowest MBC value was found for *E. coli* ATCC 10536, while the highest was found for *K. pneumoniae* ATCC 700603. This difference is also statistically significant (as determined by the Kruskal–Wallis test, *p* = 0.001). The lowest MIC value obtained for platinum nanoparticles was against *K. pneumoniae* ESBL+ MFBF 10690, while the highest values were obtained against *E. coli* ESBL+ MFBF 12795 and *K. pneumoniae* ATCC 700603. The data analysis did not reveal a statistically significant difference (Kruskal–Wallis test, *p* < 0.05) between these values.

When bacteria were grown in M9 minimal salts with 3% glucose as a more nutritious medium, MIC values between ~1.75 and ~5 times higher (mean: 2.88× ± 1.45) were observed (Table 3). On the contrary, the MBC values obtained in this enriched medium were between ~0.25 times lower and ~2.25 times higher (mean: 0.55× ± 1.15) than in the saline medium (Table 3).

### 3.2. Antibacterial Effect of Colloidal Solution of Platinum Nanoparticles over Time—“Time–Kill” Kinetics Assay

**Figure 1:**
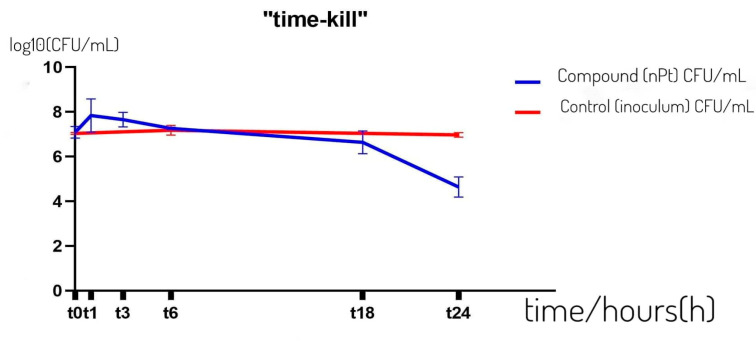
Number of colonies of *Klebsiella pneumoniae* ESBL+ MFBF 10690 over time (h) treated with nPt (c = 2 × MIC) relative to the control. CFU—colony forming unit; t—time.

Figure 1 shows the antibacterial effect of 2 × MIC of platinum nanoparticles on the resistant clinical strain of *K. pneumoniae* ESBL+ MFBF 10690 over time in a saline medium, tested according to the *time–kill* method. The graph shows that no bactericidal effect was achieved within 24 h, but a decrease in the number of bacteria in the sample with nPt was observed in comparison to the control following six hours of incubation.

A statistically significant decrease in the number of colonies of *K. pneumoniae* ESBL+ was observed in the time interval t18–t24 (as determined by ANOVA followed by the Mann–Whitney U test, *p* < 0.05). The decrease in the number of colonies in the sample after 24 h relative to the control was also statistically significantly lower (ANOVA followed by the Mann–Whitney U test, *p* < 0.05). 

### 3.3. Inhibition of Biofilm by Platinum Nanoparticles

**Figure 2:**
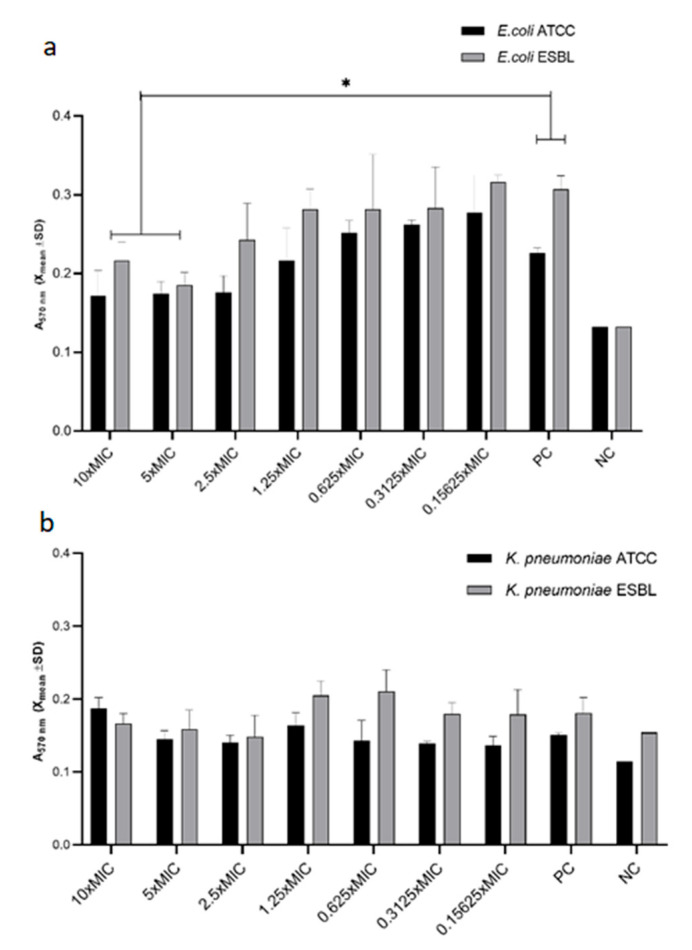
Effect of various concentrations of platinum nanoparticles on biofilm formation of (**a**) *E. coli* ATCC and *E. coli* ESBL and (**b**) *K. pneumoniae* ATCC and *K. pneumoniae* ESBL compared to the control. A_570nm_—absorbance at 570 nm wavelength; PC—positive control (untreated bacteria); NC—negative control (sterile medium). * statistically significant difference (*p* < 0.05)

As shown in Figure 2, nPt inhibited biofilm formation by both the ATCC and ESBL-producing strains of *E. coli* in a concentration-dependent manner. This effect was statistically significant for 10 × MIC and 5 × MIC of nPt for both strains (*p* < 0.05). Significant inhibition was achieved with 10 × MIC and 5 × MIC of nPt, which correspond to 218.7 ppm and 109.35 ppm of platinum nanoparticles, respectively. This is equal to 28.48% ± 2.3% and 38.73% ± 1.6% of *E. coli* ESBL biofilm inhibition compared to the control (*p* < 0.05), respectively.

Although it seems that there was no significant inhibition of biofilm formation for the *K. pneumoniae* strains (Figure 2), these results should be interpreted with caution, since the results for the positive control were not significantly different from those for the negative control with respect to the grown biofilm. It can be assumed that the biofilm growth of the *K. pneumoniae* strains could be different in some other medium and give different results or that some nutrients are not present which would allow extensive extracellular matrix formation in these bacteria.

**Figure 3:**
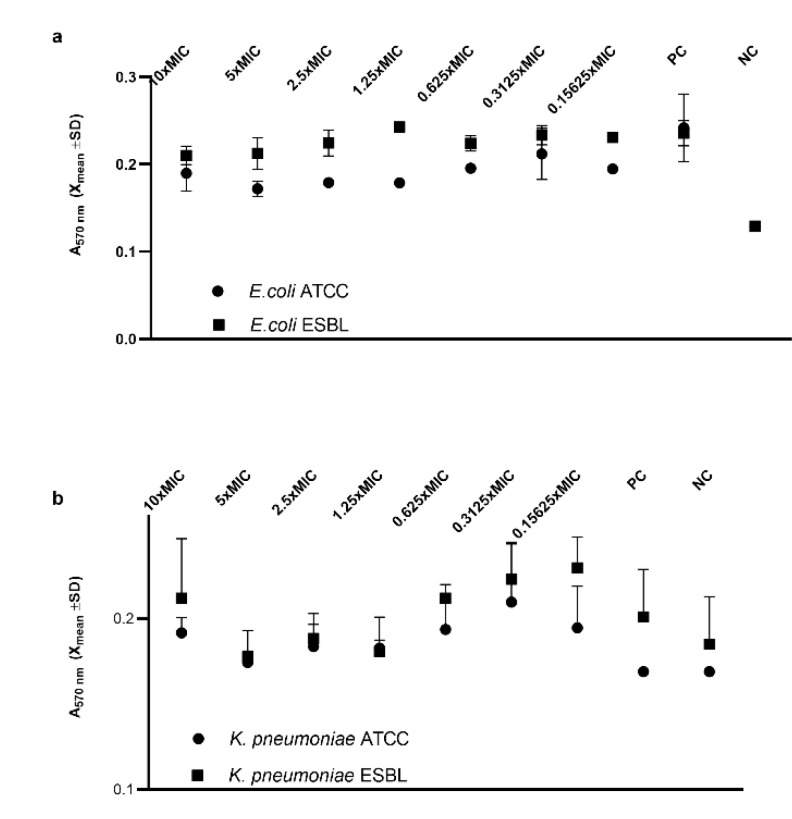
(**a**) Effects of various concentrations of platinum nanoparticles on preformed (mature) biofilms of *E. coli* ATCC and *E. coli* ESBL compared to the control. (**b**) Effects of various concentrations of platinum nanoparticles on preformed (mature) biofilms of *K. pneumoniae* ATCC and *K. pneumoniae* ESBL compared to the control. A_570nm_—absorbance at 570 nm wavelength; PC—positive control (untreated bacteria); NC—negative control (sterile medium).

Inhibition of already formed biofilm was not achieved using nPt in our conditions (Figure 3), although it seems that higher concentrations of nPt elicited some inhibition of *E. coli* ATCC biofilm (not statistically significant) (Figure 3a). Similarly, it seems that biofilms of *K. pneumoniae* strains were not completely formed compared to the negative control (Figure 3b).

### 3.4. Viability of Bacteria 48–72 h after Nanoparticle Exposure

**Figure 4:**
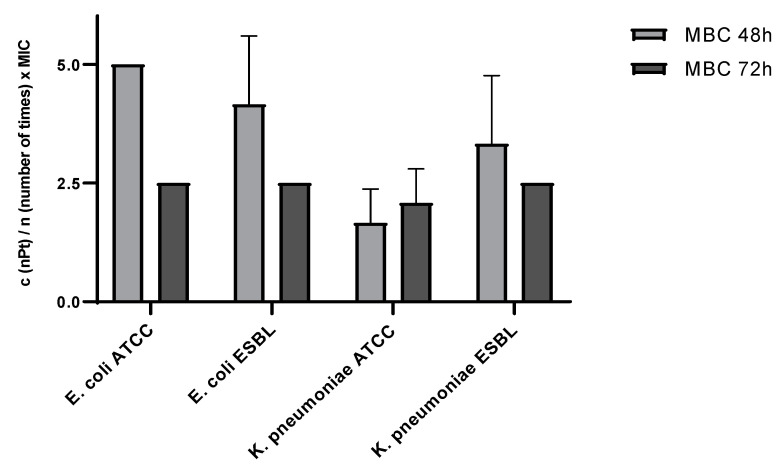
Comparison of bacterial planktonic cell survivability after 48 and 72 h of platinum nanoparticle exposure. Values are expressed as means ± standard deviations. c—concentration.

As shown in Figure 4, no bacterial viability was observed at approximately 2 × MIC for *K. pneumoniae* ATCC (2.08 × MIC ± 0.72 SD) and at 2.5 × MIC for *E. coli* ATCC, *E. coli* ESBL, and *K. pneumoniae* ESBL.

When analyzing the survival dynamics of the planktonic bacterial cells between 48 and 72 h of nPt exposure, one can observe that, for most tested species, the survival decreased from 48 to 72 h of exposure (Figure 4). The decrease in the viability of bacterial cells between these two times was statistically significant for *E. coli* ATCC (multiple *t*-test, *p* < 0.05), although the overall survival of *K. pneumoniae* ATCC planktonic cells exposed to nPt was lowest compared to the other tested bacterial species (multiple *t*-test, *p* < 0.05).

### 3.5. Effect of Platinum Nanoparticles on ROS Release

Statistical analysis (Supplementary Figures S1–S29 and Tables S1–S28) of the results (Figure 5) did not reveal significant differences between the samples at each time point, except in the case of *E. coli* ESBL+ MFBF 12795 at the zero time point, for which the average ROS levels of the samples treated with 2 × MIC of nPt were statistically higher (*p* = 0.039) than the negative control samples (untreated bacteria/inoculum). However, overall ROS levels (sum of values from all time points) for the samples of bacteria treated with 2 × MIC of nPt of all tested bacterial strains were statistically higher (*p* < 0.05) than the values obtained for inoculums of each tested bacterial strain (untreated bacteria, i.e., the negative control). The results show that the highest increase in ROS levels among the tested strains was induced by 2 × MIC of nPt for *E. coli* ESBL+ MFBF 12795 [+39.92%, *p* < 0.0001)].

### 3.6. Effect of Platinum Nanoparticles on Cell Wall Permeability

**Figure 6:**
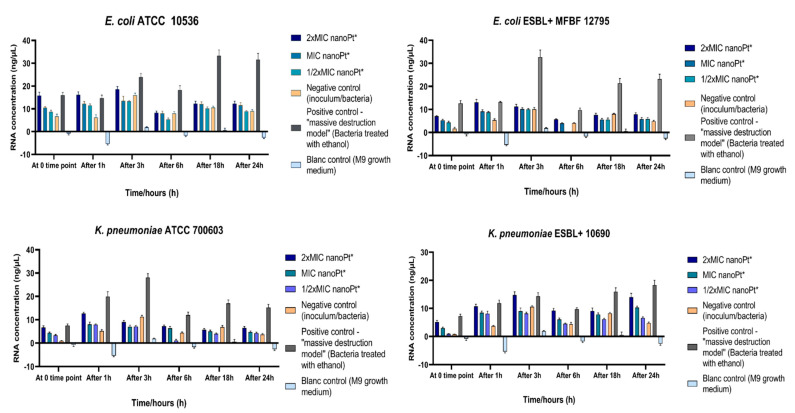
Interrelations of extracellular DNA/RNA concentrations over time for all tested bacterial strains. nanoPt*—platinum nanoparticles.

**Figure 7:**
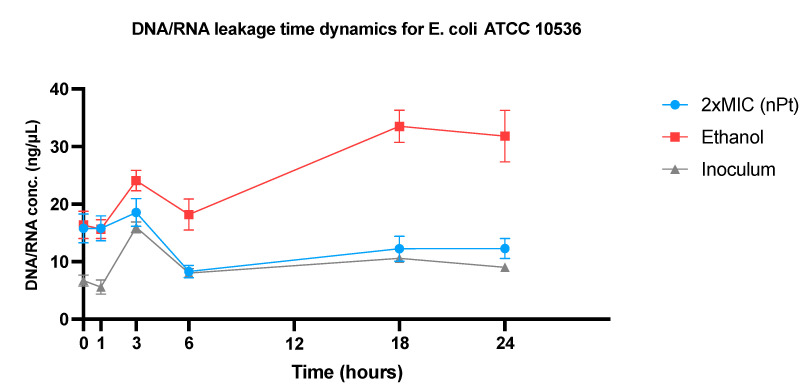
Graphical representation of DNA/RNA leakage (release) dynamics for *E. coli* ATCC 10536 at several time points of measurement over a 24 h period. The average values measured for each time point for samples of bacteria treated with 2 × MIC of nPt, bacteria treated with ethanol (as positive control), and untreated bacteria (inoculum, i.e., the negative control) are shown (along with standard deviations) and compared.

**Figure 8:**
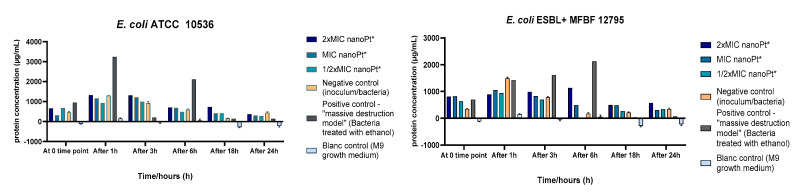

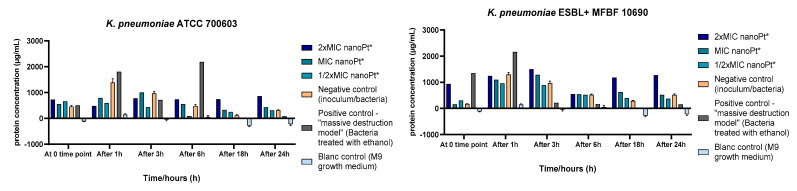
Interrelation of extracellular protein concentrations over time for all tested bacterial strains. nanoPt*—platinum nanoparticles.

The results are shown graphically for all tested bacterial strains and are presented as the amounts of DNA/RNA (Figure 6), with a single bacterial strain DNA/RNA release time dynamics representation (Figure 7), and protein released from intracellular compartments (Figure 8).

The results showed changes in extracellular DNA/RNA concentrations between the time points of measurements for each bacterial strain, mostly in terms of apparent increase from time zero to 1 h and further after 3 h of incubation. At the third hour, an increase in extracellular DNA/RNA in untreated bacteria was observed, with enhancement after 18 and 24 h of incubation (which did not reach statistical significance with *p* < 0.05), except for *K. pneumoniae* ATCC 700603 (Figure 6 and Figure 7). However, when comparing the total DNA/RNA concentrations (as a sum of the measurements for all time points), there were statistically significant differences (Supplementary Figures S30–S58 and Tables S29–S56): the total DNA/RNA concentration obtained for *E. coli* ATCC 10536 treated with 2 × MIC of nPt was statistically higher compared to bacteria treated with 1/2 × MIC of nPt (*p* = 0.014), although the result was not statistically significant compared to untreated bacteria (*p* = 0.067). For *E. coli* ESBL+ MFBF 12795, the total DNA/RNA concentration for the sample of bacteria treated with 2 × MIC of nPt was significantly higher than that for the sample of untreated bacteria (*p* = 0.041, Supplementary Figure S43 and Table S42). In contrast, for *K. pneumoniae* ATCC 700603, the total DNA/RNA concentration of samples treated with nPt was not significantly higher than that of the untreated bacteria. Lastly, for *K. pneumoniae* ESBL+ MFBF 10690, the total DNA/RNA concentration obtained for the samples of bacteria treated with 2 × MIC of nPt was statistically higher than that obtained for the samples treated with 1/2 × MIC of nPt (*p* = 0.001) and untreated samples as well (*p* = 0.006).

A pattern of protein leakage time dynamics similar to that for DNA/RNA leakage was observed (Figure 8). Statistical analysis of the results did not reveal a significant difference in extracellular protein concentrations between the different time points (Supplementary Figures S59–S87 and Tables S57–S84). However, when the total extracellular protein concentrations (as a sum of measurements of all time points) were compared, there were statistically significant differences for ESBL+ strains: for *E. coli* ESBL+ MFBF 12795 treated with 2 × MIC, the total extracellular protein concentration was significantly higher compared to the bacteria treated with 1/2 × MIC of nPt (*p* = 0.041). Further, for *K. pneumoniae* ESBL+ MFBF, a statistically higher protein leakage was noted for samples treated with 2 × MIC than for samples treated with 1/2 × MIC (*p* = 0.008), but this was also statistically higher compared to samples of bacteria treated with ethanol (*p* = 0.025, Supplementary Figure S86 and Table S84).

With regard to the overall response of each tested bacterial strain to platinum nanoparticles in terms of protein leakage, the highest increase (43.47%) induced by 2 × MIC of nPt was found for *K. pneumoniae* ESBL+ MFBF 10690. The extracellular protein concentration for *K. pneumoniae* ESBL+ MFBF 10690 treated with 2 × MIC of nPt was statistically higher than those measured for *K. pneumoniae* ATCC 700603 and *E. coli* ESBL+ MFBF 12795 (*p* < 0.05, Supplementary Figure S87). For both clinically resistant strains of *E. coli* and *K. pneumoniae* (ESBL+), the percentage increase in total extracellular protein levels for the samples treated with 2 × MIC of nPt was higher than those recorded for the standard laboratory strains of the same bacterial species.

### 3.7. Nanoparticle Tracking Analysis of the Original Colloidal Platinum Nanoparticle Samples

**Figure 9:**
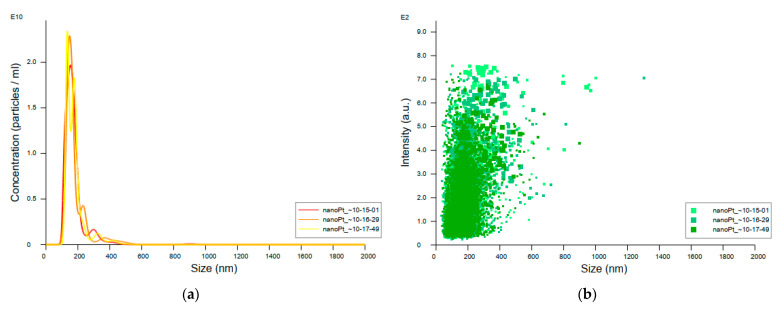
Nanoparticle Tracking Analyzer data obtained after analysis of original colloidal platinum nanoparticle samples: (**a**) distribution of particle sizes by concentration; (**b**) distribution of particle sizes by intensity (*n* = 3).

Data on the concentration, distribution, and size (mean and mode) of nanoparticles in the sample prepared from the original colloidal platinum nanoparticles stock revealed the existence of larger particle sizes than expected considering the size of 3 nm stated by the manufacturer. As can be seen, the peaks of measured particle size fall between 100 and 200 nm, with a mean value of 177.5 nm ± 71.2 nm (Figure 9).

## 4. Discussion

The developing field of nano(bio)medicine is making an immense contribution to human health and wellbeing. Most significant is the proven antimicrobial activity of numerous nanomaterials against MDR pathogens which have emerged as promising alternative treatment options. Potent antimicrobial effects, alongside other favorable properties, have been reported for metallic nanoparticles [4,12,56,57]. Although there are many reports regarding the investigation of the antibacterial properties of metallic nanoparticles and application of them, only a minority have included resistant bacterial species [7,58]. 

The results clearly show that nPt has antibacterial effects against *E. coli* and *K. pneumoniae*. Using the MBC/MIC ratio, the bactericidal and/or bacteriostatic activity of nPt towards these strains can be determined. In particular, this ratio equal or less than 4 (MBC/MIC ≤ 4) is considered as milestone of bactericidal effect, while values greater than this are considered as evidence of bacteriostatic effect [55]. According to this criterion, platinum nanoparticles are bactericidal for all examined strains except for *K. pneumoniae* ESBL+ MFBF 10690, against which they act bacteriostatically. Higher MIC values, as well as MBC values, achieved in M9 minimal medium as a richer type of growth medium are expected and rational. It is known that differences in growth medium composition can affect the activity, as more complex growth media require higher concentration of a substance to achieve antimicrobial effects [44,59]. The obtained results are in accordance with those of other recent reports in the literature, suggesting that these nanoparticles could be considered potent antibacterial agents [60,61,62]. 

The effectiveness of nPt was reaffirmed in the “*time–kill*” kinetics assay. Although after 24 h of incubation, no bactericidal effect was observed, the decrease in the number of colonies compared to the control was significant, probably due to the bacteriostatic effect of nPt for ESBL-producing strains of *K. pneumoniae*. An additional factor to consider is the concentration of nPt used in this assay, which is twice the MIC concentration. The double MIC value determined for *K. pneumoniae* ESBL+ is not higher than its MBC value, whereas bacteriostatic effect (after 24 h of exposure) is evident. It seems that significant inhibition of bacterial cells starts after at least 18 h of exposure, indicating a time-dependent effect as documented by other authors [30,45].

Significant inhibition of biofilm formation for both ATCC and ESBL-producing *E. coli* strains was observed in a concentration-dependent manner. On the contrary, since biofilm formation was weak even in the control for the *K. pneumoniae* strains, we cannot take the obtained results unreservedly. Additionally, no significant antibiofilm effect of nPt on the preformed (mature) biofilms of *E. coli* strains was observed, whilst the results for the *K. pneumoniae* strains were not firm enough for interpretation. Although there have been reports of potent antibiofilm effects for some noble nanoparticles, especially silver nanoparticles, data on the antibiofilm activity of platinum nanoparticles are scarce [51,53]. However, the most recent study by Chlumsky et al. reported modest antibiofilm efficacy for nPt, which is consistent with our results [30].

Investigation of the survival of planktonic bacterial cells after 48 and 72 h of exposure to nPt provided almost unambiguous findings. After 72 h of exposure to nPt, no bacterial cell viability was observed with exposure to 2.5 × MIC. Additionally, a trend of reduction in nPt concentration needed to achieve the eradication of viable bacterial cells (MBC) from 48 to 72 h of exposure was evident. These results correlate with the previous finding from the time–kill assay, thus suggesting the time-dependent antibacterial effect. 

An ROS-mediated mechanism of antimicrobial action has been previously reported for some metal nanoparticles [63], while for platinum nanoparticles there are just a few ambiguous reports [34,35]. The results showed that all tested bacterial strains responded to 2 × MIC of nPt, with significant enhancement of total ROS levels, whereas MDR strains responded with a higher increase, and *E. coli* responded with an overall greater increase than *K. pneumoniae*. 

Our results suggest significant bacterial cell wall damage, and a similar effect was obtained with ethanol. Cell wall damage caused by nPt occurs in the first 3 h of exposure. Interestingly, the observation contrasts with that for ethanol, which tends to cause an increase in extracellular DNA/RNA concentrations after 6 and up to 24 h of bacterial exposure. Another important finding is that both ESBL-producing strains of *E. coli* and *K. pneumoniae* treated exhibited greater increases in extracellular DNA/RNA levels than standard laboratory strains. This may suggest the greater susceptibility to nPt of MDR strains. On the basis of our results, concentration- and time-dependent effects of platinum nanoparticles on bacterial cell wall permeability and integrity are in agreement with recent literature reports [34,35], whereas bacterial cell wall integrity was also disturbed by various metal nanoparticles [30,34,35,63]. 

Assuming the aggregation of the nanoparticles in the original solution and the possible repercussions of this, measurement of particle size in the original colloidal solution by mass spectrometry was performed and a deviation from the manufacturer’s stated particle size was discovered: the average detected particle size in the original colloidal solution of platinum nanoparticles was 177.5 nm, with a standard deviation of 71.2 nm, while the declared particle size was 3 nm. As a size-dependent antibacterial effect of nanoparticles has been proven [30,48], this may have implications for our study as well. Following these observations, it can be supposed that smaller-sized platinum nanoparticles would show even more potent antibacterial activities than those presented here, especially since the spontaneous tendency to agglomeration may affect results. Keeping in mind that this study involved a biological experiment, we did not use organic stabilizers because there are no available established protocols for their use when applying nPt as an antimicrobial agent and they could have affected the results. Therefore, considering that the research on the antibacterial properties of nPt still insufficient, there is an immense range of possible methodological approaches and improvements that can be implemented. Accordingly, there is enormous potential and an open future for this exciting field of research. 

## 5. Conclusions

Based on the presented data, it can be concluded that platinum nanoparticles exhibit a potent antibacterial activity towards both standard laboratory and clinically resistant ESBL-producing strains of *E. coli* and *K. pneumoniae*. Their antibacterial effects seem to be concentration- and time-dependent. Significant inhibition of biofilm formation in both laboratory and clinically resistant strains of *E. coli* was expressed, with no significant effect against preformed biofilms, suggesting overall modest antibiofilm activity toward these bacterial species. In contrast, an antibiofilm effect for *K. pneumoniae* could not be clearly determined. 

A mechanism mediated by ROS production appears to be implicated in achieving the antibacterial effect of platinum nanoparticles. One of the key mechanisms by which platinum nanoparticles achieve their antibacterial effects could be their ability to cause damage to bacterial walls. A tendency of colloidal platinum nanoparticles to spontaneous agglomeration was also found. 

Considering the presented conclusions, platinum nanoparticles show promise as a potential novel antibacterial agent, especially in the context of response to infections caused by MDR bacteria. Accordingly, there is a need for additional research, not only under in vitro conditions, but in in vivo settings as well. Translational studies should be carried out, too, since without them it will not be possible for many of these important findings to be applied in practice. 

## Figures and Tables

**Figure 5:**
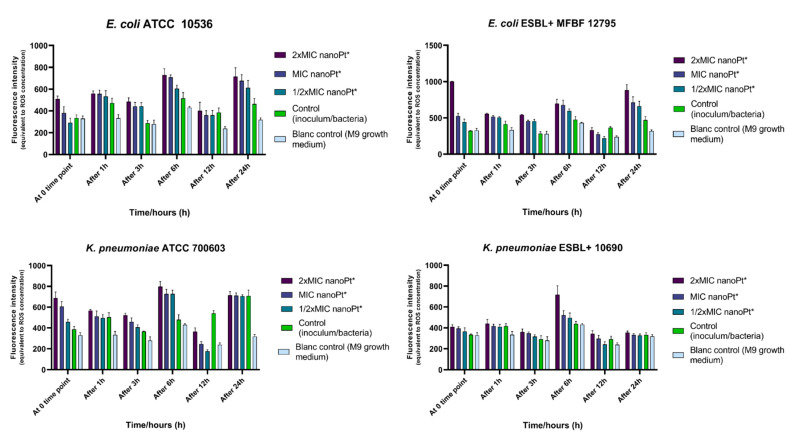
Interrelations of ROS concentrations in the extracellular compartments of all samples and time dependence for all tested bacterial strains. nanoPt*—platinum nanoparticles.

**Table 1:** Susceptibility testing of ESBL-positive clinical strains used in the study.

Bacterial Strain	MIC (µg mL^−1^)													
	Ampicillin	Amoxicillin/ Clavulanic Acid	Piperacillin/ Tazobactam	Cefotaxime	Ceftazidime	Cefepime	Imipenem	Meropenem	Amikacin	Gentamicin	Ciprofloxacin	Norfloxacin	Fosfomycin	Trimethoprim/ Sulfamethoxazole
*E. coli* MFBF 12795 ESBL+	8 (R)	8 (S)	64 (R)	2 (I)	16 (R)	4 (I)	2 (S)	0.5 (S)	4 (S)	≦1 (S)	≦0.25 (S)	1 (I)	≦16 (S)	≦20 (S)
*K. pneumoniae* MFBF 10690 ESBL+	≧32 (R)	16 (R)	8 (R)	≦1 (S)	2 (I)	≦1 (S)	2 (S)	2 (S)	4 (S)	2 (S)	≦0.25 (S)	1 ((I)	≧256 (R)	≦20 (S)

MFBF—clinical isolates from the Collection of Microbes, University of Zagreb, Faculty of Pharmacy and Biochemistry Institute for Microbiology; ESBL—extended-spectrum beta-lactamases; MIC—minimal inhibitory concentration; R—resistant; S—susceptible; I—intermediate.

**Table 2:** MBC and MIC values for colloidal solutions of platinum nanoparticles for the tested bacterial strains of *E. coli* and *K. pneumoniae* in a saline medium. Concentrations are expressed as ppm (i.e., *parts per million*), equal to µg mL^−1^.

Bacterial Strain	MBC/ppm	MIC/ppm	MBC/MIC Ratio	Antibacterial Effect (According to MBC/MIC Ratio) [55]
	$\bar{X}\pm S.D.\;\left(N=3\right)$	$\bar{X}\pm S.D.\;\left(N=3\right)$		
*E. coli* ATCC 10536	8.33 ± 3.61	4.17 ± 1.80	~2	Bactericidal
*E. coli* ESBL+ MFBF 12795	25.00 ± 0.00	12.50 ± 0.00	2	Bactericidal
*K. pneumoniae* ATCC 700603	50.00 ± 0,00	12.50 ± 0.00	4	Bactericidal
*K. pneumoniae* ESBL+ MFBF 10690	20.83 ± 7.22	3.13 ± 0.00	~6.65	Bacteriostatic

MFBF—clinical isolates from the Collection of Microbes, University of Zagreb, Faculty of Pharmacy and Biochemistry Institute for Microbiology; MBC—minimal bactericidal concentration; MIC—minimal inhibitory concentration; $\bar{X}$ —mean; S.D.—standard deviation; N—number of experiments.

**Table 3:** MBC and MIC values for colloidal solutions of platinum nanoparticles for the tested bacterial strains of *E. coli* and *K. pneumoniae* in M9 minimal salts with 3% glucose solution as a medium. Concentrations are expressed as ppm (i.e., *parts per million*), equal to µg mL^−1^. Values are presented as means ± standard deviations.

Bacterial Strain	MBC/ppm	MIC/ppm	MBC/MIC Ratio	Antibacterial Effect (According to MBC/MIC Ratio) [55]
	$\bar{X}\pm S.D.\;\left(N=3\right)$	$\bar{X}\pm S.D.\;\left(N=3\right)$		
*E. coli* ATCC 10536	18.75 ± 0.00	12.5 ± 0.00	1.50	Bactericidal
*E. coli* ESBL+ MFBF 12795	25.00 ± 0.00	21.87 ± 4.41	~1.14	Bactericidal
*K. pneumoniae* ATCC 700603	37.5 ± 0.00	23.95 ± 1.80	~1.56	Bactericidal
*K. pneumoniae* ESBL+ MFBF 10690	25.00 ± 0.00	15.31 ± 4.41	~1.63	Bactericidal

MFBF—clinical isolates from Collection of Microbes, University of Zagreb, Faculty of Pharmacy and Biochemistry Institute for Microbiology; MBC—minimal bactericidal concentration; MIC—minimal inhibitory concentration; $\bar{X}$—mean; S.D.—standard deviation; N—number of experiments.

## Data Availability

Data are available upon request from the corresponding author.

## Supplementary Materials

The following supporting information can be downloaded at: https://www.mdpi.com/article/10.3390/pharmaceutics14081714/s1. Figures S1–S29: Graphical statistical analysis representation of determined ROS levels obtained for all tested bacterial strains exposed to 2 × MIC, MIC, and 1/2 × MIC of nPt from 0 to 24 hours of exposure.; Tables S1–S28: Statistical significance between comparing variables for the group of results from Figures S1–S29.; Figures S30–S58: Graphical statistical analysis representation of determined DNA/RNA concentrations obtained for all tested bacterial strains exposed to 2 × MIC, MIC, and 1/2 × MIC from 0 to 24 hours of exposure.; Tables S29–S56: Statistical significance between comparing variables for the group of results from Figures S30–S58.; Figures S59–S87: Graphical statistical analysis representation of determined protein concentrations obtained for all tested bacterial strains exposed to 2 × MIC, MIC, and 1/2 × MIC from 0 to 24 hours of exposure.; Tables S57–S84: Statistical significance between comparing variables for the group of results from Figures S59–S87.
