# Supplementary material for: Innovative Insights into In Vitro Activity of Colloidal Platinum Nanoparticles against ESBL-Producing Strains of Escherichia coli and Klebsiella pneumoniae
Source: Pharmaceutics. 2022 Aug 17;14(8):1714. doi: 10.3390/pharmaceutics14081714 (PMC9413765; doi:10.3390/pharmaceutics14081714)
Supplement: Supplementary file 1 [file pharmaceutics-14-01714-s001.zip › pharmaceutics-1824297-supplementary.pdf]

pharmaceutics

Supplementary Materials:

Innovative Insights into In Vitro Activity of Colloidal Platinum Nanoparticles against ESBL-Producing Strains of *Escherichia coli* and *Klebsiella pneumoniae*

Damir Vukoja, Josipa Vlainić, Vanja Ljolić Bilić, Lela Martinaga, Iva Rezić , Diana Brlek Gorski and Ivan Kosalec

Statistical analysis for methods of ROS level determination and cell leakage (determination of cellular DNA/RNA and proteins in extracellular fluid sample)

Abbreviations

ROS – reactive oxygen species
E. coli – Escherichia coli
ATCC – American type cell culture
nPt – platinum nanoparticles
MIC – minimal inhibitory concentration
ESBL – extended spectrum beta lactamase
MFBF – denotement for microbiological collection of Faculty of Pharmacy and Biochemistry University of Zagreb
K. pneumoniae – Klebsiella pneumoniae

1. ROS Level Determination Statistical Analysis

Statistical test used: Friedman test (nonparametric comparison of *n* samples)
Statistical significance value (α) = 0,05

1.1. E. coli ATCC 10536

1.1.1. At 0 Time Point (Of Exposure to nPt)

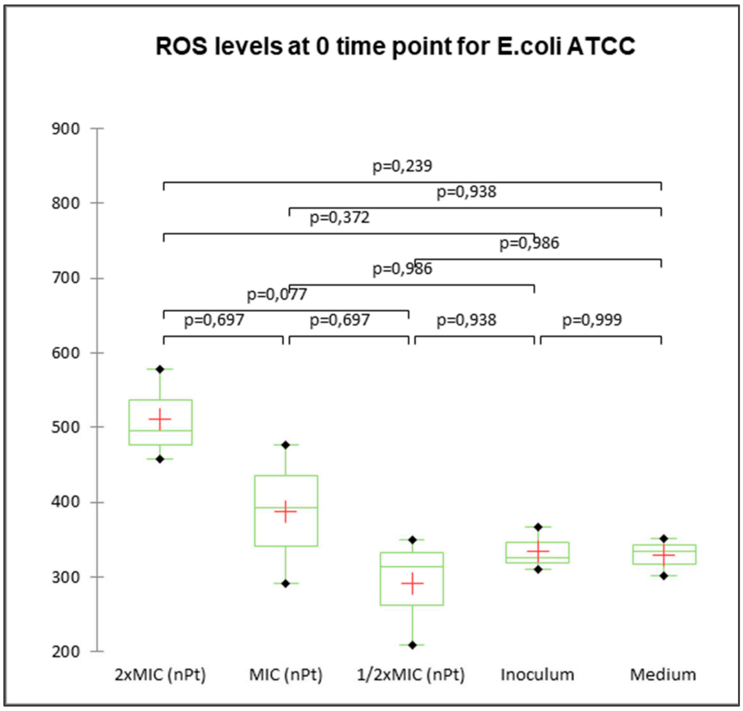

Figure S1. Graphical statistical analysis representation of determined ROS levels obtained for E coli ATCC 10536 exposed to 2 × MIC, MIC, and 1/2 × MIC of nPt at zero time point. P values are shown between comparing variables.

Table S1. Statistical significance between comparing variables for the group of results from Figure S1. If p < 0,05 the difference is considered as significant (Yes), if the p > 0,05 the difference is considered as not significant (No).

Significant differences:

	2xMIC (nPt)	MIC (nPt)	1/2xMIC (nPt)	Inoculum	Medium
2xMIC (nPt)	-	No	No	No	No
MIC (nPt)	No	-	No	No	No
1/2xMIC (nPt)	No	No	-	No	No
Inoculum	No	No	No	-	No
Medium	No	No	No	No	-

1.1.2. After 1 Hour (of Exposure to nPt)

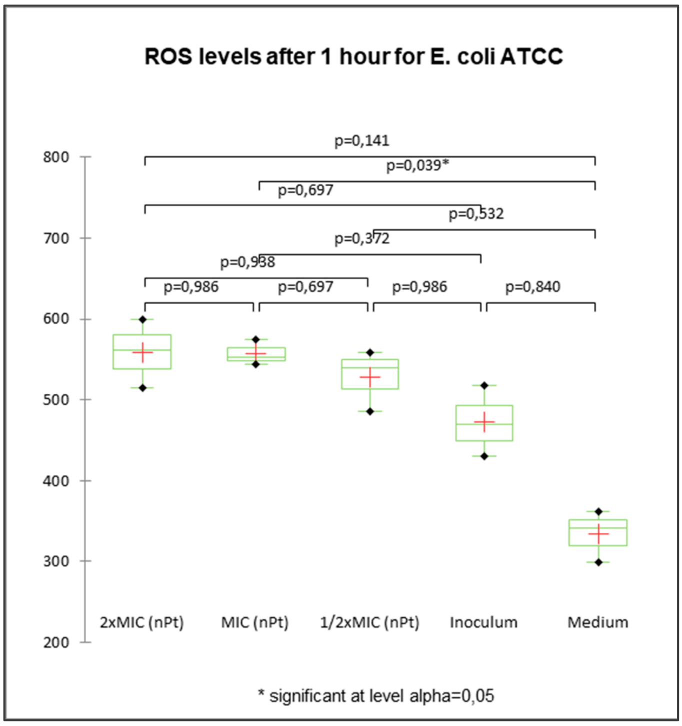

Figure S2. Graphical statistical analysis representation of determined ROS levels obtained for E coli ATCC 10536 exposed to 2 × MIC, MIC, and 1/2 × MIC of nPt after 1 hour of exposure. P values are shown between comparing variables.

Table S2. Statistical significance between comparing variables for the group of results from Figure S2. If p < 0,05 the difference is considered as significant (Yes), if the p > 0,05 the difference is considered as not significant (No).

Significant differences:

	2xMIC (nPt)	MIC (nPt)	1/2xMIC (nPt)	Inoculum	Medium
2xMIC (nPt)	-	No	No	No	No
MIC (nPt)	No	-	No	No	**Yes**
1/2xMIC (nPt)	No	No	-	No	No
Inoculum	No	No	No	-	No
Medium	No	**Yes**	No	No	-

1.1.3. After 3 hours (of Exposure to nPt)

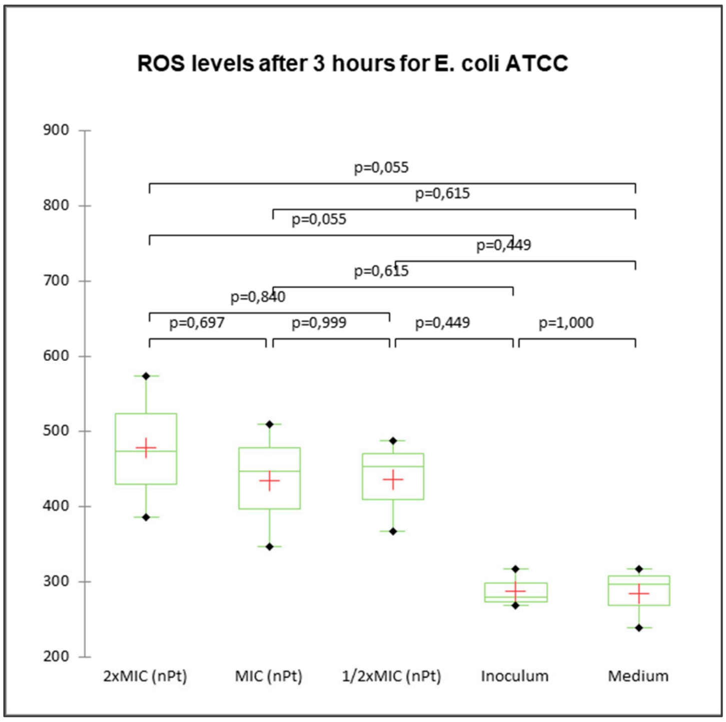

Figure S3. Graphical statistical analysis representation of determined ROS levels obtained for E coli ATCC 10536 exposed to 2 × MIC, MIC, and 1/2 × MIC of nPt after 3 hours of exposure. P values are shown between comparing variables.

Table S3. Statistical significance between comparing variables for the group of results from Figure S3. If $p < 0{,}05$ the difference is considered as significant (Yes), if the $p > 0{,}05$ the difference is considered as not significant (No).

Significant differences:					
	2xMIC (nPt)	MIC (nPt)	1/2xMIC (nPt)	Inoculum	Medium
2xMIC (nPt)	-	No	No	No	No
MIC (nPt)	No	-	No	No	No
1/2xMIC (nPt)	No	No	-	No	No
Inoculum	No	No	No	-	No
Medium	No	No	No	No	-

1.1.4. After 6 hours (of exposure to nPt).

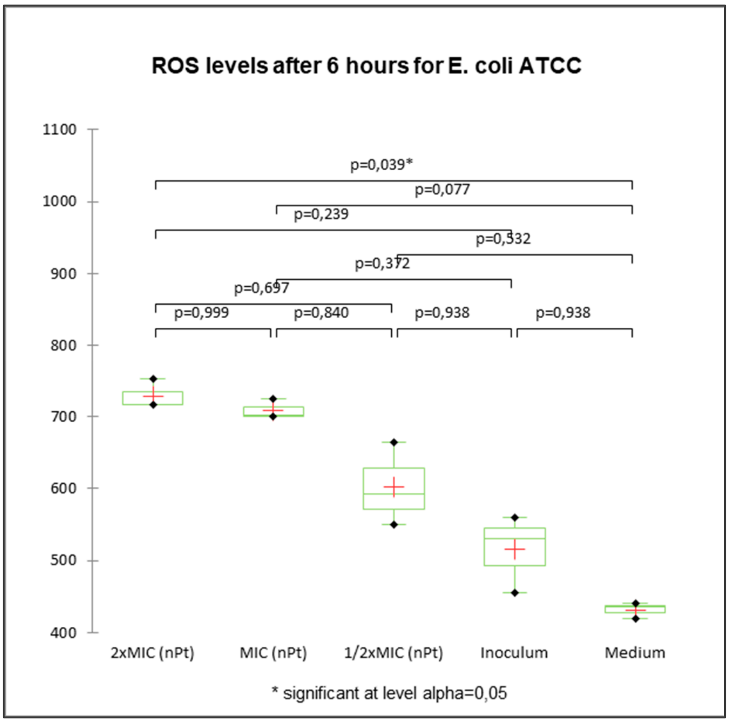

Figure S4. Graphical statistical analysis representation of determined ROS levels obtained for E coli ATCC 10536 exposed to 2 × MIC, MIC, and 1/2 × MIC of nPt after 6 hours of exposure. P values are shown between comparing variables.

Table S4. Statistical significance between comparing variables for the group of results from Figure S4. If p < 0,05 the difference is considered as significant (Yes), if the p > 0,05 the difference is considered as not significant (No).

Significant differences:

	2xMIC (nPt)	MIC (nPt)	1/2xMIC (nPt)	Inoculum	Medium
2xMIC (nPt)	-	No	No	No	**Yes**
MIC (nPt)	No	-	No	No	No
1/2xMIC (nPt)	No	No	-	No	No
Inoculum	No	No	No	-	No
Medium	**Yes**	No	No	No	-

1.1.5. After 12 hours (of exposure to nPt)

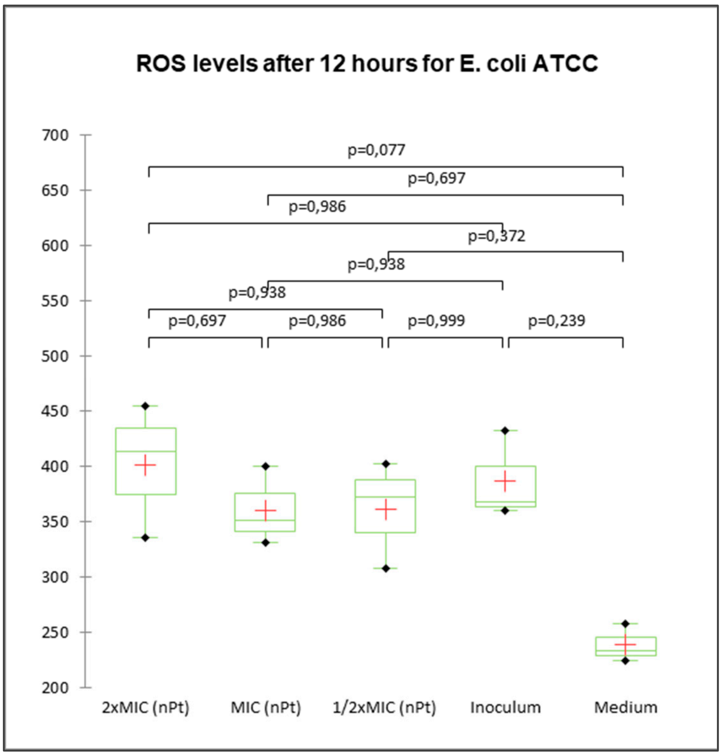

Figure S5. Graphical statistical analysis representation of determined ROS levels obtained for E coli ATCC 10536 exposed to 2 × MIC, MIC, and 1/2 × MIC of nPt after 12 hours of exposure. P values are shown between comparing variables.

Table S5. Statistical significance between comparing variables for the group of results from Figure S5. If $p < 0,05$ the difference is considered as significant (Yes), if the $p > 0,05$ the difference is considered as not significant (No).

Significant differences:

	2xMIC (nPt)	MIC (nPt)	1/2xMIC (nPt)	Inoculum	Medium
2xMIC (nPt)	-	No	No	No	No
MIC (nPt)	No	-	No	No	No
1/2xMIC (nPt)	No	No	-	No	No
Inoculum	No	No	No	-	No
Medium	No	No	No	No	-

1.1.6. After 24 hours (of exposure to nPt)

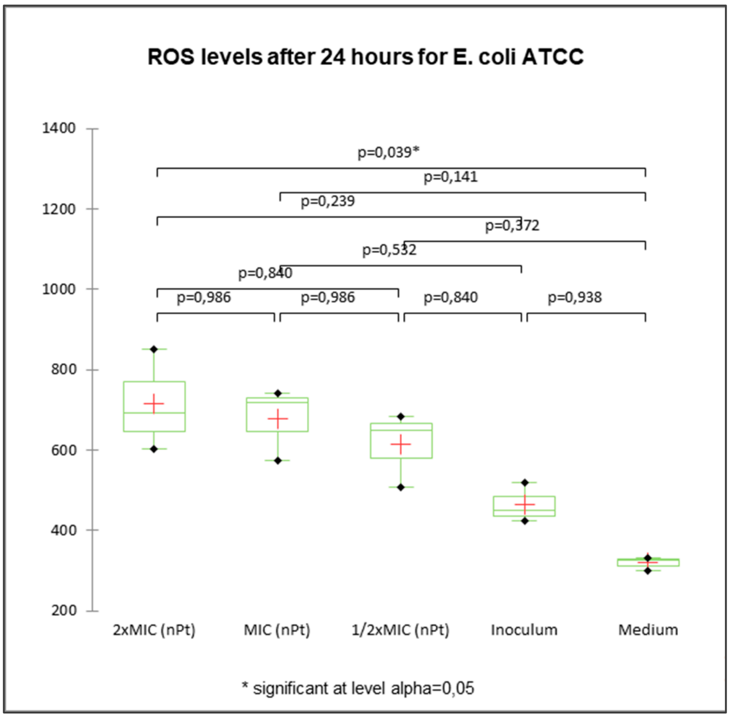

Figure S6. Graphical statistical analysis representation of determined ROS levels obtained for E coli ATCC 10536 exposed to 2 × MIC, MIC, and 1/2 × MIC of nPt after 24 hours of exposure. P values are shown between comparing variables.

Table S6. Statistical significance between comparing variables for the group of results from Figure S6. If p < 0,05 the difference is considered as significant (Yes), if the p > 0,05 the difference is considered as not significant (No).

Significant differences:

	2xMIC (nPt)	MIC (nPt)	1/2xMIC (nPt)	Inoculum	Medium
2xMIC (nPt)	-	No	No	No	**Yes**
MIC (nPt)	No	-	No	No	No
1/2xMIC (nPt)	No	No	-	No	No
Inoculum	No	No	No	-	No
Medium	**Yes**	No	No	No	-

1.1.7. Total ROS levels statistical count (sum of results of all measurement time points)

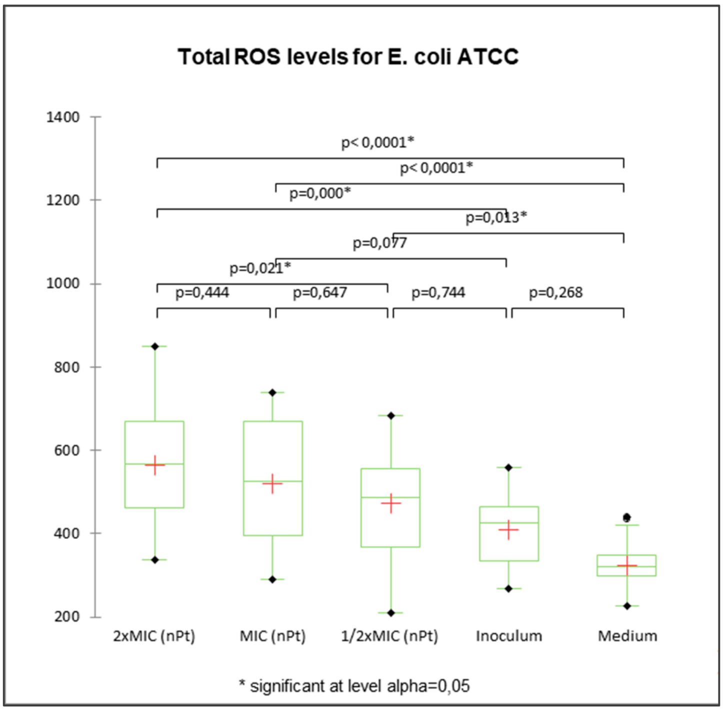

Figure S7. Graphical statistical analysis representation of determined total ROS levels obtained for E coli ATCC 10536 exposed to 2 × MIC, MIC, and 1/2 × MIC of nPt as a sum of results of all measurement time points. P values are shown between comparing variables.

Table S7. Statistical significance between comparing variables for the group of results from Figure S7. If p < 0,05 the difference is considered as significant (Yes), if the p > 0,05 the difference is considered as not significant (No).

Significant differences:					
	2xMIC (nPt)	MIC (nPt)	1/2xMIC (nPt)	Inoculum	Medium
2xMIC (nPt)	-	No	**Yes**	**Yes**	**Yes**
MIC (nPt)	No	-	No	No	**Yes**
1/2xMIC (nPt)	**Yes**	No	-	No	**Yes**
Inoculum	**Yes**	No	No	-	No
Medium	**Yes**	**Yes**	**Yes**	No	-

1.2. E. coli ESBL+ MFBF 12795

1.2.1. At 0 time point (of exposure to nPt)

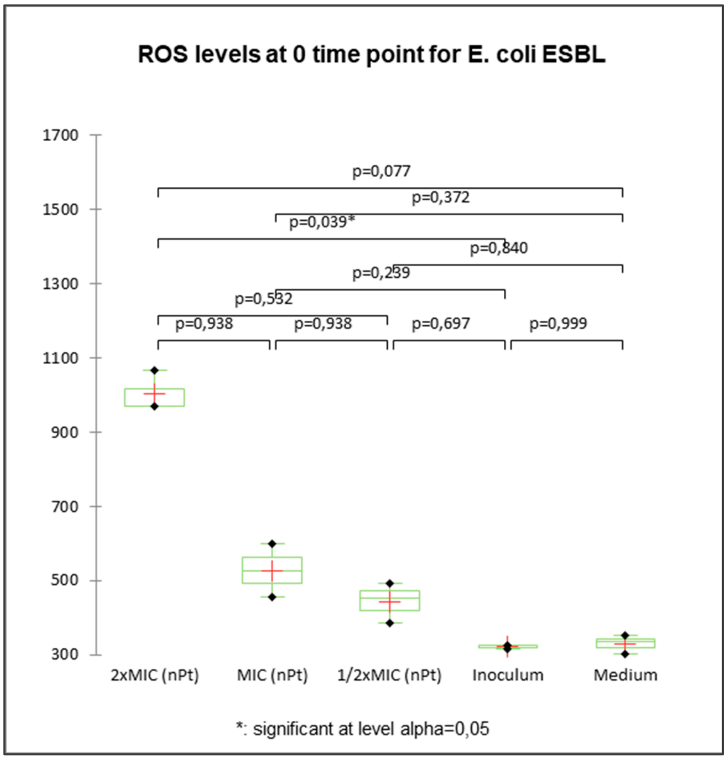

Figure S8. Graphical statistical analysis representation of determined ROS levels obtained for E coli ESBL+ MFBF 12795 exposed to 2 × MIC, MIC, and 1/2 × MIC of nPt at zero time point. P values are shown between comparing variables.

Table S8. Statistical significance between comparing variables for the group of results from Figure S8. If p < 0,05 the difference is considered as significant (Yes), if the p > 0,05 the difference is considered as not significant (No).

Significant differences:

	2xMIC (nPt)	MIC (nPt)	1/2xMIC (nPt)	Inoculum	Medium
2xMIC (nPt)	-	No	No	**Yes**	No
MIC (nPt)	No	-	No	No	No
1/2xMIC (nPt)	No	No	-	No	No
Inoculum	**Yes**	No	No	-	No
Medium	No	No	No	No	-

1.2.2. After 1 hour (of exposure to nPt)

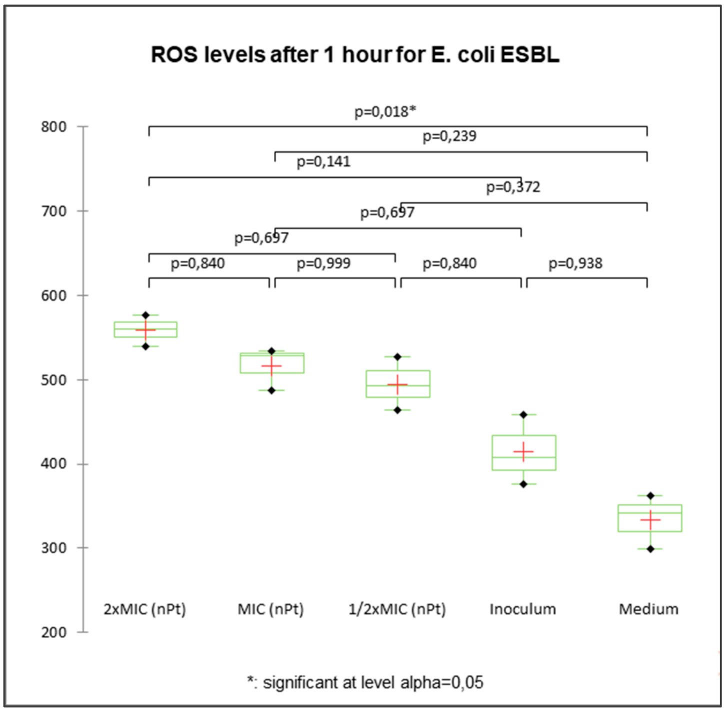

Figure S9. Graphical statistical analysis representation of determined ROS levels obtained for E coli ESBL+ MFBF 12795 exposed to 2 × MIC, MIC, and 1/2 × MIC of nPt after 1 hour of exposure. P values are shown between comparing variables.

Table S9. Statistical significance between comparing variables for the group of results from Figure S9. If p < 0,05 the difference is considered as significant (Yes), if the p > 0,05 the difference is considered as not significant (No).

Significant differences:					
	2xMIC (nPt)	MIC (nPt)	1/2xMIC (nPt)	Inoculum	Medium
2xMIC (nPt)	-	No	No	No	**Yes**
MIC (nPt)	No	-	No	No	No
1/2xMIC (nPt)	No	No	-	No	No
Inoculum	No	No	No	-	No
Medium	**Yes**	No	No	No	-

1.2.3. After 3 hours (of exposure to nPt)

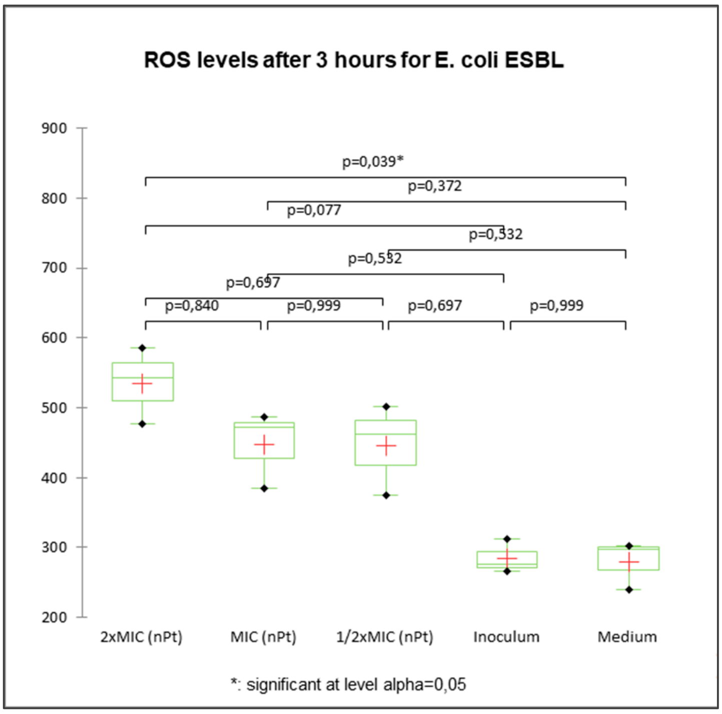

Figure S10. Graphical statistical analysis representation of determined ROS levels obtained for E coli ESBL+ MFBF 12795 exposed to 2 × MIC, MIC, and 1/2 × MIC of nPt after 3 hours of exposure. P values are shown between comparing variables.

Table S10. Statistical significance between comparing variables for the group of results from Figure S10. If p < 0,05 the difference is considered as significant (Yes), if the p > 0,05 the difference is considered as not significant (No).

Significant differences:

	2xMIC (nPt)	MIC (nPt)	1/2xMIC (nPt)	Inoculum	Medium
2xMIC (nPt)	-	No	No	No	**Yes**
MIC (nPt)	No	-	No	No	No
1/2xMIC (nPt)	No	No	-	No	No
Inoculum	No	No	No	-	No
Medium	**Yes**	No	No	No	-

1.2.4. After 6 hours (of exposure to nPt)

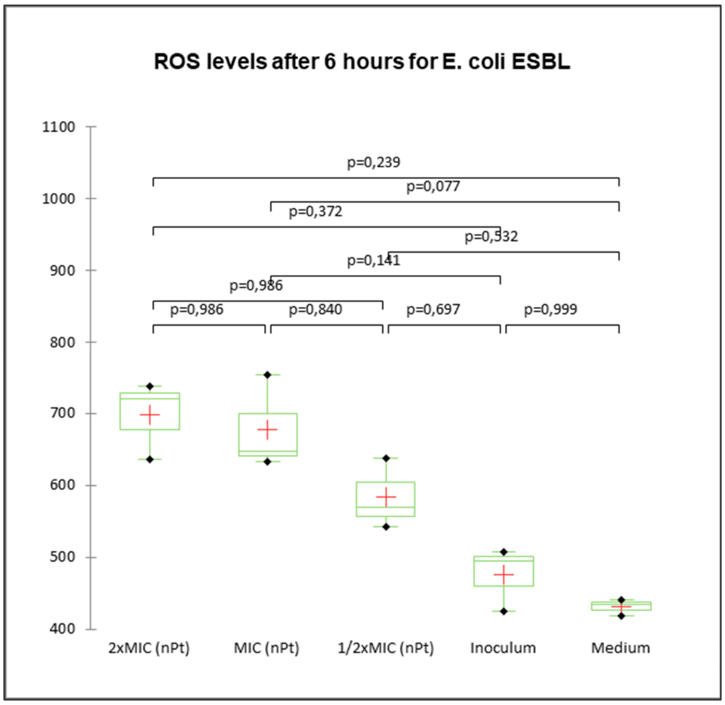

Figure S11. Graphical statistical analysis representation of determined ROS levels obtained for E coli ESBL+ MFBF 12795 exposed to 2 × MIC, MIC, and 1/2 × MIC of nPt after 6 hours of exposure. P values are shown between comparing variables.

Table S11. Statistical significance between comparing variables for the group of results from Figure S11. If p < 0,05 the difference is considered as significant (Yes), if the p > 0,05 the difference is considered as not significant (No).

Significant differences:					
	2xMIC (nPt)	MIC (nPt)	1/2xMIC (nPt)	Inoculum	Medium
2xMIC (nPt)	-	No	No	No	No
MIC (nPt)	No	-	No	No	No
1/2xMIC (nPt)	No	No	-	No	No
Inoculum	No	No	No	-	No
Medium	No	No	No	No	-

1.2.5. After 12 hours (of exposure to nPt)

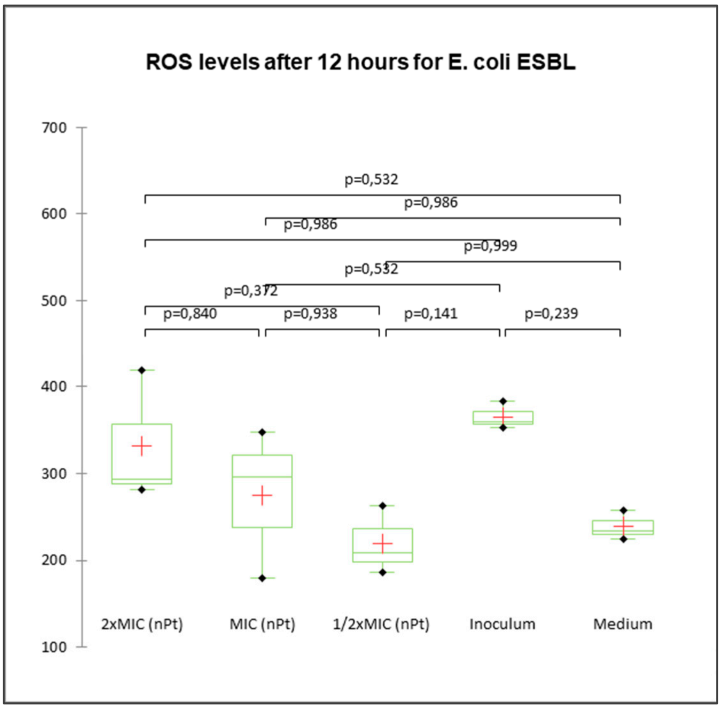

Figure S12. Graphical statistical analysis representation of determined ROS levels obtained for E coli ESBL+ MFBF 12795 exposed to 2 × MIC, MIC, and 1/2 × MIC of nPt after 12 hours of exposure. P values are shown between comparing variables.

Table S12. Statistical significance between comparing variables for the group of results from Figure S12. If p < 0,05 the difference is considered as significant (Yes), if the p > 0,05 the difference is considered as not significant (No).

Significant differences:					
	2xMIC (nPt)	MIC (nPt)	1/2xMIC (nPt)	Inoculum	Medium
2xMIC (nPt)	-	No	No	No	No
MIC (nPt)	No	-	No	No	No
1/2xMIC (nPt)	No	No	-	No	No
Inoculum	No	No	No	-	No
Medium	No	No	No	No	-

1.2.6. After 24 hours (of exposure to nPt)

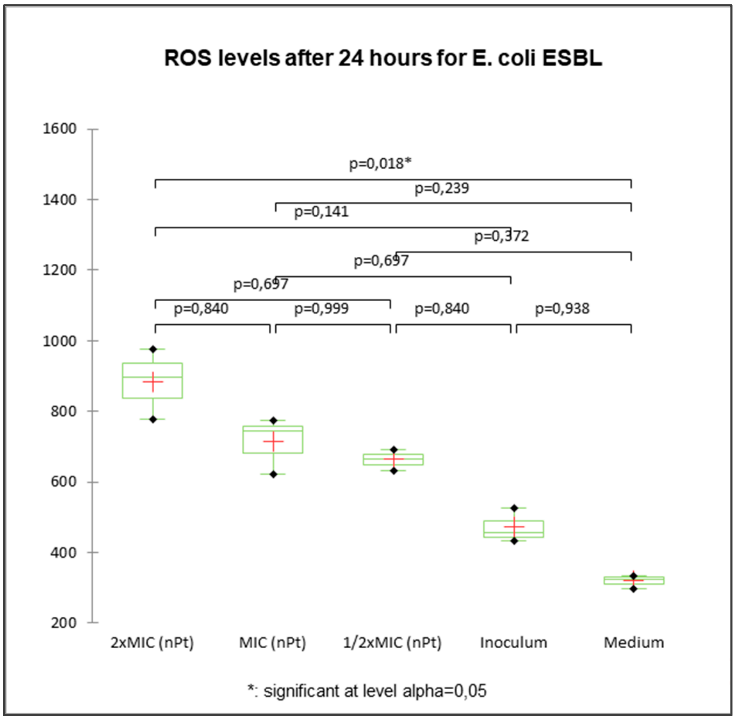

Figure S13. Graphical statistical analysis representation of determined ROS levels obtained for E coli ESBL+ MFBF 12795 exposed to 2 × MIC, MIC, and 1/2 × MIC of nPt after 24 hours of exposure. P values are shown between comparing variables.

Table S13. Statistical significance between comparing variables for the group of results from Figure S13. If p < 0,05 the difference is considered as significant (Yes), if the p > 0,05 the difference is considered as not significant (No).

Significant differences:

	2xMIC (nPt)	MIC (nPt)	1/2xMIC (nPt)	Inoculum	Medium
2xMIC (nPt)	-	No	No	No	**Yes**
MIC (nPt)	No	-	No	No	No
1/2xMIC (nPt)	No	No	-	No	No
Inoculum	No	No	No	-	No
Medium	**Yes**	No	No	No	-

1.2.7. Total ROS levels statistical count (sum of results of all measurement time points)

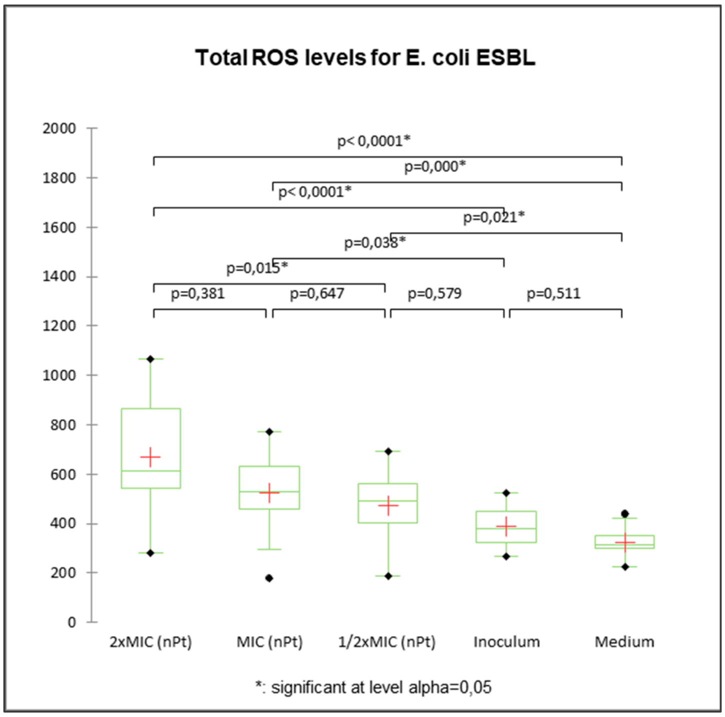

Figure S14. Graphical statistical analysis representation of determined total ROS levels obtained for E coli ESBL+ MFBF 12795 exposed to 2 × MIC, MIC, and 1/2 × MIC of nPt as a sum of results of all measurement time points. P values are shown between comparing variables.

Table S14. Statistical significance between comparing variables for the group of results from Figure S14. If $p < 0,05$ the difference is considered as significant (Yes), if the $p > 0,05$ the difference is considered as not significant (No).

Significant differences:					
	2xMIC (nPt)	MIC (nPt)	1/2xMIC (nPt)	Inoculum	Medium
2xMIC (nPt)	-	No	**Yes**	**Yes**	**Yes**
MIC (nPt)	No	-	No	**Yes**	**Yes**
1/2xMIC (nPt)	**Yes**	No	-	No	**Yes**
Inoculum	**Yes**	**Yes**	No	-	No
Medium	**Yes**	**Yes**	**Yes**	No	-

1.3. K. pneumoniae ATCC 700603

1.3.1. At 0 time point (of exposure to nPt)

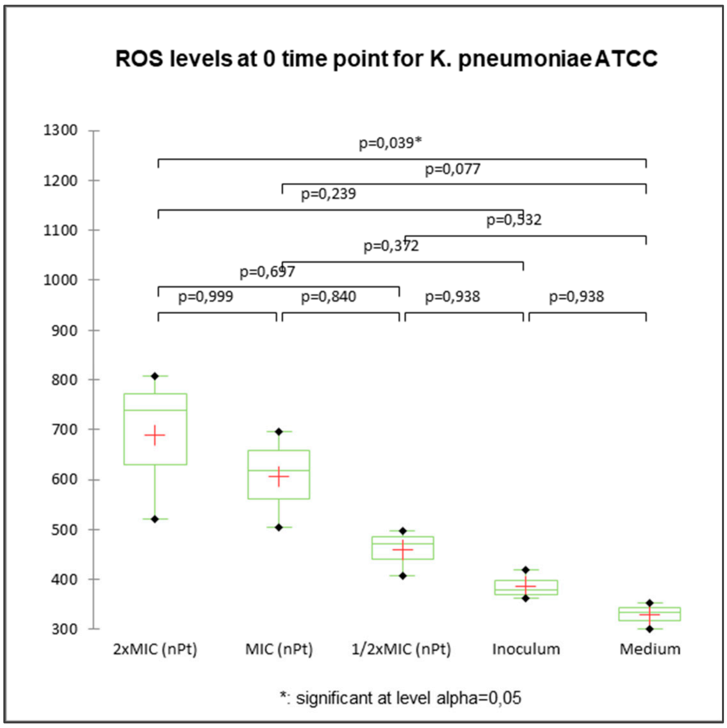

Figure S15. Graphical statistical analysis representation of determined ROS levels obtained for K pneumoniae ATCC 700603 exposed to 2 × MIC, MIC, and 1/2 × MIC of nPt at zero time point. P values are shown between comparing variables.

Table S15. Statistical significance between comparing variables for the group of results from Figure S15. If p < 0,05 the difference is considered as significant (Yes), if the p > 0,05 the difference is considered as not significant (No).

Significant differences:					
	2xMIC (nPt)	MIC (nPt)	1/2xMIC (nPt)	Inoculum	Medium
2xMIC (nPt)	-	No	No	No	**Yes**
MIC (nPt)	No	-	No	No	No
1/2xMIC (nPt)	No	No	-	No	No
Inoculum	No	No	No	-	No
Medium	**Yes**	No	No	No	-

1.3.2. After 1 hour (of exposure to nPt)

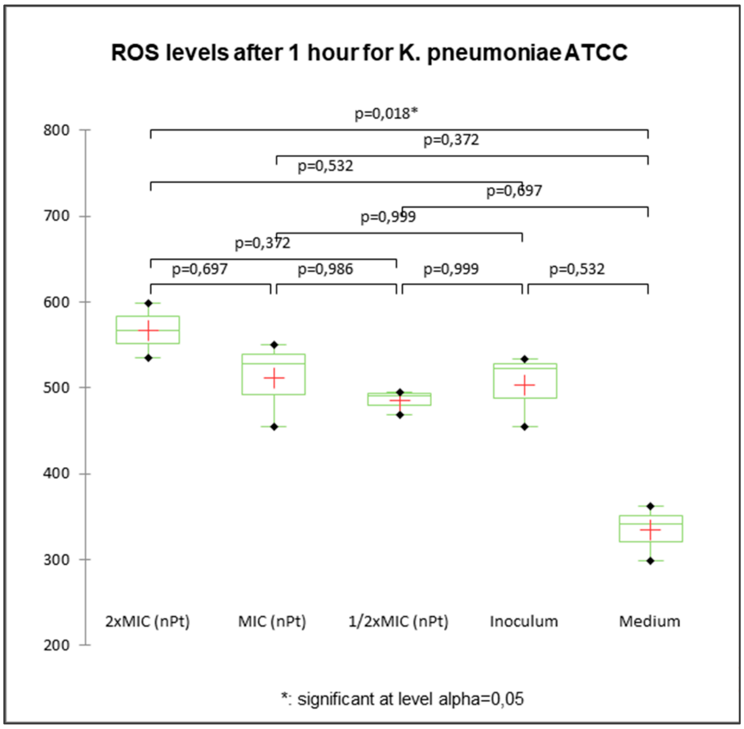

Figure S16. Graphical statistical analysis representation of determined ROS levels obtained for K pneumoniae ATCC 700603 exposed to 2 × MIC, MIC, and 1/2 × MIC of nPt after 1 hour of exposure. P values are shown between comparing variables.

Table S16. Statistical significance between comparing variables for the group of results from Figure S16. If $p < 0,05$ the difference is considered as significant (Yes), if the $p > 0,05$ the difference is considered as not significant (No).

Significant differences:					
	2xMIC (nPt)	MIC (nPt)	1/2xMIC (nPt)	Inoculum	Medium
2xMIC (nPt)	-	No	No	No	**Yes**
MIC (nPt)	No	-	No	No	No
1/2xMIC (nPt)	No	No	-	No	No
Inoculum	No	No	No	-	No
Medium	**Yes**	No	No	No	-

1.3.3. After 3 hours (of exposure to nPt)

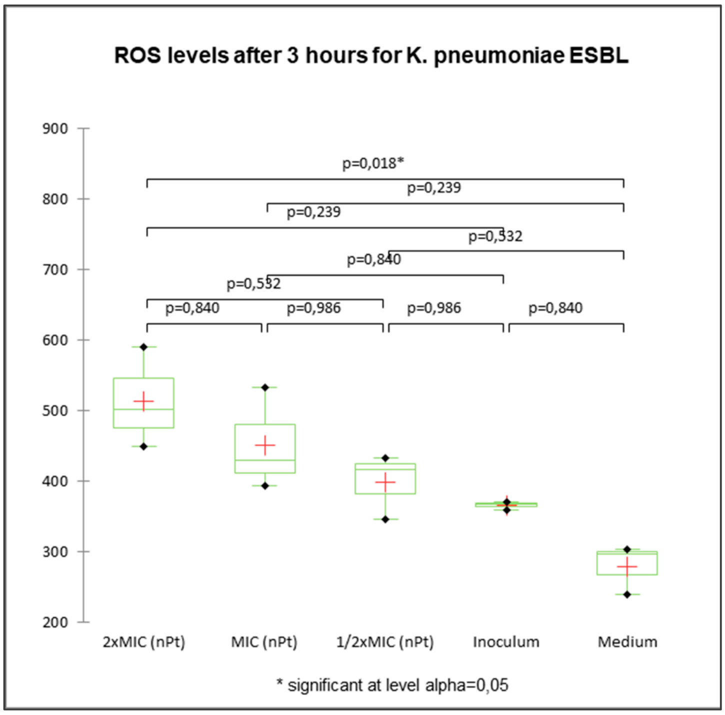

Figure S17. Graphical statistical analysis representation of determined ROS levels obtained for K pneumoniae ATCC 700603 exposed to 2 × MIC, MIC, and 1/2 × MIC of nPt after 3 hours of exposure. P values are shown between comparing variables.

Table S17. Statistical significance between comparing variables for the group of results from Figure S17. If p < 0,05 the difference is considered as significant (Yes), if the p > 0,05 the difference is considered as not significant (No).

Significant differences:					
	2xMIC (nPt)	MIC (nPt)	1/2xMIC (nPt)	Inoculum	Medium
2xMIC (nPt)	-	No	No	No	**Yes**
MIC (nPt)	No	-	No	No	No
1/2xMIC (nPt)	No	No	-	No	No
Inoculum	No	No	No	-	No
Medium	**Yes**	No	No	No	-

1.3.4. After 6 hours (of exposure to nPt)

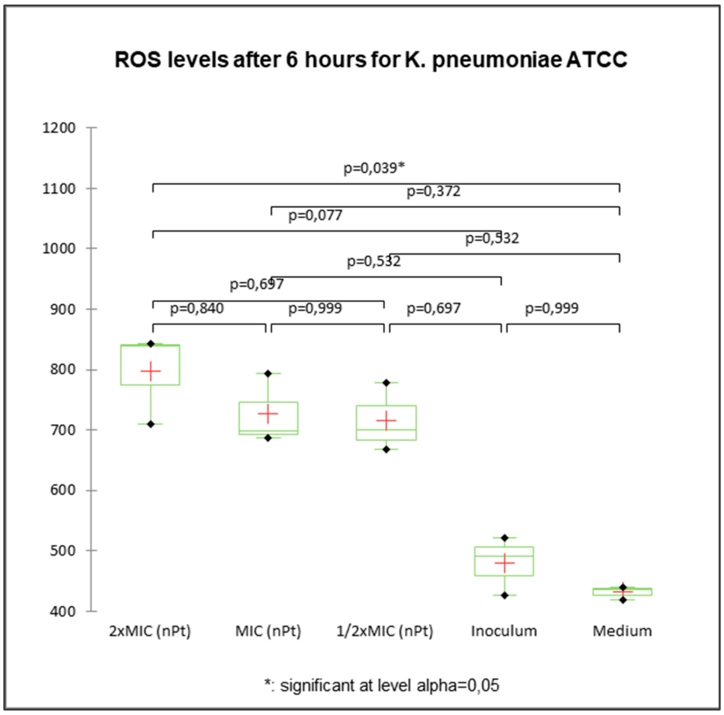

Figure S18. Graphical statistical analysis representation of determined ROS levels obtained for K pneumoniae ATCC 700603 exposed to 2 × MIC, MIC, and 1/2 × MIC of nPt after 6 hours of exposure. P values are shown between comparing variables.

Table S18. Statistical significance between comparing variables for the group of results from Figure S18. If p < 0,05 the difference is considered as significant (Yes), if the p > 0,05 the difference is considered as not significant (No).

Significant differences:					
	2xMIC (nPt)	MIC (nPt)	1/2xMIC (nPt)	Inoculum	Medium
2xMIC (nPt)	-	No	No	No	**Yes**
MIC (nPt)	No	-	No	No	No
1/2xMIC (nPt)	No	No	-	No	No
Inoculum	No	No	No	-	No
Medium	**Yes**	No	No	No	-

1.3.5. After 12 hours (of exposure to nPt)

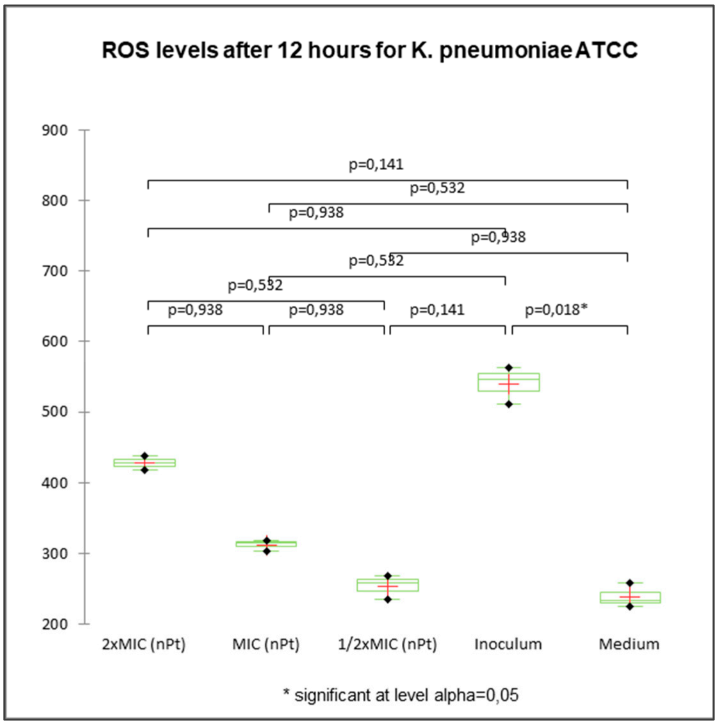

Figure S19. Graphical statistical analysis representation of determined ROS levels obtained for K pneumoniae ATCC 700603 exposed to 2 × MIC, MIC, and 1/2 × MIC of nPt after 12 hours of exposure. P values are shown between comparing variables.

Table S19. Statistical significance between comparing variables for the group of results from Figure S19. If p < 0,05 the difference is considered as significant (Yes), if the p > 0,05 the difference is considered as not significant (No).

Significant differences:					
	2xMIC (nPt)	MIC (nPt)	1/2xMIC (nPt)	Inoculum	Medium
2xMIC (nPt)	-	No	No	No	No
MIC (nPt)	No	-	No	No	No
1/2xMIC (nPt)	No	No	-	No	No
Inoculum	No	No	No	-	**Yes**
Medium	No	No	No	**Yes**	-

1.3.6. After 24 hours (of exposure to nPt)

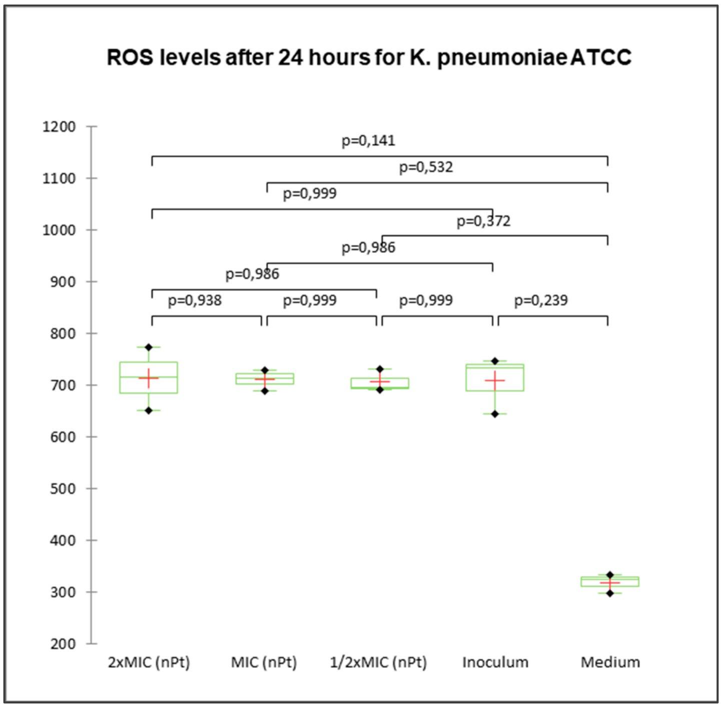

Figure S20. Figure S20. Graphical statistical analysis representation of determined ROS levels obtained for K pneumoniae ATCC 700603 exposed to 2 × MIC, MIC, and 1/2 × MIC of nPt after 24 hours of exposure. P values are shown between comparing variables.

Table S20. Statistical significance between comparing variables for the group of results from Figure S20. If p < 0,05 the difference is considered as significant (Yes), if the p > 0,05 the difference is considered as not significant (No).

Significant differences:

	2xMIC (nPt)	MIC (nPt)	1/2xMIC (nPt)	Inoculum	Medium
2xMIC (nPt)	-	No	No	No	No
MIC (nPt)	No	-	No	No	No
1/2xMIC (nPt)	No	No	-	No	No
Inoculum	No	No	No	-	No
Medium	No	No	No	No	-

1.3.7. Total ROS levels statistical count (sum of results of all measurement time points)

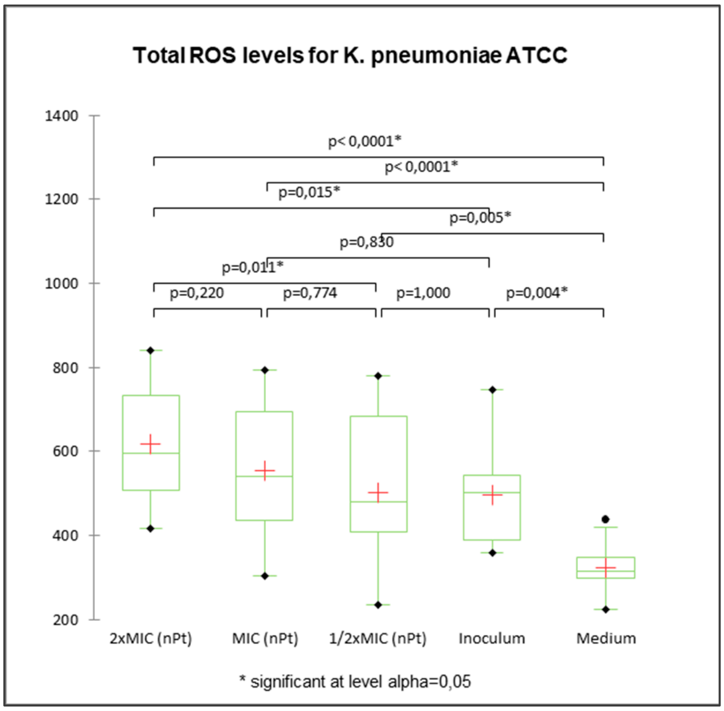

Figure S21. Graphical statistical analysis representation of determined total ROS levels obtained for K. pneumoniae ATCC 700603 exposed to 2 × MIC, MIC, and 1/2 × MIC of nPt as a sum of results of all measurement time points. P values are shown between comparing variables.

Table S21. Statistical significance between comparing variables for the group of results from Figure S21. If p < 0,05 the difference is considered as significant (Yes), if the p > 0,05 the difference is considered as not significant (No).

Significant differences:

	2xMIC (nPt)	MIC (nPt)	1/2xMIC (nPt)	Inoculum	Medium
2xMIC (nPt)	-	No	**Yes**	**Yes**	**Yes**
MIC (nPt)	No	-	No	No	**Yes**
1/2xMIC (nPt)	**Yes**	No	-	No	**Yes**
Inoculum	**Yes**	No	No	-	**Yes**
Medium	**Yes**	**Yes**	**Yes**	**Yes**	-

1.4. K. pneumoniae ESBL+ MFBF 10690

1.4.1. At 0 time point (of exposure to nPt)

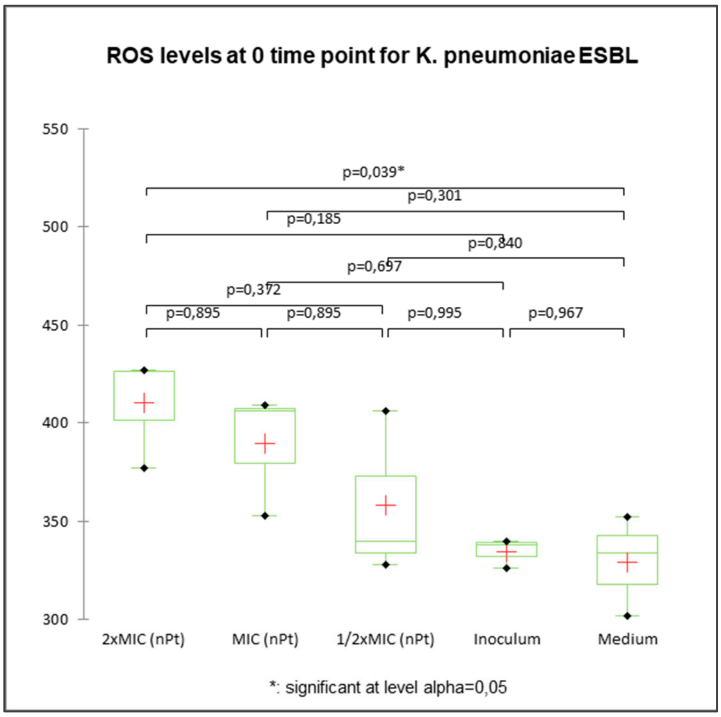

Figure S22 Graphical statistical analysis representation of determined ROS levels obtained for K pneumoniae ESBL+ MFBF 10690 exposed to 2 × MIC, MIC, and 1/2 × MIC of nPt at zero time point. P values are shown between comparing variables.

Table S22 Statistical significance between comparing variables for the group of results from Figure S22. If p < 0,05 the difference is considered as significant (Yes), if the p > 0,05 the difference is considered as not significant (No).

Significant differences:					
	2xMIC (nPt)	MIC (nPt)	1/2xMIC (nPt)	Inoculum	Medium
2xMIC (nPt)	-	No	No	No	**Yes**
MIC (nPt)	No	-	No	No	No
1/2xMIC (nPt)	No	No	-	No	No
Inoculum	No	No	No	-	No
Medium	**Yes**	No	No	No	-

1.4.2. After 1 hour (of exposure to nPt)

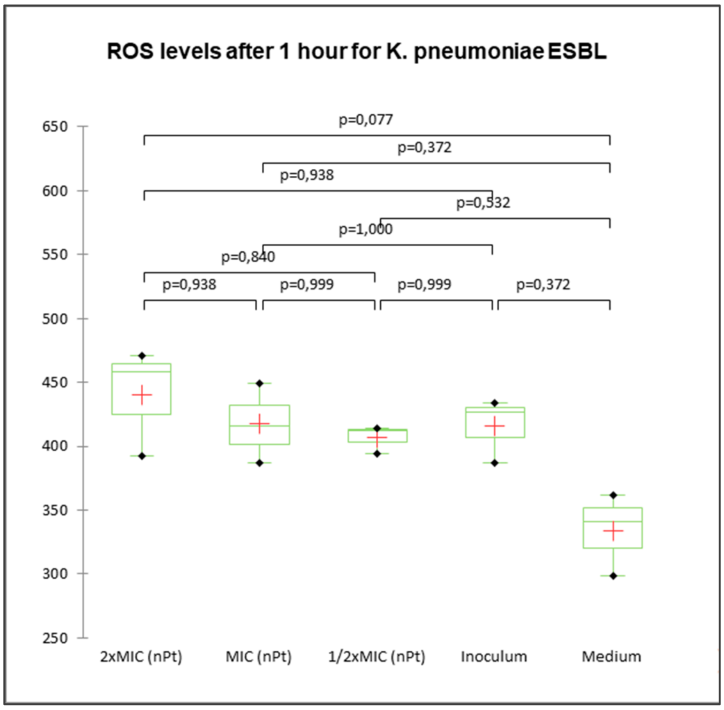

Figure S23. Graphical statistical analysis representation of determined ROS levels obtained for K pneumoniae ESBL+ MFBF 10690 exposed to 2 × MIC, MIC, and 1/2 × MIC of nPt after 1 hour of exposure. P values are shown between comparing variables.

Table S23. Statistical significance between comparing variables for the group of results from Figure S23. If p < 0,05 the difference is considered as significant (Yes), if the p > 0,05 the difference is considered as not significant (No).

Significant differences:					
	2xMIC (nPt)	MIC (nPt)	1/2xMIC (nPt)	Inoculum	Medium
2xMIC (nPt)	-	No	No	No	No
MIC (nPt)	No	-	No	No	No
1/2xMIC (nPt)	No	No	-	No	No
Inoculum	No	No	No	-	No
Medium	No	No	No	No	-

1.4.3. After 3 hours (of exposure to nPt)

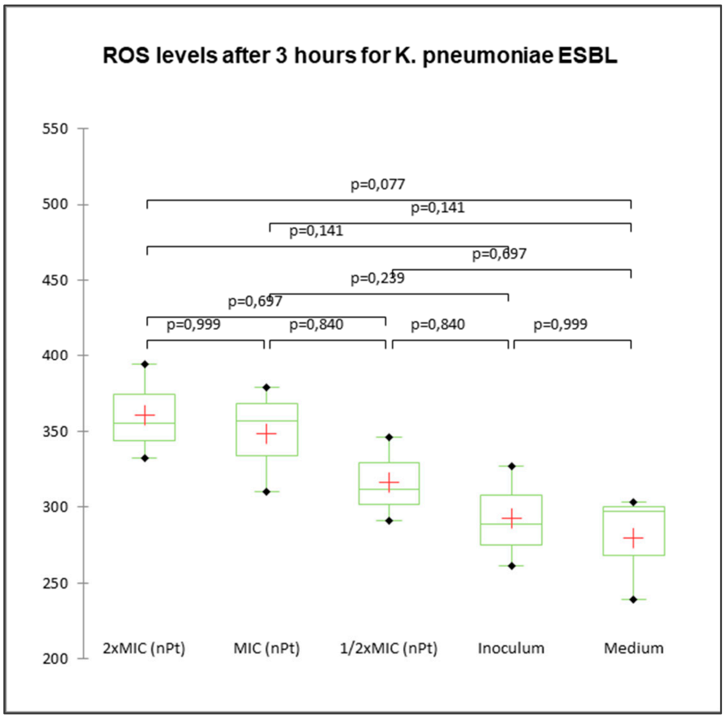

Figure S24. Graphical statistical analysis representation of determined ROS levels obtained for K pneumoniae ESBL+ MFBF 10690 exposed to 2 × MIC, MIC, and 1/2 × MIC of nPt after 3 hours of exposure. P values are shown between comparing variables.

Table S24. Statistical significance between comparing variables for the group of results from Figure S24. If p < 0,05 the difference is considered as significant (Yes), if the p > 0,05 the difference is considered as not significant (No).

Significant differences:					
	2xMIC (nPt)	MIC (nPt)	1/2xMIC (nPt)	Inoculum	Medium
2xMIC (nPt)	-	No	No	No	No
MIC (nPt)	No	-	No	No	No
1/2xMIC (nPt)	No	No	-	No	No
Inoculum	No	No	No	-	No
Medium	No	No	No	No	-

1.4.4. After 6 hours (of exposure to nPt)

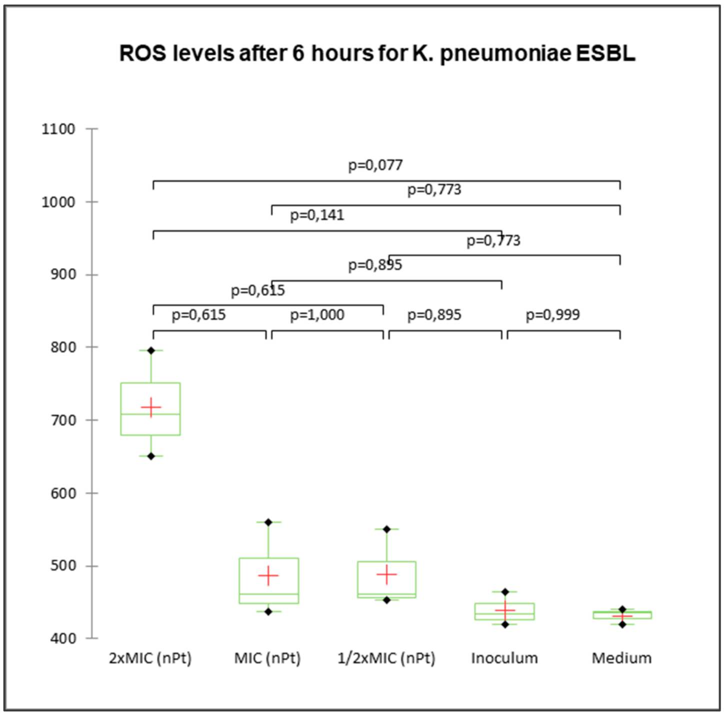

Figure S25. Graphical statistical analysis representation of determined ROS levels obtained for K pneumoniae ESBL+ MFBF 10690 exposed to 2 × MIC, MIC, and 1/2 × MIC of nPt after 6 hours of exposure. P values are shown between comparing variables.

Table S25. Statistical significance between comparing variables for the group of results from Figure S25. If p < 0,05 the difference is considered as significant (Yes), if the p > 0,05 the difference is considered as not significant (No).

Significant differences:

	2xMIC (nPt)	MIC (nPt)	1/2xMIC (nPt)	Inoculum	Medium
2xMIC (nPt)	-	No	No	No	No
MIC (nPt)	No	-	No	No	No
1/2xMIC (nPt)	No	No	-	No	No
Inoculum	No	No	No	-	No
Medium	No	No	No	No	-

1.4.5. After 12 hours (of exposure to nPt)

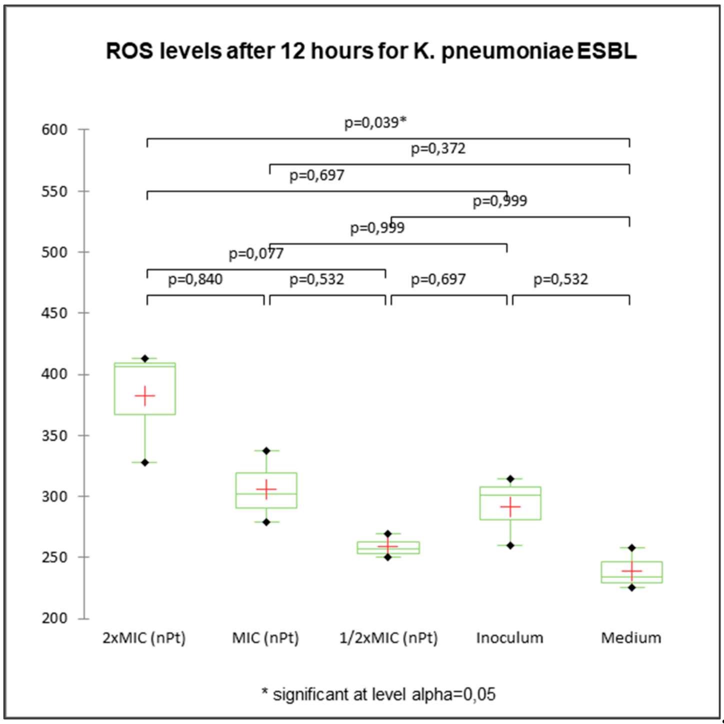

Figure S26. Graphical statistical analysis representation of determined ROS levels obtained for K pneumoniae ESBL+ MFBF 10690 exposed to 2 × MIC, MIC, and 1/2 × MIC of nPt after 12 hours of exposure. P values are shown between comparing variables.

Table S26. Statistical significance between comparing variables for the group of results from Figure S26. If p < 0,05 the difference is considered as significant (Yes), if the p > 0,05 the difference is considered as not significant (No).

Significant differences:

	2xMIC (nPt)	MIC (nPt)	1/2xMIC (nPt)	Inoculum	Medium
2xMIC (nPt)	-	No	No	No	**Yes**
MIC (nPt)	No	-	No	No	No
1/2xMIC (nPt)	No	No	-	No	No
Inoculum	No	No	No	-	No
Medium	**Yes**	No	No	No	-

1.4.6. After 24 hours (of exposure to nPt)

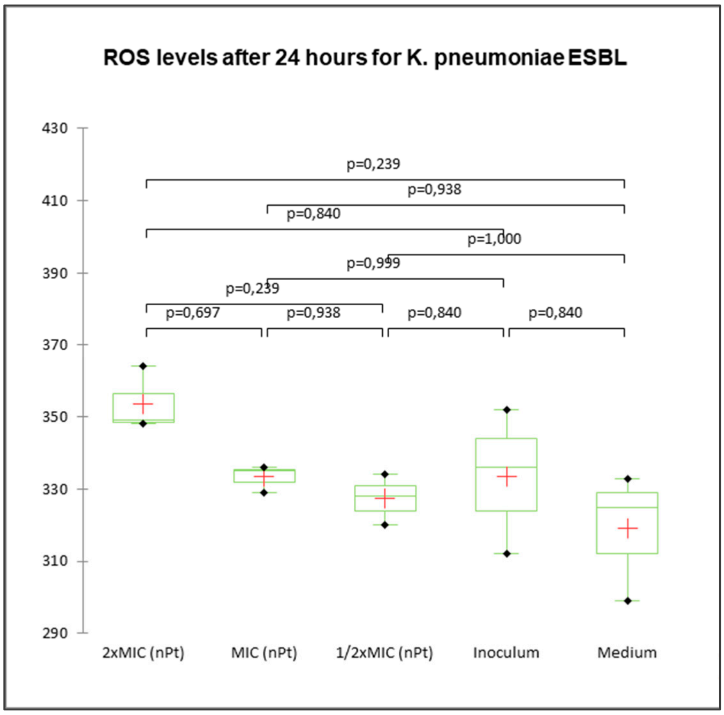

Figure S27. Graphical statistical analysis representation of determined ROS levels obtained for K pneumoniae ESBL+ MFBF 10690 exposed to 2 × MIC, MIC, and 1/2 × MIC of nPt after 24 hours of exposure. P values are shown between comparing variables.

Table S27. Statistical significance between comparing variables for the group of results from Figure S27. If p < 0,05 the difference is considered as significant (Yes), if the p > 0,05 the difference is considered as not significant (No).

Significant differences:					
	2xMIC (nPt)	MIC (nPt)	1/2xMIC (nPt)	Inoculum	Medium
2xMIC (nPt)	-	No	No	No	No
MIC (nPt)	No	-	No	No	No
1/2xMIC (nPt)	No	No	-	No	No
Inoculum	No	No	No	-	No
Medium	No	No	No	No	-

1.4.7. Total ROS levels statistical count (sum of results of all measurement time points)

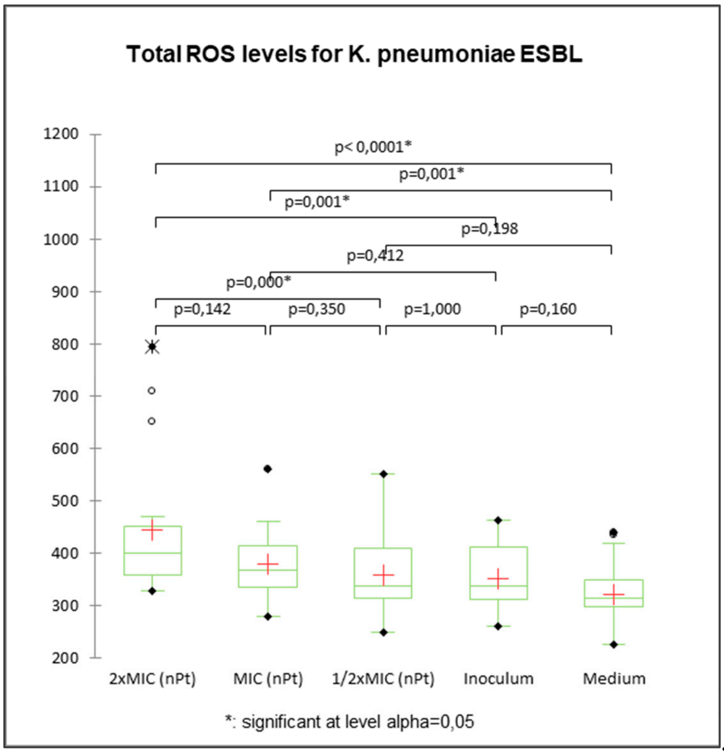

Figure S28. Graphical statistical analysis representation of determined total ROS levels obtained for K. pneumoniae ESBL+ MFBF 10690 exposed to 2 × MIC, MIC, and 1/2 × MIC of nPt as a sum of results of all measurement time points. P values are shown between comparing variables.

Table S28. Statistical significance between comparing variables for the group of results from Figure S28. If $p < 0,05$ the difference is considered as significant (Yes), if the $p > 0,05$ the difference is considered as not significant (No).

Significant differences:

	2xMIC (nPt)	MIC (nPt)	1/2xMIC (nPt)	Inoculum	Medium
2xMIC (nPt)	-	No	**Yes**	**Yes**	**Yes**
MIC (nPt)	No	-	No	No	**Yes**
1/2xMIC (nPt)	**Yes**	No	-	No	No
Inoculum	**Yes**	No	No	-	No
Medium	**Yes**	**Yes**	No	No	-

1.5. *Comparison of total ROS levels between tested strains*

Statistical test used: Kruskal-Wallis test (nonparametric comparison of *n* samples)
Statistical significance value (α) = 0,05

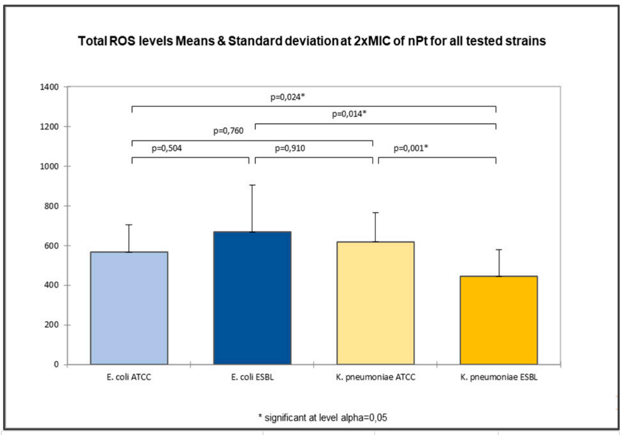

Significant differences:

	E. coli ATCC	E. coli ESBL	K. pneumoniae ATCC	K. pneumoniae ESBL
E. coli ATCC	-	No	No	**Yes**
E. coli ESBL	No	-	No	**Yes**
K. pneumoniae ATCC	No	No	-	**Yes**
K. pneumoniae ESBL	**Yes**	**Yes**	**Yes**	-

(a)

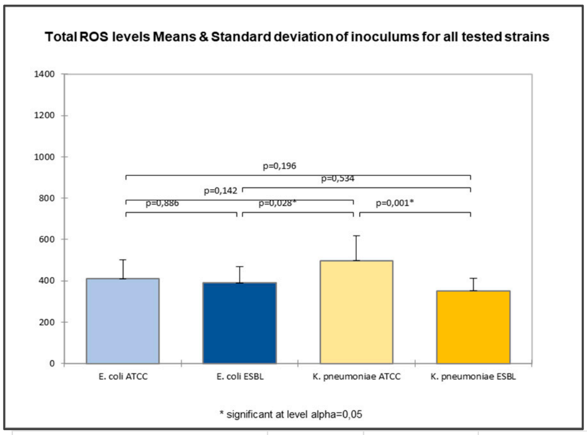

Significant differences:

	E. coli ATCC	E. coli ESBL	K. pneumoniae ATCC	K. pneumoniae ESBL
E. coli ATCC	-	No	No	No
E. coli ESBL	No	-	**Yes**	No
K. pneumoniae ATCC	No	**Yes**	-	**Yes**
K. pneumoniae ESBL	No	No	**Yes**	-

(b)

Figure S29. Comparison of total ROS levels of all measurements for all tested strains with statistical analysis representation: (**a**) for 2 × MIC of nPt exposure, (**b**) for inoculums (untreated bacteria). P values are shown between comparing variables. If p < 0,05 the difference is considered as significant (Yes), if the p > 0,05 the difference is considered as not significant (No).

2. Cell leakage (determination of cellular DNA/RNA and proteins in extracellular fluid sample)

2.1. Extracellular DNA/RNA determination

Statistical test used: Friedman test (nonparametric comparison of *n* samples)
Statistical significance value (α) = 0,05

2.1.1. E. coli ATCC 10536

2.1.1.1. At 0 time point (of exposure to nPt)

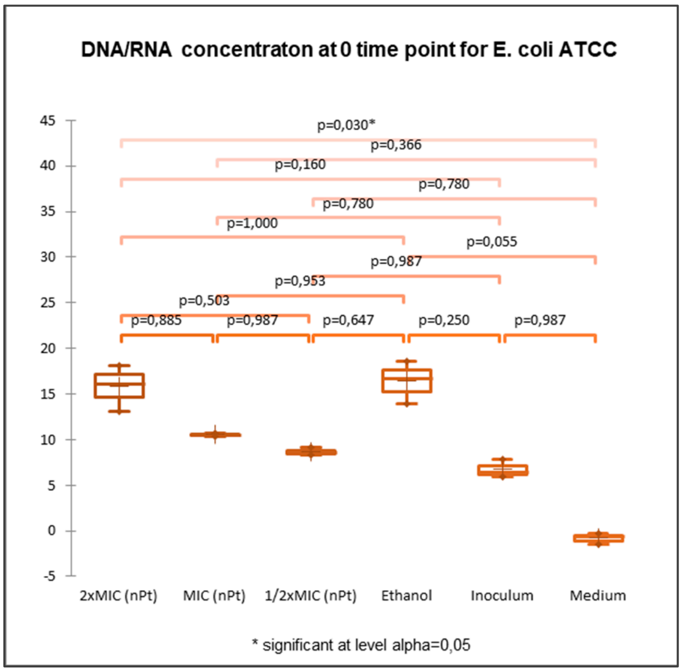

Figure S30. Graphical statistical analysis representation of determined DNA/RNA concentrations obtained for E coli ATCC 10536 exposed to 2 × MIC, MIC, and 1/2 × MIC of nPt at zero time point. P values are shown between comparing variables.

Table S29. Statistical significance between comparing variables for the group of results from Figure S30. If p < 0,05 the difference is considered as significant (Yes), if the p > 0,05 the difference is considered as not significant (No).

Significant differences:						
	2xMIC (nPt)	MIC (nPt)	1/2xMIC (nPt)	Ethanol	Inoculum	Medium
2xMIC (nPt)	-	No	No	No	No	**Yes**
MIC (nPt)	No	-	No	No	No	No
1/2xMIC (nPt)	No	No	-	No	No	No
Ethanol	No	No	No	-	No	No
Inoculum	No	No	No	No	-	No
Medium	**Yes**	No	No	No	No	-

2.1.1.2. After 1 hour (of exposure to nPt)

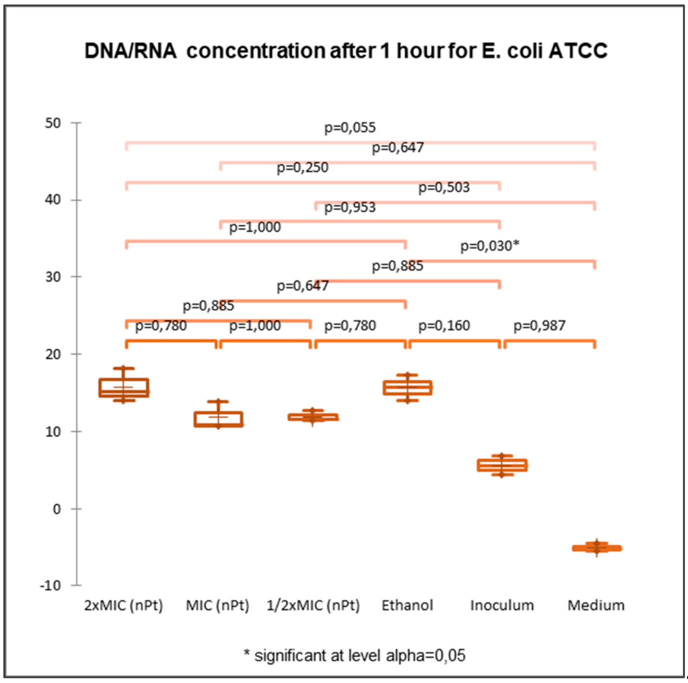

Figure S31. Graphical statistical analysis representation of determined DNA/RNA concentrations obtained for E coli ATCC 10536 exposed to 2 × MIC, MIC, and 1/2 × MIC of nPt after 1 hour of exposure. P values are shown between comparing variables.

Table S30. Statistical significance between comparing variables for the group of results from Figure S31. If $p < 0,05$ the difference is considered as significant (Yes), if the $p > 0,05$ the difference is considered as not significant (No).

Significant differences:

	2xMIC (nPt)	MIC (nPt)	1/2xMIC (nPt)	Ethanol	Inoculum	Medium
2xMIC (nPt)	-	No	No	No	No	No
MIC (nPt)	No	-	No	No	No	No
1/2xMIC (nPt)	No	No	-	No	No	No
Ethanol	No	No	No	-	No	**Yes**
Inoculum	No	No	No	No	-	No
Medium	No	No	No	**Yes**	No	-

2.1.1.3. After 3 hours (of exposure to nPt)

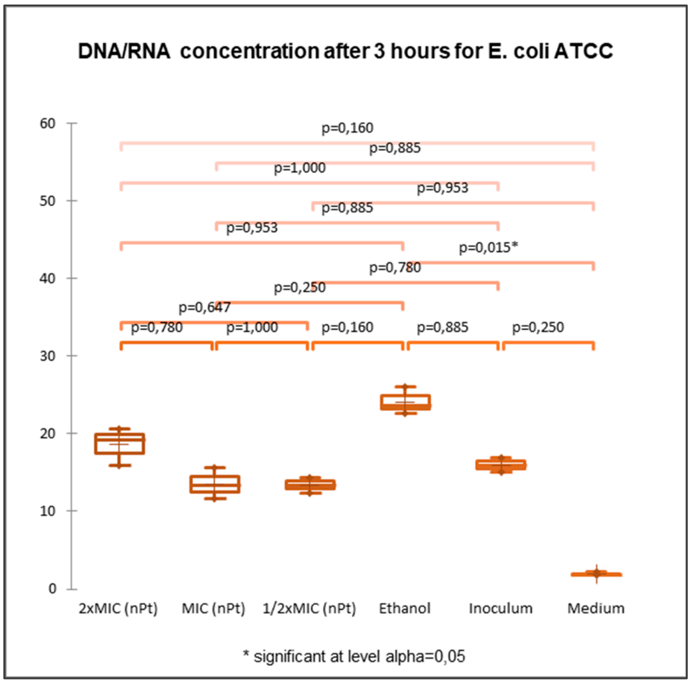

Figure S32. Graphical statistical analysis representation of determined DNA/RNA concentrations obtained for E coli ATCC 10536 exposed to 2 × MIC, MIC, and 1/2 × MIC of nPt after 3 hours of exposure. P values are shown between comparing variables.

Table S31. Statistical significance between comparing variables for the group of results from Figure S32. If p < 0,05 the difference is considered as significant (Yes), if the p > 0,05 the difference is considered as not significant (No).

Significant differences:

	2xMIC (nPt)	MIC (nPt)	1/2xMIC (nPt)	Ethanol	Inoculum	Medium
2xMIC (nPt)	-	No	No	No	No	No
MIC (nPt)	No	-	No	No	No	No
1/2xMIC (nPt)	No	No	-	No	No	No
Ethanol	No	No	No	-	No	**Yes**
Inoculum	No	No	No	No	-	No
Medium	No	No	No	**Yes**	No	-

2.1.1.4. After 6 hours (of exposure to nPt)

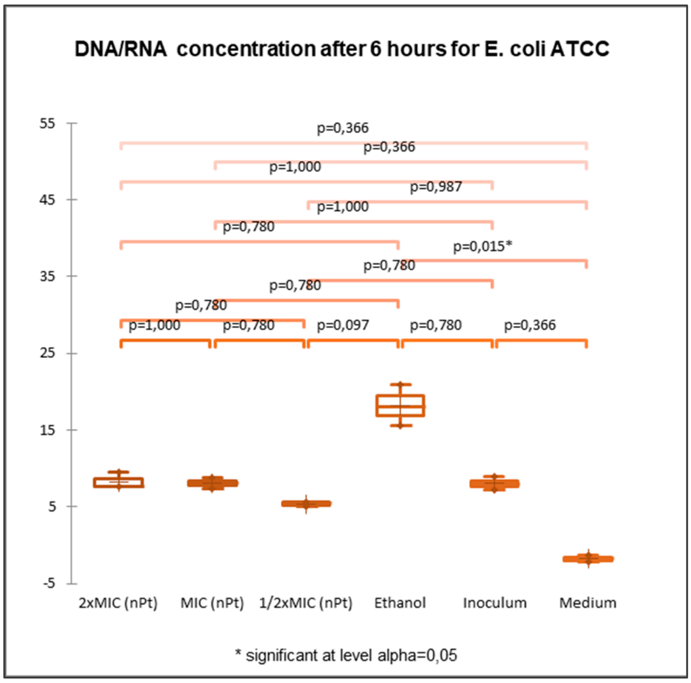

Figure S33. Graphical statistical analysis representation of determined DNA/RNA concentrations obtained for E coli ATCC 10536 exposed to 2 × MIC, MIC, and 1/2 × MIC of nPt after 6 hours of exposure. P values are shown between comparing variables.

Table S32. Statistical significance between comparing variables for the group of results from Figure S33. If p < 0,05 the difference is considered as significant (Yes), if the p > 0,05 the difference is considered as not significant (No).

Significant differences:						
	2xMIC (nPt)	MIC (nPt)	1/2xMIC (nPt)	Ethanol	Inoculum	Medium
2xMIC (nPt)	-	No	No	No	No	No
MIC (nPt)	No	-	No	No	No	No
1/2xMIC (nPt)	No	No	-	No	No	No
Ethanol	No	No	No	-	No	**Yes**
Inoculum	No	No	No	No	-	No
Medium	No	No	No	**Yes**	No	-

2.1.1.5. After 18 hours (of exposure to nPt)

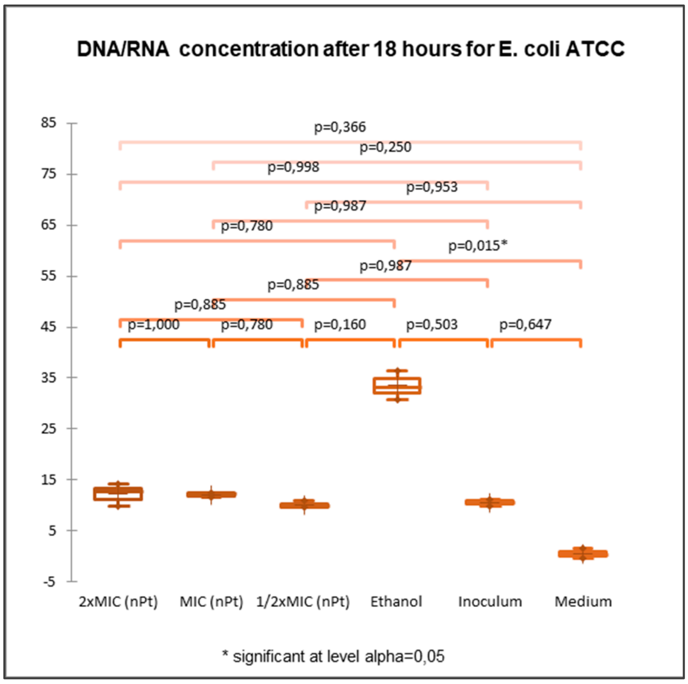

Figure S34. Graphical statistical analysis representation of determined DNA/RNA concentrations obtained for E coli ATCC 10536 exposed to 2 × MIC, MIC, and 1/2 × MIC of nPt after 18 hours of exposure. P values are shown between comparing variables.

Table S33. Statistical significance between comparing variables for the group of results from Figure S34. If p < 0,05 the difference is considered as significant (Yes), if the p > 0,05 the difference is considered as not significant (No).

Significant differences:						
	2xMIC (nPt)	MIC (nPt)	1/2xMIC (nPt)	Ethanol	Inoculum	Medium
2xMIC (nPt)	-	No	No	No	No	No
MIC (nPt)	No	-	No	No	No	No
1/2xMIC (nPt)	No	No	-	No	No	No
Ethanol	No	No	No	-	No	**Yes**
Inoculum	No	No	No	No	-	No
Medium	No	No	No	**Yes**	No	-

2.1.1.6. After 24 hours (of exposure to nPt).

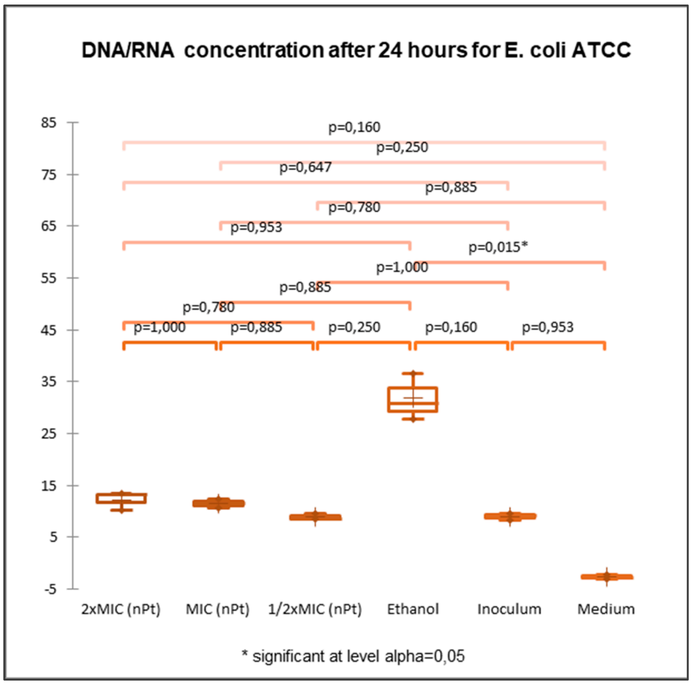

Figure S35. Graphical statistical analysis representation of determined DNA/RNA concentrations obtained for E coli ATCC 10536 exposed to 2 × MIC, MIC, and 1/2 × MIC of nPt after 24 hours of exposure. P values are shown between comparing variables.

Table S34. Statistical significance between comparing variables for the group of results from Figure S35. If p < 0,05 the difference is considered as significant (Yes), if the p > 0,05 the difference is considered as not significant (No).

Significant differences:						
	2xMIC (nPt)	MIC (nPt)	1/2xMIC (nPt)	Ethanol	Inoculum	Medium
2xMIC (nPt)	-	No	No	No	No	No
MIC (nPt)	No	-	No	No	No	No
1/2xMIC (nPt)	No	No	-	No	No	No
Ethanol	No	No	No	-	No	**Yes**
Inoculum	No	No	No	No	-	No
Medium	No	No	No	**Yes**	No	-

2.1.1.7. Total DNA/RNA concentration statistical count (sum of results of all measurement time points)

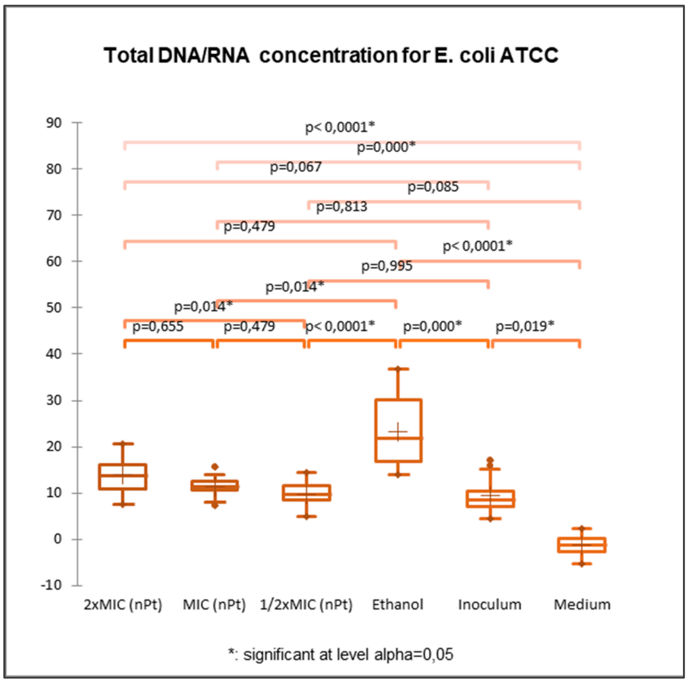

Figure S36. Graphical statistical analysis representation of determined total DNA/RNA concentrations obtained for E coli ATCC 10536 exposed to 2 × MIC, MIC, and 1/2 × MIC of nPt as a sum of results of all measurement time points. P values are shown between comparing variables.

Table S35. Statistical significance between comparing variables for the group of results from Figure S36. If p < 0,05 the difference is considered as significant (Yes), if the p > 0,05 the difference is considered as not significant (No).

Significant differences:						
	2xMIC (nPt)	MIC (nPt)	1/2xMIC (nPt)	Ethanol	Inoculum	Medium
2xMIC (nPt)	-	No	**Yes**	No	No	**Yes**
MIC (nPt)	No	-	No	**Yes**	No	**Yes**
1/2xMIC (nPt)	**Yes**	No	-	**Yes**	No	No
Ethanol	No	**Yes**	**Yes**	-	**Yes**	**Yes**
Inoculum	No	No	No	**Yes**	-	**Yes**
Medium	**Yes**	**Yes**	No	**Yes**	**Yes**	-

2.1.2. E. coli ESBL+ MFBF 12795

2.1.2.1. At 0 time point (of exposure to nPt)

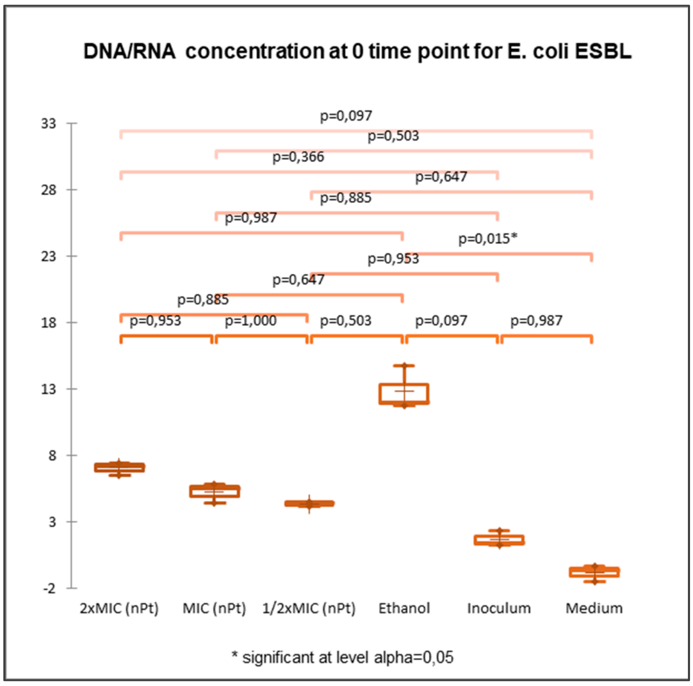

Figure S37. Graphical statistical analysis representation of determined DNA/RNA concentrations obtained for E coli ESBL+ MFBF 12795 exposed to 2 × MIC, MIC, and 1/2 × MIC of nPt at zero time point. P values are shown between comparing variables.

Table S36. Statistical significance between comparing variables for the group of results from Figure S37. If p < 0,05 the difference is considered as significant (Yes), if the p > 0,05 the difference is considered as not significant (No).

Significant differences:						
	2xMIC (nPt)	MIC (nPt)	1/2xMIC (nPt)	Ethanol	Inoculum	Medium
2xMIC (nPt)	-	No	No	No	No	No
MIC (nPt)	No	-	No	No	No	No
1/2xMIC (nPt)	No	No	-	No	No	No
Ethanol	No	No	No	-	No	**Yes**
Inoculum	No	No	No	No	-	No
Medium	No	No	No	**Yes**	No	-

2.1.2.2. After 1 hour (of exposure to nPt)

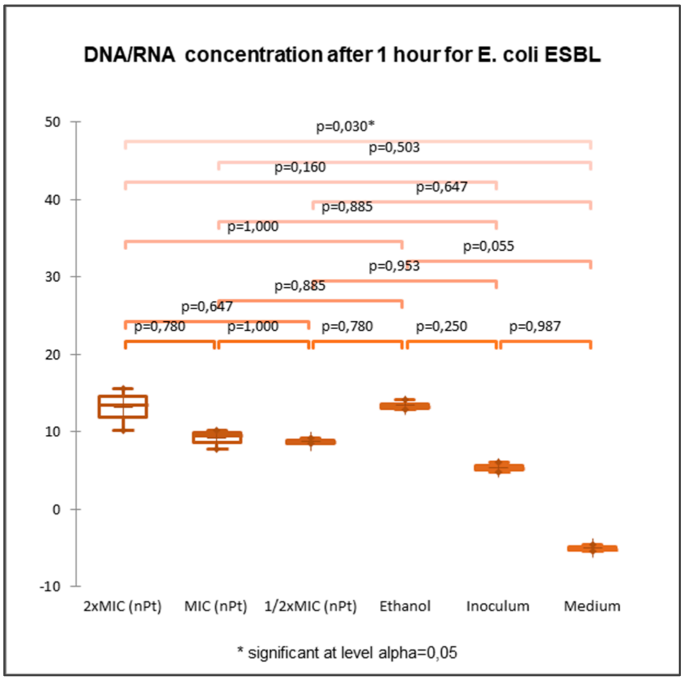

Figure S38. Graphical statistical analysis representation of determined DNA/RNA concentrations obtained for E coli ESBL+ MFBF 12795 exposed to 2 × MIC, MIC, and 1/2 × MIC of nPt after 1 hour of exposure. P values are shown between comparing variables.

Table S37. Statistical significance between comparing variables for the group of results from Figure S38. If p < 0,05 the difference is considered as significant (Yes), if the p > 0,05 the difference is considered as not significant (No).

Significant differences:

	2xMIC (nPt)	MIC (nPt)	1/2xMIC (nPt)	Ethanol	Inoculum	Medium
2xMIC (nPt)	-	No	No	No	No	**Yes**
MIC (nPt)	No	-	No	No	No	No
1/2xMIC (nPt)	No	No	-	No	No	No
Ethanol	No	No	No	-	No	No
Inoculum	No	No	No	No	-	No
Medium	**Yes**	No	No	No	No	-

2.1.2.3. After 3 hours (of exposure to nPt)

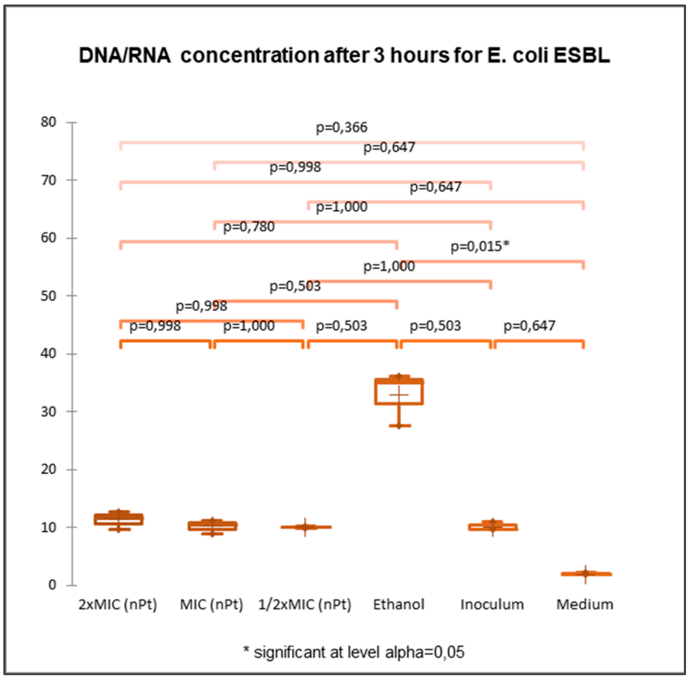

Figure S39. Graphical statistical analysis representation of determined DNA/RNA concentrations obtained for E coli ESBL+ MFBF 12795 exposed to 2 × MIC, MIC, and 1/2 × MIC of nPt after 3 hours of exposure. P values are shown between comparing variables.

Table S38. Statistical significance between comparing variables for the group of results from Figure S39. If p < 0,05 the difference is considered as significant (Yes), if the p > 0,05 the difference is considered as not significant (No).

Significant differences:

	2xMIC (nPt)	MIC (nPt)	1/2xMIC (nPt)	Ethanol	Inoculum	Medium
2xMIC (nPt)	-	No	No	No	No	No
MIC (nPt)	No	-	No	No	No	No
1/2xMIC (nPt)	No	No	-	No	No	No
Ethanol	No	No	No	-	No	**Yes**
Inoculum	No	No	No	No	-	No
Medium	No	No	No	**Yes**	No	-

2.1.2.4. After 6 hours (of exposure to nPt)

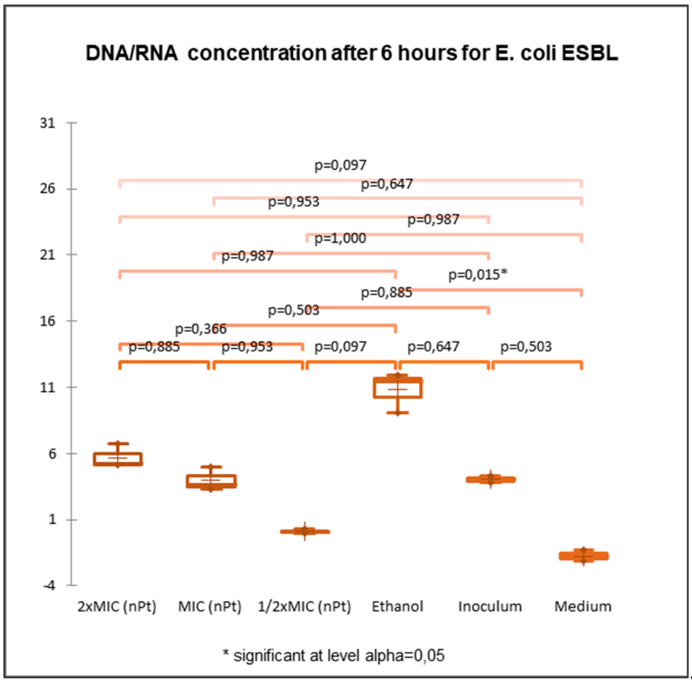

Figure S40. Graphical statistical analysis representation of determined DNA/RNA concentrations obtained for E coli ESBL+ MFBF 12795 exposed to 2 × MIC, MIC, and 1/2 × MIC of nPt after 6 hours of exposure. P values are shown between comparing variables.

Table S39. Statistical significance between comparing variables for the group of results from Figure S40. If p < 0,05 the difference is considered as significant (Yes), if the p > 0,05 the difference is considered as not significant (No).

Significant differences:						
	2xMIC (nPt)	MIC (nPt)	1/2xMIC (nPt)	Ethanol	Inoculum	Medium
2xMIC (nPt)	-	No	No	No	No	No
MIC (nPt)	No	-	No	No	No	No
1/2xMIC (nPt)	No	No	-	No	No	No
Ethanol	No	No	No	-	No	**Yes**
Inoculum	No	No	No	No	-	No
Medium	No	No	No	**Yes**	No	-

2.1.2.5. After 18 hours (of exposure to nPt)

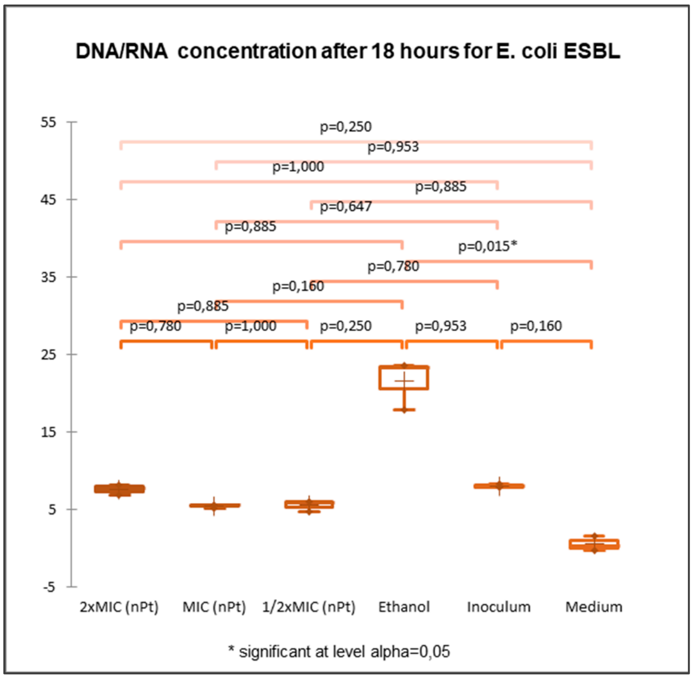

Figure S41. Graphical statistical analysis representation of determined DNA/RNA concentrations obtained for E coli ESBL+ MFBF 12795 exposed to 2 × MIC, MIC, and 1/2 × MIC of nPt after 18 hours of exposure. P values are shown between comparing variables.

Table S40. Statistical significance between comparing variables for the group of results from Figure S41. If $p < 0,05$ the difference is considered as significant (Yes), if the $p > 0,05$ the difference is considered as not significant (No).

Significant differences:						
	2xMIC (nPt)	MIC (nPt)	1/2xMIC (nPt)	Ethanol	Inoculum	Medium
2xMIC (nPt)	-	No	No	No	No	No
MIC (nPt)	No	-	No	No	No	No
1/2xMIC (nPt)	No	No	-	No	No	No
Ethanol	No	No	No	-	No	**Yes**
Inoculum	No	No	No	No	-	No
Medium	No	No	No	**Yes**	No	-

2.1.2.6. After 24 hours (of exposure to nPt)

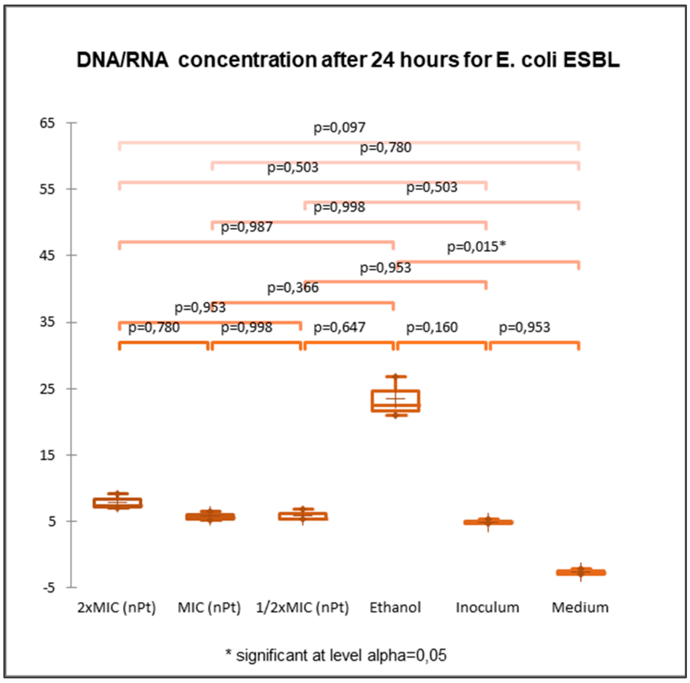

Figure S42. Graphical statistical analysis representation of determined DNA/RNA concentrations obtained for E coli ESBL+ MFBF 12795 exposed to 2 × MIC, MIC, and 1/2 × MIC of nPt after 24 hours of exposure. P values are shown between comparing variables.

Table S41. Statistical significance between comparing variables for the group of results from Figure S42. If p < 0,05 the difference is considered as significant (Yes), if the p > 0,05 the difference is considered as not significant (No).

Significant differences:						
	2xMIC (nPt)	MIC (nPt)	1/2xMIC (nPt)	Ethanol	Inoculum	Medium
2xMIC (nPt)	-	No	No	No	No	No
MIC (nPt)	No	-	No	No	No	No
1/2xMIC (nPt)	No	No	-	No	No	No
Ethanol	No	No	No	-	No	**Yes**
Inoculum	No	No	No	No	-	No
Medium	No	No	No	**Yes**	No	-

2.1.2.7. Total DNA/RNA concentration statistical count (sum of results of all measurement time points)

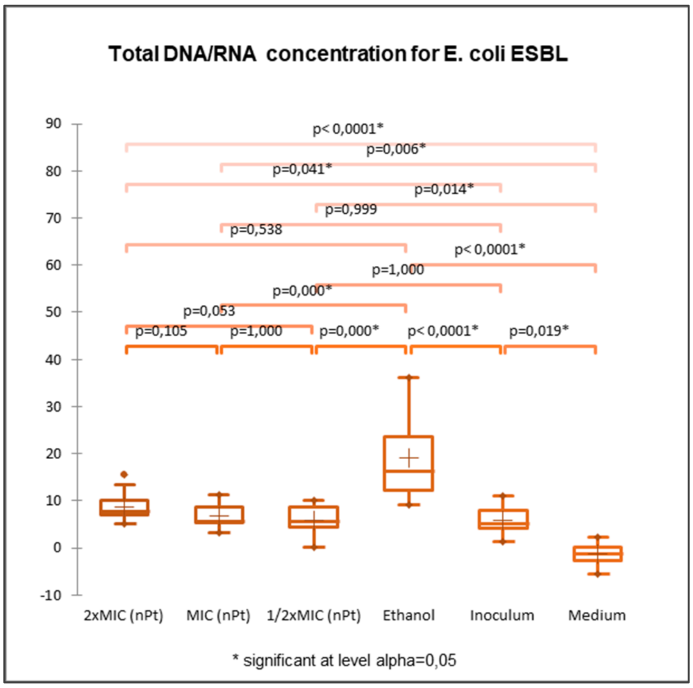

Figure S43. Graphical statistical analysis representation of determined total DNA/RNA concentrations obtained for E coli ESBL+ MFBF 12795 exposed to 2 × MIC, MIC, and 1/2 × MIC of nPt as a sum of results of all measurement time points. P values are shown between comparing variables.

Table S42. Statistical significance between comparing variables for the group of results from Figure S43. If p < 0,05 the difference is considered as significant (Yes), if the p > 0,05 the difference is considered as not significant (No).

Significant differences:						
	2xMIC (nPt)	MIC (nPt)	1/2xMIC (nPt)	Ethanol	Inoculum	Medium
2xMIC (nPt)	-	No	No	No	**Yes**	**Yes**
MIC (nPt)	No	-	No	**Yes**	No	**Yes**
1/2xMIC (nPt)	No	No	-	**Yes**	No	**Yes**
Ethanol	No	**Yes**	**Yes**	-	**Yes**	**Yes**
Inoculum	**Yes**	No	No	**Yes**	-	**Yes**
Medium	**Yes**	**Yes**	**Yes**	**Yes**	**Yes**	-

2.1.3. K. pneumoniae ATCC 700603

2.1.3.1. At 0 time point (of exposure to nPt)

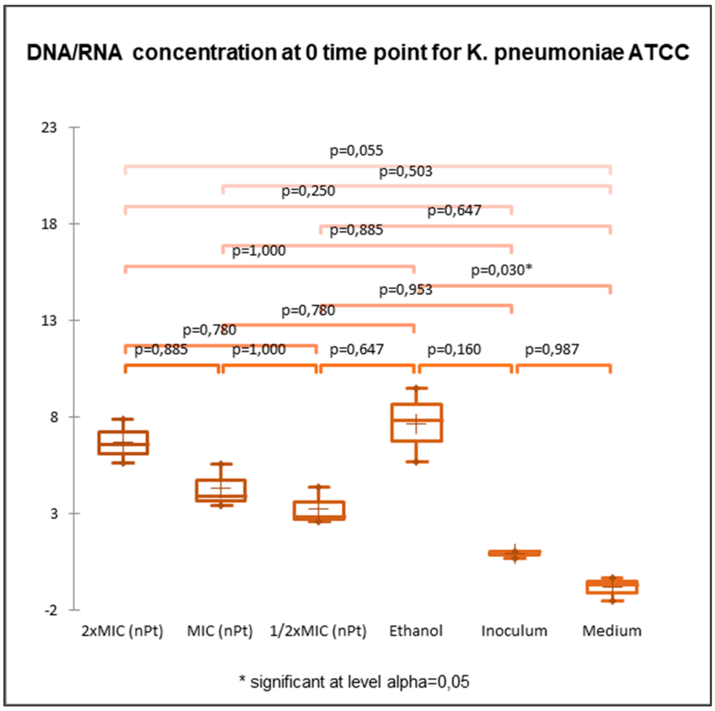

Figure S44. Graphical statistical analysis representation of determined DNA/RNA concentrations obtained for K. pneumoniae ATCC 700603 exposed to 2 × MIC, MIC, and 1/2 × MIC of nPt at zero time point. P values are shown between comparing variables.

Table S43. Statistical significance between comparing variables for the group of results from Figure S44. If p < 0,05 the difference is considered as significant (Yes), if the p > 0,05 the difference is considered as not significant (No).

Significant differences:						
	2xMIC (nPt)	MIC (nPt)	1/2xMIC (nPt)	Ethanol	Inoculum	Medium
2xMIC (nPt)	-	No	No	No	No	No
MIC (nPt)	No	-	No	No	No	No
1/2xMIC (nPt)	No	No	-	No	No	No
Ethanol	No	No	No	-	No	**Yes**
Inoculum	No	No	No	No	-	No
Medium	No	No	No	**Yes**	No	-

2.1.3.2. After 1 hour (of exposure to nPt)

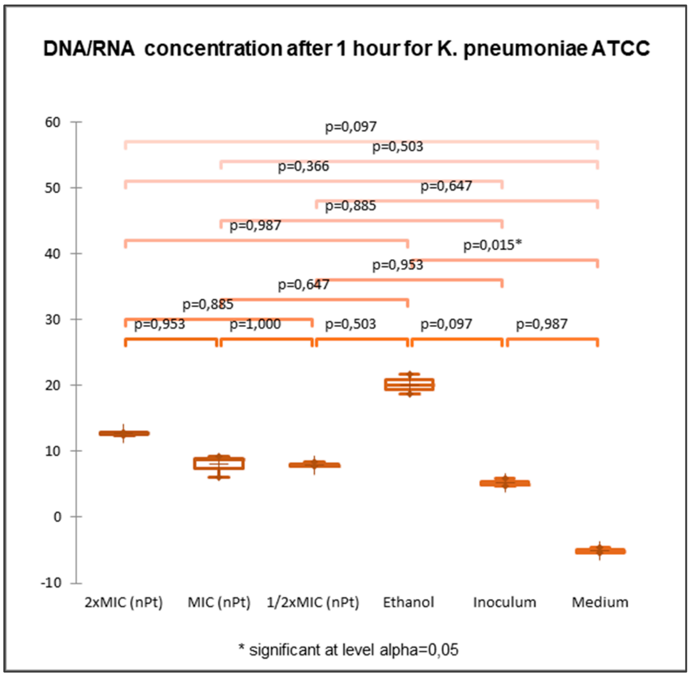

Figure S45. Graphical statistical analysis representation of determined DNA/RNA concentrations obtained for K. pneumoniae ATCC 700603 exposed to 2 × MIC, MIC, and 1/2 × MIC of nPt after 1 hour of exposure. P values are shown between comparing variables.

Table S44. Statistical significance between comparing variables for the group of results from Figure S45. If p < 0,05 the difference is considered as significant (Yes), if the p > 0,05 the difference is considered as not significant (No).

Significant differences:						
	2xMIC (nPt)	MIC (nPt)	1/2xMIC (nPt)	Ethanol	Inoculum	Medium
2xMIC (nPt)	-	No	No	No	No	No
MIC (nPt)	No	-	No	No	No	No
1/2xMIC (nPt)	No	No	-	No	No	No
Ethanol	No	No	No	-	No	**Yes**
Inoculum	No	No	No	No	-	No
Medium	No	No	No	**Yes**	No	-

2.1.3.3. After 3 hours (of exposure to nPt)

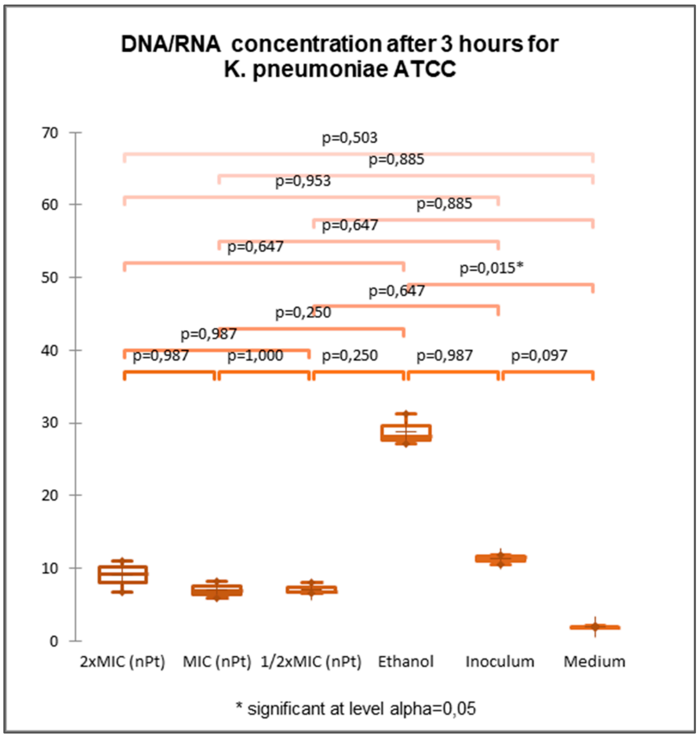

Figure S46. Graphical statistical analysis representation of determined DNA/RNA concentrations obtained for K. pneumoniae ATCC 700603 exposed to 2 × MIC, MIC, and 1/2 × MIC of nPt after 3 hours of exposure. P values are shown between comparing variables.

Table S45. Statistical significance between comparing variables for the group of results from Figure S46. If p < 0,05 the difference is considered as significant (Yes), if the p > 0,05 the difference is considered as not significant (No).

Significant differences:

	2xMIC (nPt)	MIC (nPt)	1/2xMIC (nPt)	Ethanol	Inoculum	Medium
2xMIC (nPt)	-	No	No	No	No	No
MIC (nPt)	No	-	No	No	No	No
1/2xMIC (nPt)	No	No	-	No	No	No
Ethanol	No	No	No	-	No	**Yes**
Inoculum	No	No	No	No	-	No
Medium	No	No	No	**Yes**	No	-

2.1.3.4. After 6 hours (of exposure to nPt)

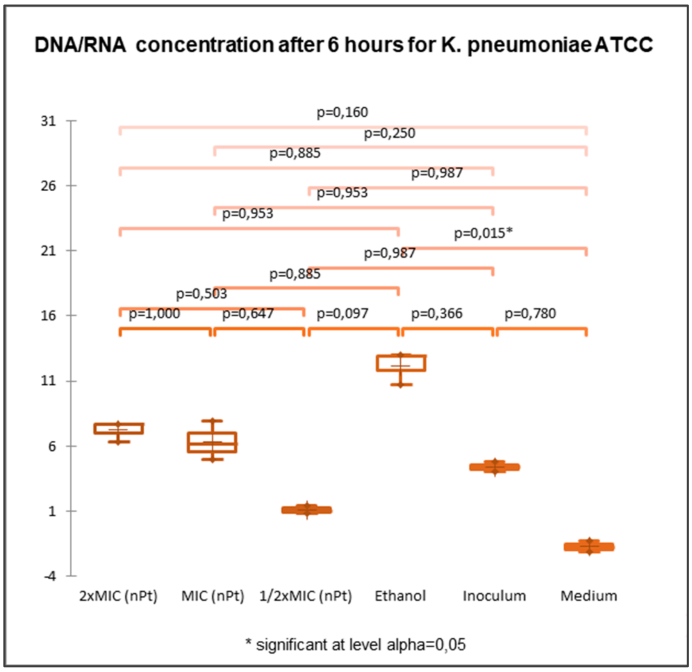

Figure S47. Graphical statistical analysis representation of determined DNA/RNA concentrations obtained for K. pneumoniae ATCC 700603 exposed to 2 × MIC, MIC, and 1/2 × MIC of nPt after 6 hours of exposure. P values are shown between comparing variables.

Table S46. Statistical significance between comparing variables for the group of results from Figure S47. If p < 0,05 the difference is considered as significant (Yes), if the p > 0,05 the difference is considered as not significant (No).

Significant differences:						
	2xMIC (nPt)	MIC (nPt)	1/2xMIC (nPt)	Ethanol	Inoculum	Medium
2xMIC (nPt)	-	No	No	No	No	No
MIC (nPt)	No	-	No	No	No	No
1/2xMIC (nPt)	No	No	-	No	No	No
Ethanol	No	No	No	-	No	**Yes**
Inoculum	No	No	No	No	-	No
Medium	No	No	No	**Yes**	No	-

2.1.3.5. After 18 hours (of exposure to nPt)

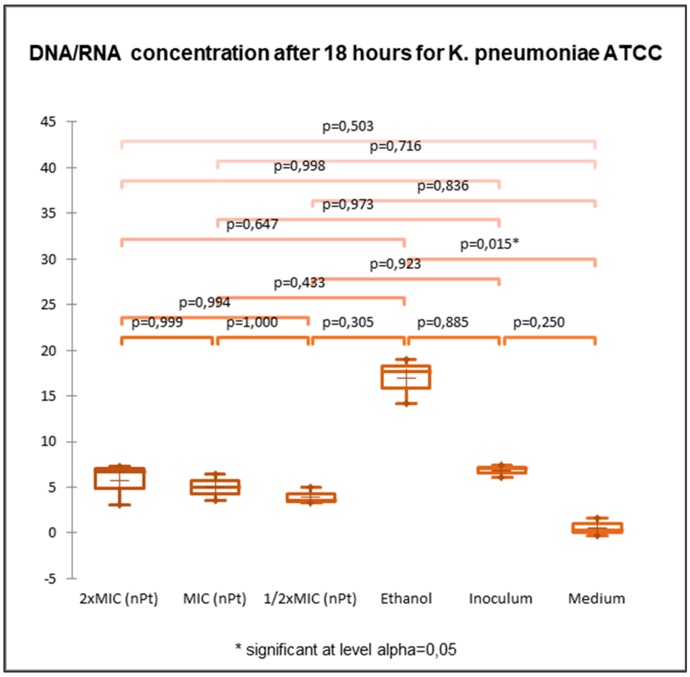

Figure S48. Graphical statistical analysis representation of determined DNA/RNA concentrations obtained for K. pneumoniae ATCC 700603 exposed to 2 × MIC, MIC, and 1/2 × MIC of nPt after 18 hours of exposure. P values are shown between comparing variables.

Table S47. Statistical significance between comparing variables for the group of results from Figure S48. If p < 0,05 the difference is considered as significant (Yes), if the p > 0,05 the difference is considered as not significant (No).

Significant differences:						
	2xMIC (nPt)	MIC (nPt)	1/2xMIC (nPt)	Ethanol	Inoculum	Medium
2xMIC (nPt)	-	No	No	No	No	No
MIC (nPt)	No	-	No	No	No	No
1/2xMIC (nPt)	No	No	-	No	No	No
Ethanol	No	No	No	-	No	**Yes**
Inoculum	No	No	No	No	-	No
Medium	No	No	No	**Yes**	No	-

2.1.3.6. After 24 hours (of exposure to nPt)

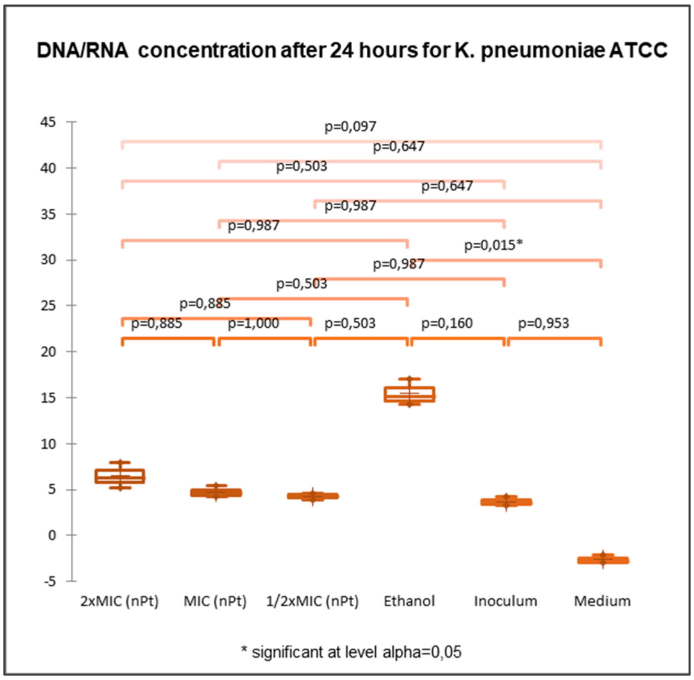

Figure S49. Graphical statistical analysis representation of determined DNA/RNA concentrations obtained for K. pneumoniae ATCC 700603 exposed to 2 × MIC, MIC, and 1/2 × MIC of nPt after 24 hours of exposure. P values are shown between comparing variables.

Table S48. Statistical significance between comparing variables for the group of results from Figure S49. If p < 0,05 the difference is considered as significant (Yes), if the p > 0,05 the difference is considered as not significant (No).

Significant differences:						
	2xMIC (nPt)	MIC (nPt)	1/2xMIC (nPt)	Ethanol	Inoculum	Medium
2xMIC (nPt)	-	No	No	No	No	No
MIC (nPt)	No	-	No	No	No	No
1/2xMIC (nPt)	No	No	-	No	No	No
Ethanol	No	No	No	-	No	**Yes**
Inoculum	No	No	No	No	-	No
Medium	No	No	No	**Yes**	No	-

2.1.3.7. Total DNA/RNA concentration statistical count (sum of results of all measurement time points)

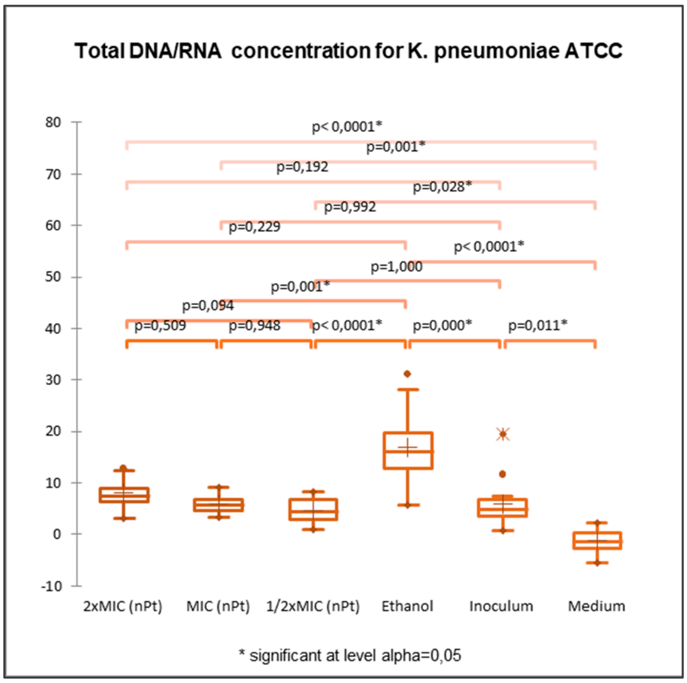

Figure S50. Graphical statistical analysis representation of determined total DNA/RNA concentrations obtained for K. pneumoniae ATCC 700603 exposed to 2 × MIC, MIC, and 1/2 × MIC of nPt as a sum of results of all measurement time points. P values are shown between comparing variables.

Table S49. Statistical significance between comparing variables for the group of results from Figure S50. If p < 0,05 the difference is considered as significant (Yes), if the p > 0,05 the difference is considered as not significant (No).

Significant differences:

	2xMIC (nPt)	MIC (nPt)	1/2xMIC (nPt)	Ethanol	Inoculum	Medium
2xMIC (nPt)	-	No	No	No	No	**Yes**
MIC (nPt)	No	-	No	**Yes**	No	**Yes**
1/2xMIC (nPt)	No	No	-	**Yes**	No	**Yes**
Ethanol	No	**Yes**	**Yes**	-	**Yes**	**Yes**
Inoculum	No	No	No	**Yes**	-	**Yes**
Medium	**Yes**	**Yes**	**Yes**	**Yes**	**Yes**	-

2.1.4. K. pneumoniae ESBL+ MFBF 10690

2.1.4.1. At 0 time point (of exposure to nPt)

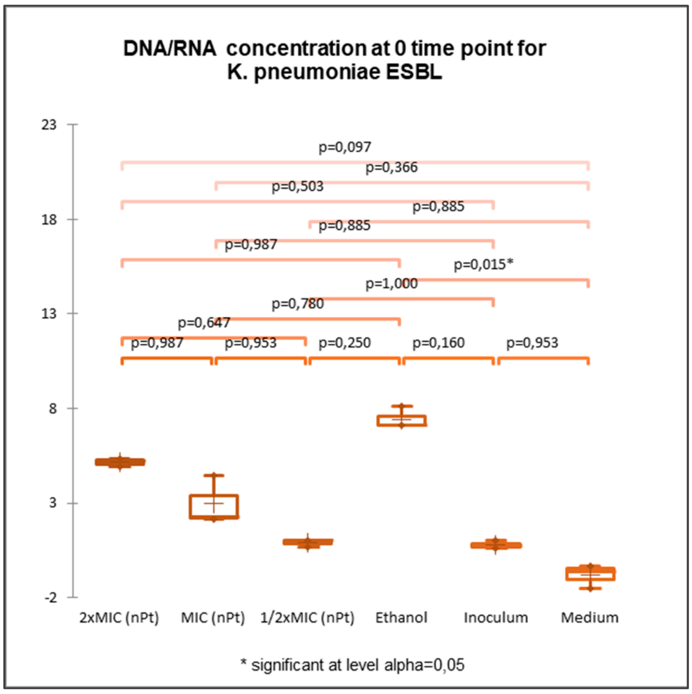

Figure S51. Graphical statistical analysis representation of determined total DNA/RNA concentrations obtained for K. pneumoniae ESBL+ MFBF 10690 exposed to 2 × MIC, MIC, and 1/2 × MIC at zero time point. P values are shown between comparing variables.

Table S50. Statistical significance between comparing variables for the group of results from Figure S51. If p < 0,05 the difference is considered as significant (Yes), if the p > 0,05 the difference is considered as not significant (No).

Significant differences:						
	2xMIC (nPt)	MIC (nPt)	1/2xMIC (nPt)	Ethanol	Inoculum	Medium
2xMIC (nPt)	-	No	No	No	No	No
MIC (nPt)	No	-	No	No	No	No
1/2xMIC (nPt)	No	No	-	No	No	No
Ethanol	No	No	No	-	No	**Yes**
Inoculum	No	No	No	No	-	No
Medium	No	No	No	**Yes**	No	-

2.1.4.2. After 1 hour (of exposure to nPt)

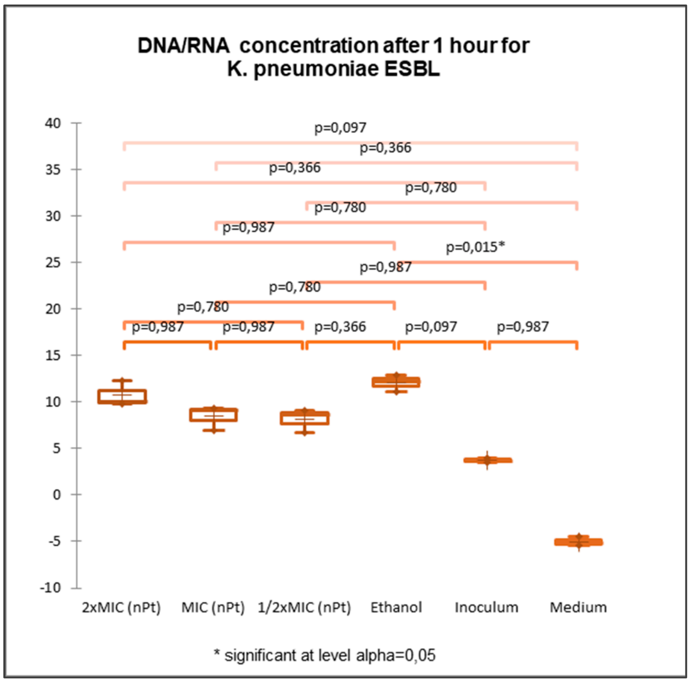

Figure S52. Graphical statistical analysis representation of determined total DNA/RNA concentrations obtained for K. pneumoniae ESBL+ MFBF 10690 exposed to 2 × MIC, MIC, and 1/2 × MIC after 1 hour of exposure. P values are shown between comparing variables.

Table S51. Statistical significance between comparing variables for the group of results from Figure S52. If p < 0,05 the difference is considered as significant (Yes), if the p > 0,05 the difference is considered as not significant (No).

Significant differences:						
	2xMIC (nPt)	MIC (nPt)	1/2xMIC (nPt)	Ethanol	Inoculum	Medium
2xMIC (nPt)	-	No	No	No	No	No
MIC (nPt)	No	-	No	No	No	No
1/2xMIC (nPt)	No	No	-	No	No	No
Ethanol	No	No	No	-	No	**Yes**
Inoculum	No	No	No	No	-	No
Medium	No	No	No	**Yes**	No	-

2.1.4.3. After 3 hours (of exposure to nPt)

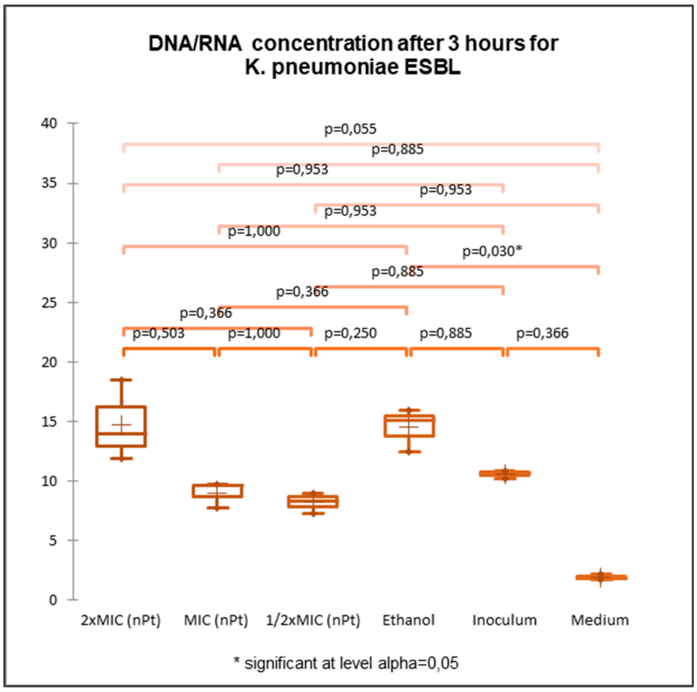

Figure S53. Graphical statistical analysis representation of determined total DNA/RNA concentrations obtained for K. pneumoniae ESBL+ MFBF 10690 exposed to 2 × MIC, MIC, and 1/2 × MIC after 3 hours of exposure. P values are shown between comparing variables.

Table S52. Statistical significance between comparing variables for the group of results from Figure S53. If p < 0,05 the difference is considered as significant (Yes), if the p > 0,05 the difference is considered as not significant (No).

Significant differences:						
	2xMIC (nPt)	MIC (nPt)	1/2xMIC (nPt)	Ethanol	Inoculum	Medium
2xMIC (nPt)	-	No	No	No	No	No
MIC (nPt)	No	-	No	No	No	No
1/2xMIC (nPt)	No	No	-	No	No	No
Ethanol	No	No	No	-	No	**Yes**
Inoculum	No	No	No	No	-	No
Medium	No	No	No	**Yes**	No	-

2.1.4.4. After 6 hours (of exposure to nPt)

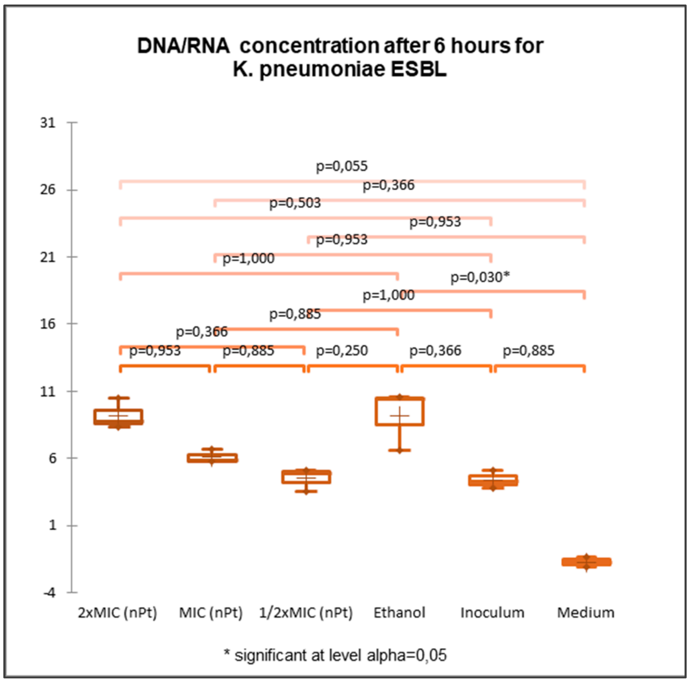

Figure S54. Graphical statistical analysis representation of determined total DNA/RNA concentrations obtained for K. pneumoniae ESBL+ MFBF 10690 exposed to 2 × MIC, MIC, and 1/2 × MIC after 6 hours of exposure. P values are shown between comparing variables.

Table S53. Statistical significance between comparing variables for the group of results from Figure S54. If p < 0,05 the difference is considered as significant (Yes), if the p > 0,05 the difference is considered as not significant (No).

Significant differences:

	2xMIC (nPt)	MIC (nPt)	1/2xMIC (nPt)	Ethanol	Inoculum	Medium
2xMIC (nPt)	-	No	No	No	No	No
MIC (nPt)	No	-	No	No	No	No
1/2xMIC (nPt)	No	No	-	No	No	No
Ethanol	No	No	No	-	No	**Yes**
Inoculum	No	No	No	No	-	No
Medium	No	No	No	**Yes**	No	-

2.1.4.5. After 18 hours (of exposure to nPt)

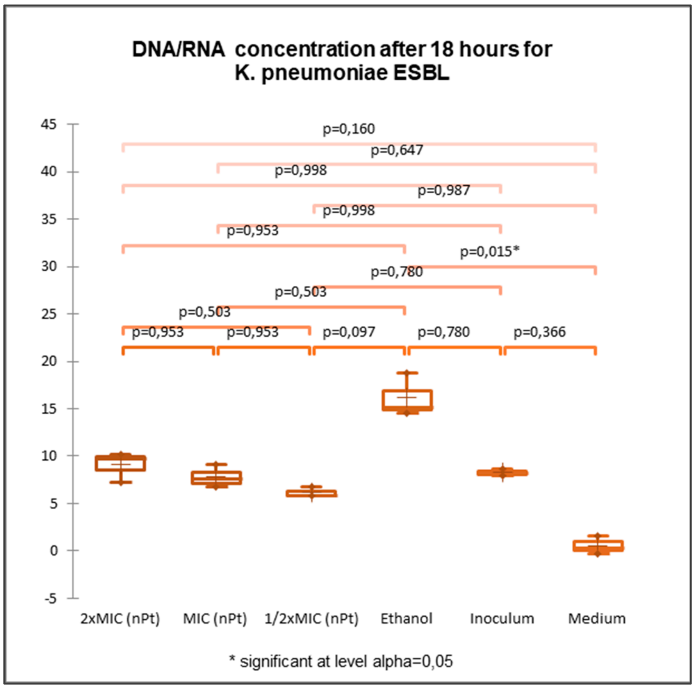

Figure S55. Graphical statistical analysis representation of determined total DNA/RNA concentrations obtained for K. pneumoniae ESBL+ MFBF 10690 exposed to 2 × MIC, MIC, and 1/2 × MIC after 18 hours of exposure. P values are shown between comparing variables.

Table S54. Statistical significance between comparing variables for the group of results from Figure S55. If p < 0,05 the difference is considered as significant (Yes), if the p > 0,05 the difference is considered as not significant (No).

Significant differences:						
	2xMIC (nPt)	MIC (nPt)	1/2xMIC (nPt)	Ethanol	Inoculum	Medium
2xMIC (nPt)	-	No	No	No	No	No
MIC (nPt)	No	-	No	No	No	No
1/2xMIC (nPt)	No	No	-	No	No	No
Ethanol	No	No	No	-	No	**Yes**
Inoculum	No	No	No	No	-	No
Medium	No	No	No	**Yes**	No	-

2.1.4.6. After 24 hours (of exposure to nPt)

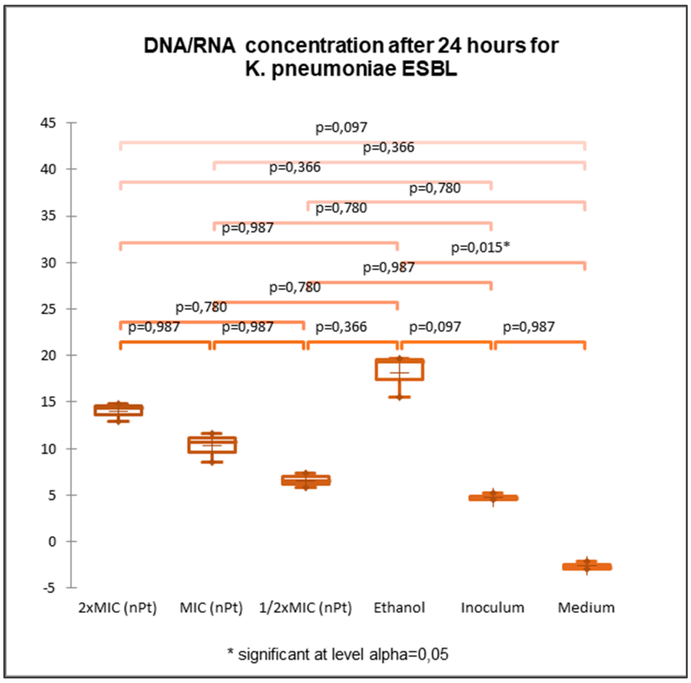

Figure S56. Graphical statistical analysis representation of determined total DNA/RNA concentrations obtained for K. pneumoniae ESBL+ MFBF 10690 exposed to 2 × MIC, MIC, and 1/2 × MIC after 24 hours of exposure. P values are shown between comparing variables.

Table S55. Statistical significance between comparing variables for the group of results from Figure S56. If p < 0,05 the difference is considered as significant (Yes), if the p > 0,05 the difference is considered as not significant (No).

Significant differences:						
	2xMIC (nPt)	MIC (nPt)	1/2xMIC (nPt)	Ethanol	Inoculum	Medium
2xMIC (nPt)	-	No	No	No	No	No
MIC (nPt)	No	-	No	No	No	No
1/2xMIC (nPt)	No	No	-	No	No	No
Ethanol	No	No	No	-	No	**Yes**
Inoculum	No	No	No	No	-	No
Medium	No	No	No	**Yes**	No	-

2.1.4.7. Total DNA/RNA concentration statistical count (sum of results of all measurement time points)

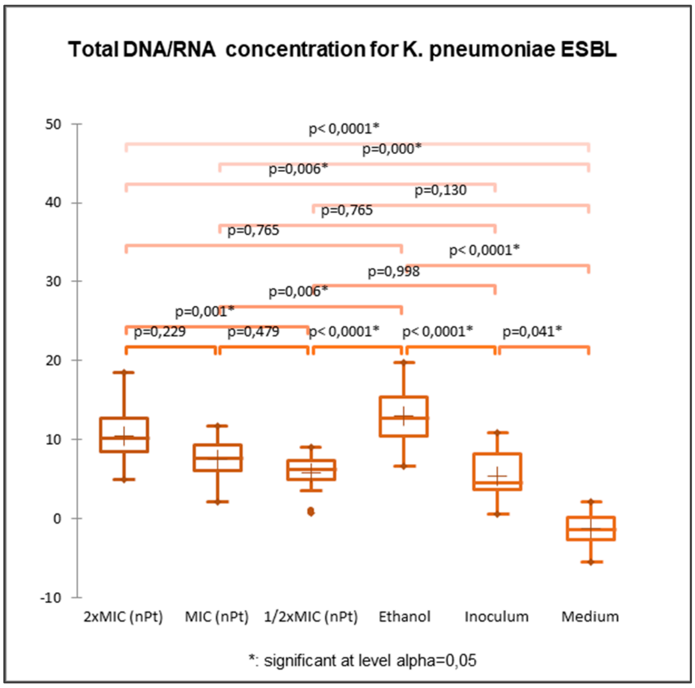

Figure S57. Graphical statistical analysis representation of determined total DNA/RNA concentrations obtained for K. pneumoniae ESBL+ MFBF 10690 exposed to 2 × MIC, MIC, and 1/2 × MIC as a sum of results of all measurement time points. P values are shown between comparing variables.

Table S56. Statistical significance between comparing variables for the group of results from Figure S57. If p < 0,05 the difference is considered as significant (Yes), if the p > 0,05 the difference is considered as not significant (No).

Significant differences:						
	2xMIC (nPt)	MIC (nPt)	1/2xMIC (nPt)	Ethanol	Inoculum	Medium
2xMIC (nPt)	-	No	**Yes**	No	**Yes**	**Yes**
MIC (nPt)	No	-	No	**Yes**	No	**Yes**
1/2xMIC (nPt)	**Yes**	No	-	**Yes**	No	No
Ethanol	No	**Yes**	**Yes**	-	**Yes**	**Yes**
Inoculum	**Yes**	No	No	**Yes**	-	**Yes**
Medium	**Yes**	**Yes**	No	**Yes**	**Yes**	-

2.1.5. Comparison of total DNA/RNA concentration between tested strains

Statistical test used: Kruskal-Wallis test (nonparametric comparison of n samples)

Statistical significance value (α) = 0,05

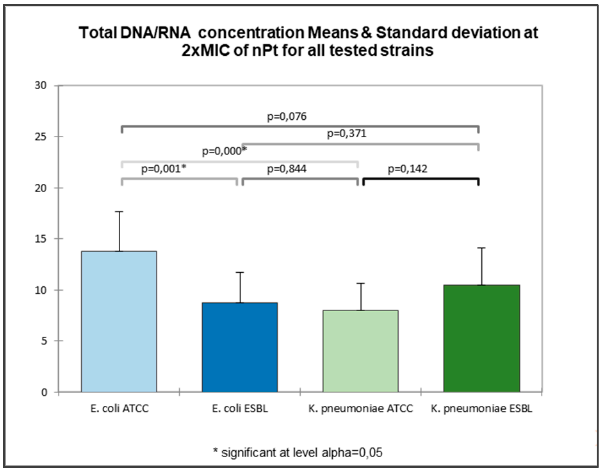

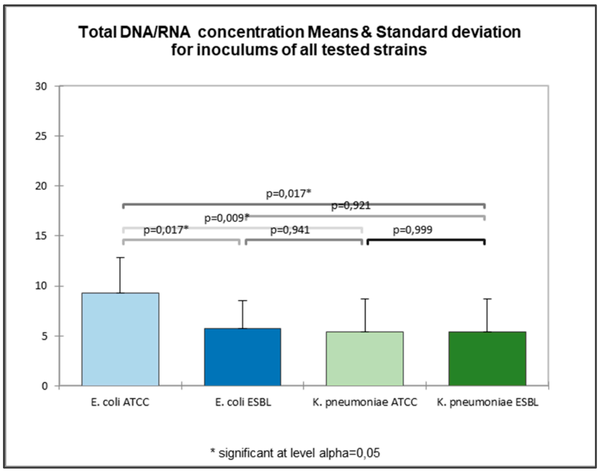

Significant differences:

	E. coli ATCC	E. coli ESBL	K. pneumoniae ATCC	K. pneumoniae ESBL
E. coli ATCC	-	**Yes**	**Yes**	No
E. coli ESBL	**Yes**	-	No	No
K. pneumoniae ATCC	**Yes**	No	-	No
K. pneumoniae ESBL	No	No	No	-

(a)

Significant differences:

	E. coli ATCC	E. coli ESBL	K. pneumoniae ATCC	K. pneumoniae ESBL
E. coli ATCC	-	**Yes**	**Yes**	**Yes**
E. coli ESBL	**Yes**	-	No	No
K. pneumoniae ATCC	**Yes**	No	-	No
K. pneumoniae ESBL	**Yes**	No	No	-

(b)

Figure S58. Comparison of total DNA/RNA concentration of all measurements for all tested strains with statistical analysis representation: (**a**) for 2 × MIC of nPt exposure, (**b**) for inoculums (untreated bacteria). P values are shown between comparing variables. If p < 0,05 the difference is considered as significant (Yes), if the p > 0,05 the difference is considered as not significant (No).

2.2. Extracellular proteins determination

Statistical test used: Friedman test (nonparametric comparison of *n* samples)
Statistical significance value (α) = 0,05

2.2.1. E. coli ATCC 10536

2.2.1.1. At 0 time point (of exposure to nPt)

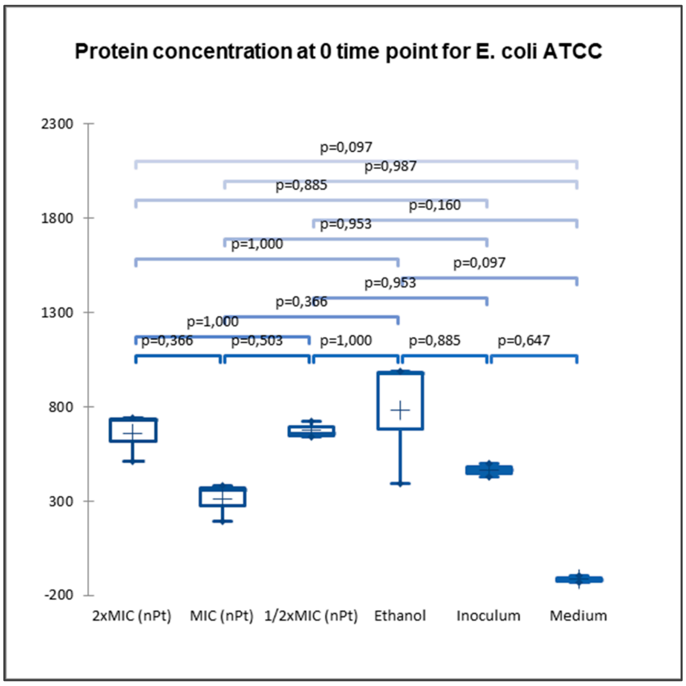

Figure S59. Graphical statistical analysis representation of determined protein concentrations obtained for E coli ATCC 10536 exposed to 2 × MIC, MIC, and 1/2 × MIC of nPt at zero time point. P values are shown between comparing variables.

Table S57. Statistical significance between comparing variables for the group of results from Figure S59. If p < 0,05 the difference is considered as significant (Yes), if the p > 0,05 the difference is considered as not significant (No).

Significant differences:

	2xMIC (nPt)	MIC (nPt)	1/2xMIC (nPt)	Ethanol	Inoculum	Medium
2xMIC (nPt)	-	No	No	No	No	No
MIC (nPt)	No	-	No	No	No	No
1/2xMIC (nPt)	No	No	-	No	No	No
Ethanol	No	No	No	-	No	No
Inoculum	No	No	No	No	-	No
Medium	No	No	No	No	No	-

2.2.1.2. After 1 hour (of exposure to nPt)

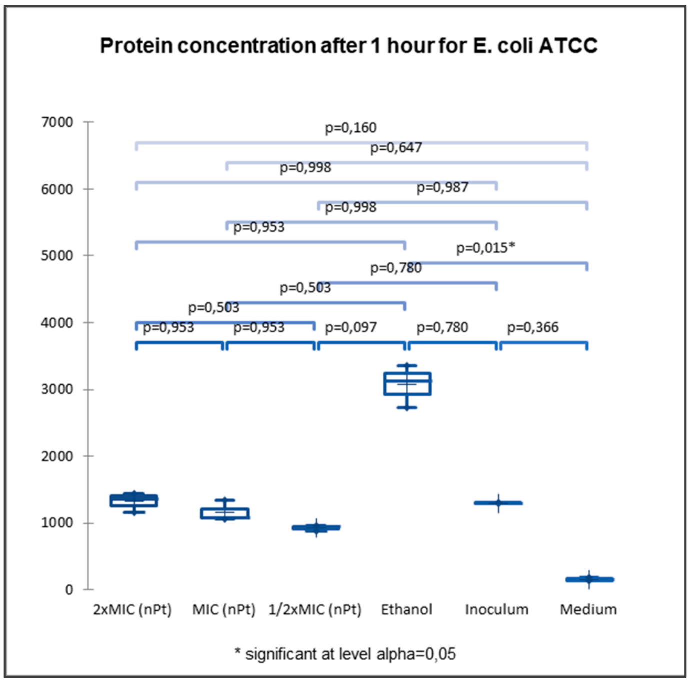

Figure S60. Graphical statistical analysis representation of determined protein concentrations obtained for E coli ATCC 10536 exposed to 2 × MIC, MIC, and 1/2 × MIC on nPt after 1 hour of exposure. P values are shown between comparing variables.

Table S58. Statistical significance between comparing variables for the group of results from Figure S60. If p < 0,05 the difference is considered as significant (Yes), if the p > 0,05 the difference is considered as not significant (No).

Significant differences:						
	2xMIC (nPt)	MIC (nPt)	1/2xMIC (nPt)	Ethanol	Inoculum	Medium
2xMIC (nPt)	-	No	No	No	No	No
MIC (nPt)	No	-	No	No	No	No
1/2xMIC (nPt)	No	No	-	No	No	No
Ethanol	No	No	No	-	No	**Yes**
Inoculum	No	No	No	No	-	No
Medium	No	No	No	**Yes**	No	-

2.2.1.3. After 3 hours (of exposure to nPt)

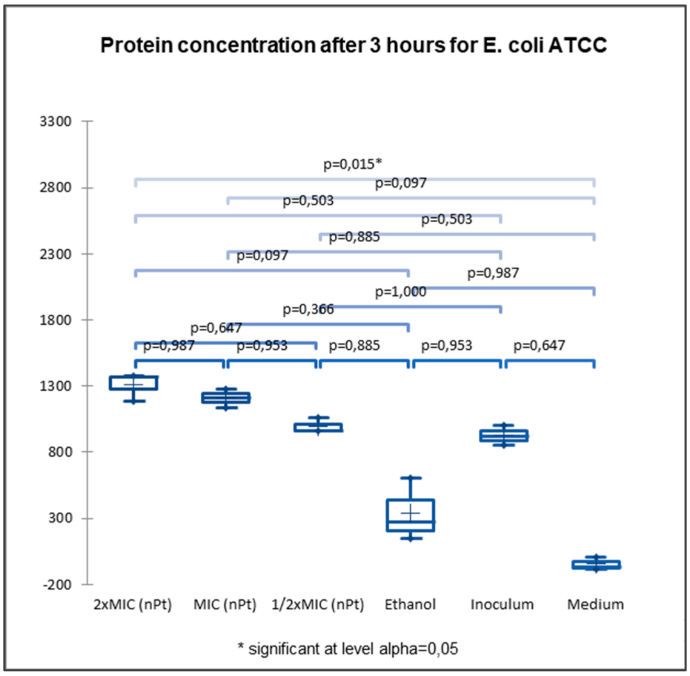

Figure S61. Graphical statistical analysis representation of determined protein concentrations obtained for E coli ATCC 10536 exposed to 2 × MIC, MIC, and 1/2 × MIC on nPt after 3 hours of exposure. P values are shown between comparing variables.

Table S59. Statistical significance between comparing variables for the group of results from Figure S61. If p < 0,05 the difference is considered as significant (Yes), if the p > 0,05 the difference is considered as not significant (No).

Significant differences:						
	2xMIC (nPt)	MIC (nPt)	1/2xMIC (nPt)	Ethanol	Inoculum	Medium
2xMIC (nPt)	-	No	No	No	No	**Yes**
MIC (nPt)	No	-	No	No	No	No
1/2xMIC (nPt)	No	No	-	No	No	No
Ethanol	No	No	No	-	No	No
Inoculum	No	No	No	No	-	No
Medium	**Yes**	No	No	No	No	-

2.2.1.4. After 6 hours (of exposure to nPt)

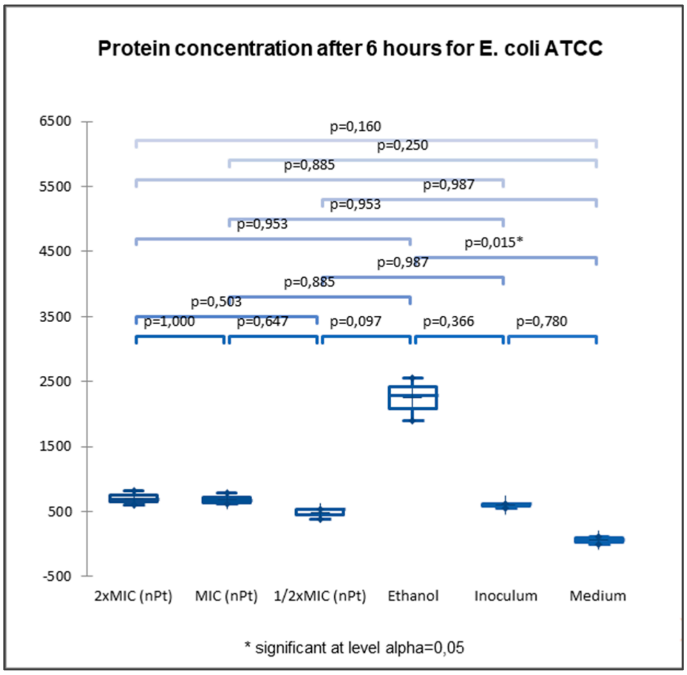

Figure S62. Graphical statistical analysis representation of determined protein concentrations obtained for E coli ATCC 10536 exposed to 2 × MIC, MIC, and 1/2 × MIC on nPt after 6 hours of exposure. P values are shown between comparing variables.

Table S60. Statistical significance between comparing variables for the group of results from Figure S62. If $p < 0,05$ the difference is considered as significant (Yes), if the $p > 0,05$ the difference is considered as not significant (No).

Significant differences:						
	2xMIC (nPt)	MIC (nPt)	1/2xMIC (nPt)	Ethanol	Inoculum	Medium
2xMIC (nPt)	-	No	No	No	No	No
MIC (nPt)	No	-	No	No	No	No
1/2xMIC (nPt)	No	No	-	No	No	No
Ethanol	No	No	No	-	No	**Yes**
Inoculum	No	No	No	No	-	No
Medium	No	No	No	**Yes**	No	-

2.2.1.5. After 18 hours (of exposure to nPt)

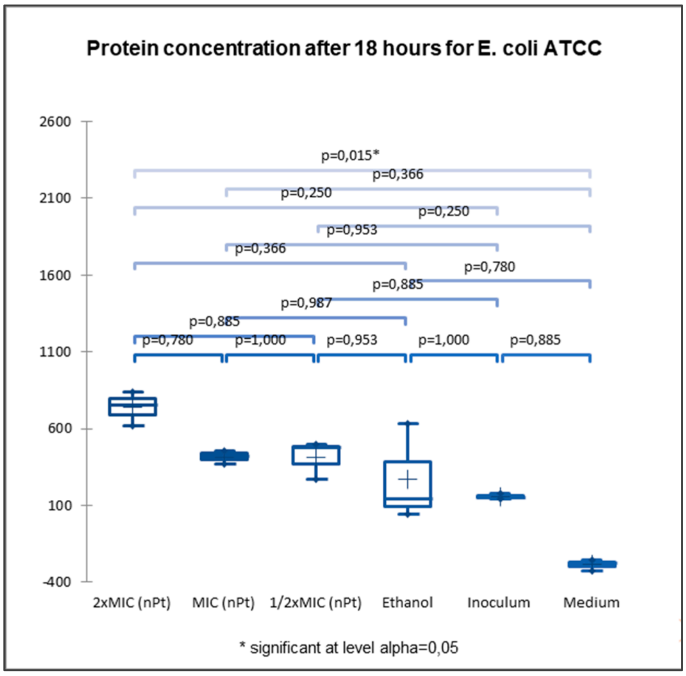

Figure S63. Graphical statistical analysis representation of determined protein concentrations obtained for E coli ATCC 10536 exposed to 2 × MIC, MIC, and 1/2 × MIC on nPt after 18 hours of exposure. P values are shown between comparing variables.

Table S61. Statistical significance between comparing variables for the group of results from Figure S63. If p < 0,05 the difference is considered as significant (Yes), if the p > 0,05 the difference is considered as not significant (No).

Significant differences:						
	2xMIC (nPt)	MIC (nPt)	1/2xMIC (nPt)	Ethanol	Inoculum	Medium
2xMIC (nPt)	-	No	No	No	No	**Yes**
MIC (nPt)	No	-	No	No	No	No
1/2xMIC (nPt)	No	No	-	No	No	No
Ethanol	No	No	No	-	No	No
Inoculum	No	No	No	No	-	No
Medium	**Yes**	No	No	No	No	-

2.2.1.6. After 24 hours (of exposure to nPt)

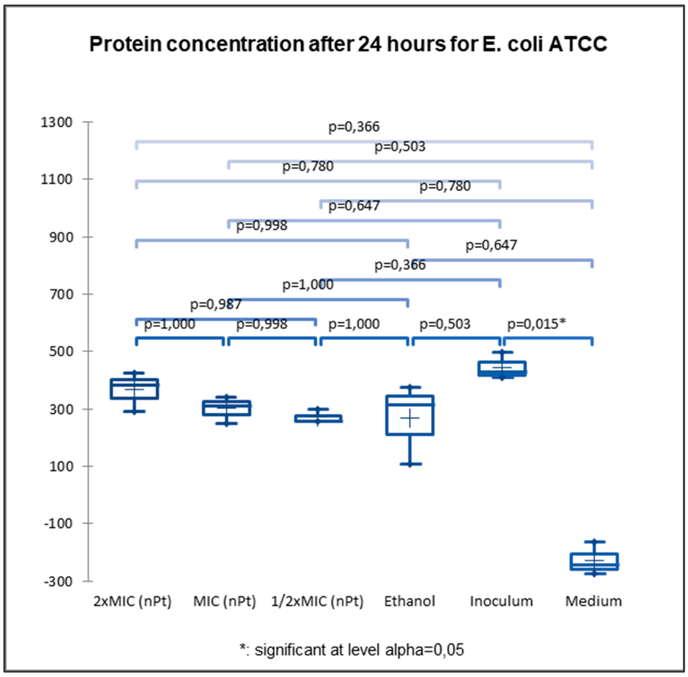

Figure S64. Graphical statistical analysis representation of determined protein concentrations obtained for E coli ATCC 10536 exposed to 2 × MIC, MIC, and 1/2 × MIC on nPt after 24 hours of exposure. P values are shown between comparing variables.

Table S62. Statistical significance between comparing variables for the group of results from Figure S64. If p < 0,05 the difference is considered as significant (Yes), if the p > 0,05 the difference is considered as not significant (No).

Significant differences:

	2xMIC (nPt)	MIC (nPt)	1/2xMIC (nPt)	Ethanol	Inoculum	Medium
2xMIC (nPt)	-	No	No	No	No	No
MIC (nPt)	No	-	No	No	No	No
1/2xMIC (nPt)	No	No	-	No	No	No
Ethanol	No	No	No	-	No	No
Inoculum	No	No	No	No	-	**Yes**
Medium	No	No	No	No	**Yes**	-

2.2.1.7. Total protein concentration statistical count (sum of results of all measurement time points)

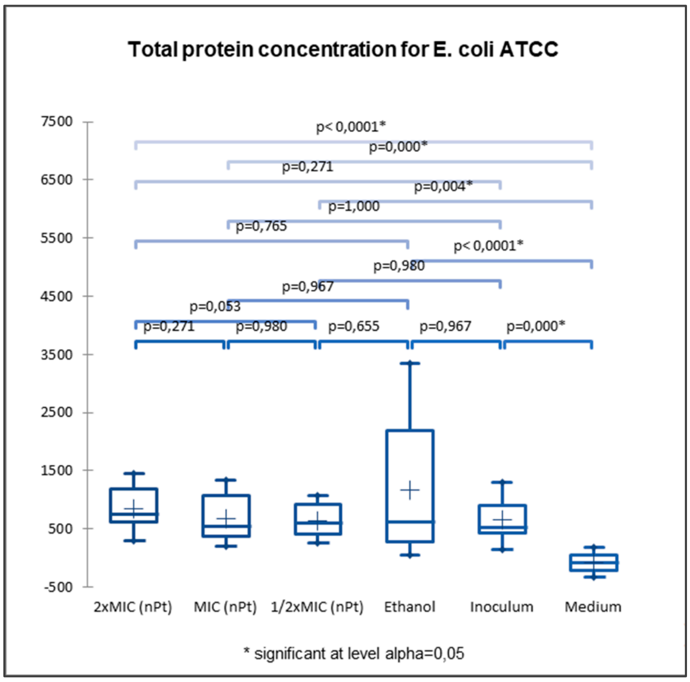

Figure S65. Graphical statistical analysis representation of determined total protein concentrations obtained for E coli ATCC 10536 exposed to 2 × MIC, MIC, and 1/2 × MIC of nPt as a sum of results of all measurement time points. P values are shown between comparing variables.

Table S63. Statistical significance between comparing variables for the group of results from Figure S65. If p < 0,05 the difference is considered as significant (Yes), if the p > 0,05 the difference is considered as not significant (No).

Significant differences:						
	2xMIC (nPt)	MIC (nPt)	1/2xMIC (nPt)	Ethanol	Inoculum	Medium
2xMIC (nPt)	-	No	No	No	No	**Yes**
MIC (nPt)	No	-	No	No	No	**Yes**
1/2xMIC (nPt)	No	No	-	No	No	**Yes**
Ethanol	No	No	No	-	No	**Yes**
Inoculum	No	No	No	No	-	**Yes**
Medium	**Yes**	**Yes**	**Yes**	**Yes**	**Yes**	-

2.2.2. E. coli ESBL+ MFBF 12795

2.2.2.1. At 0 time point (of exposure to nPt)

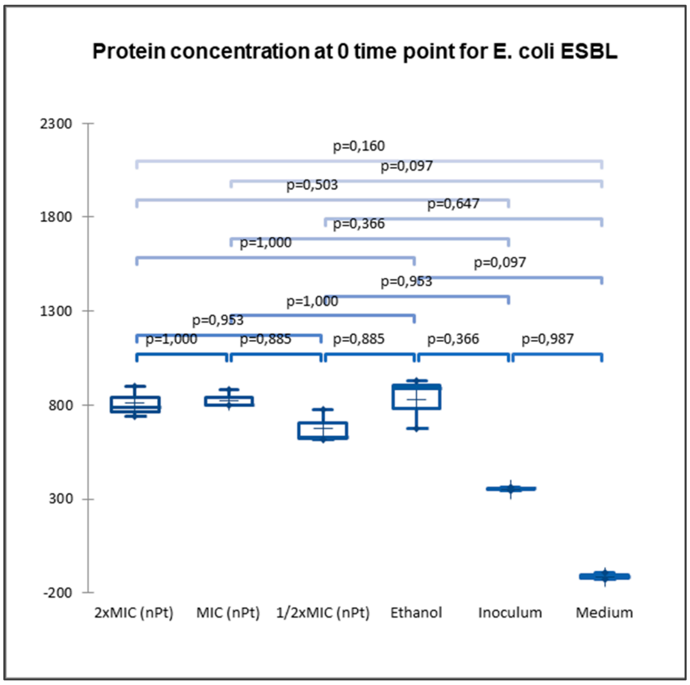

Figure S66. Graphical statistical analysis representation of determined protein concentrations obtained for E coli ESBL+ MFBF 12795 exposed to 2 × MIC, MIC, and 1/2 × MIC of nPt at zero time point. P values are shown between comparing variables.

Table S64. Statistical significance between comparing variables for the group of results from Figure S66. If p < 0,05 the difference is considered as significant (Yes), if the p > 0,05 the difference is considered as not significant (No).

Significant differences:						
	2xMIC (nPt)	MIC (nPt)	1/2xMIC (nPt)	Ethanol	Inoculum	Medium
2xMIC (nPt)	-	No	No	No	No	No
MIC (nPt)	No	-	No	No	No	No
1/2xMIC (nPt)	No	No	-	No	No	No
Ethanol	No	No	No	-	No	No
Inoculum	No	No	No	No	-	No
Medium	No	No	No	No	No	-

2.2.2.2. After 1 hour (of exposure to nPt)

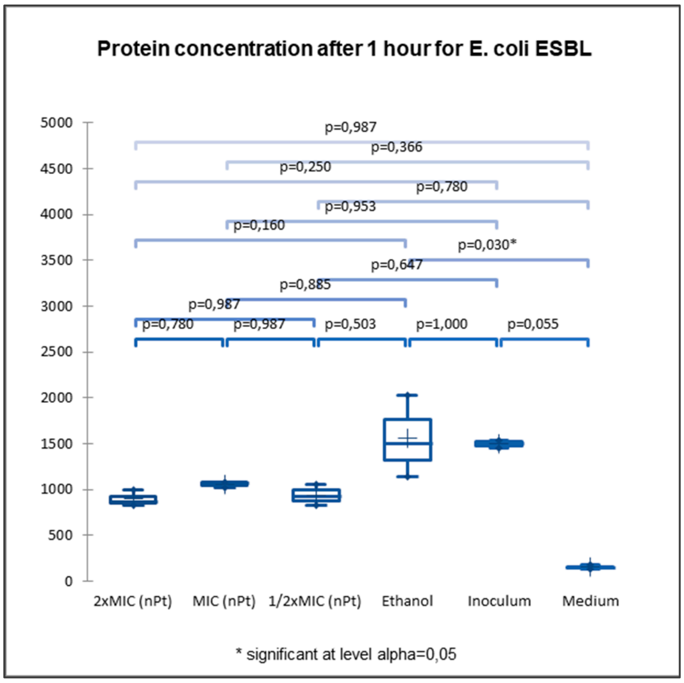

Figure S67. Graphical statistical analysis representation of determined protein concentrations obtained for E coli ESBL+ MFBF 12795 exposed to 2 × MIC, MIC, and 1/2 × MIC of nPt after 1 hour of exposure. P values are shown between comparing variables.

Table S65. Statistical significance between comparing variables for the group of results from Figure S67. If p < 0,05 the difference is considered as significant (Yes), if the p > 0,05 the difference is considered as not significant (No).

Significant differences:

	2xMIC (nPt)	MIC (nPt)	1/2xMIC (nPt)	Ethanol	Inoculum	Medium
2xMIC (nPt)	-	No	No	No	No	No
MIC (nPt)	No	-	No	No	No	No
1/2xMIC (nPt)	No	No	-	No	No	No
Ethanol	No	No	No	-	No	**Yes**
Inoculum	No	No	No	No	-	No
Medium	No	No	No	**Yes**	No	-

2.2.2.3. After 3 hours (of exposure to nPt)

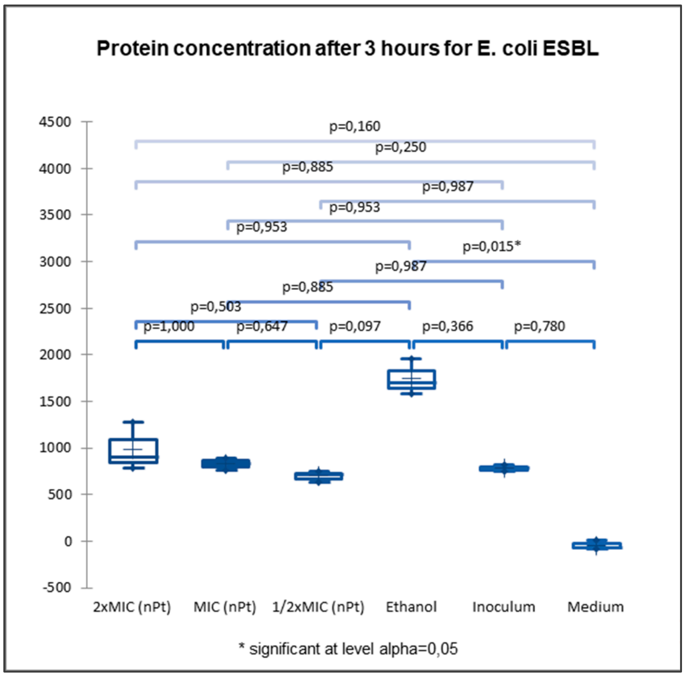

Figure S68. Graphical statistical analysis representation of determined protein concentrations obtained for E coli ESBL+ MFBF 12795 exposed to 2 × MIC, MIC, and 1/2 × MIC of nPt after 3 hours of exposure. P values are shown between comparing variables.

Table S66. Statistical significance between comparing variables for the group of results from Figure S68. If p < 0,05 the difference is considered as significant (Yes), if the p > 0,05 the difference is considered as not significant (No).

Significant differences:						
	2xMIC (nPt)	MIC (nPt)	1/2xMIC (nPt)	Ethanol	Inoculum	Medium
2xMIC (nPt)	-	No	No	No	No	No
MIC (nPt)	No	-	No	No	No	No
1/2xMIC (nPt)	No	No	-	No	No	No
Ethanol	No	No	No	-	No	**Yes**
Inoculum	No	No	No	No	-	No
Medium	No	No	No	**Yes**	No	-

2.2.2.4. After 6 hours (of exposure to nPt)

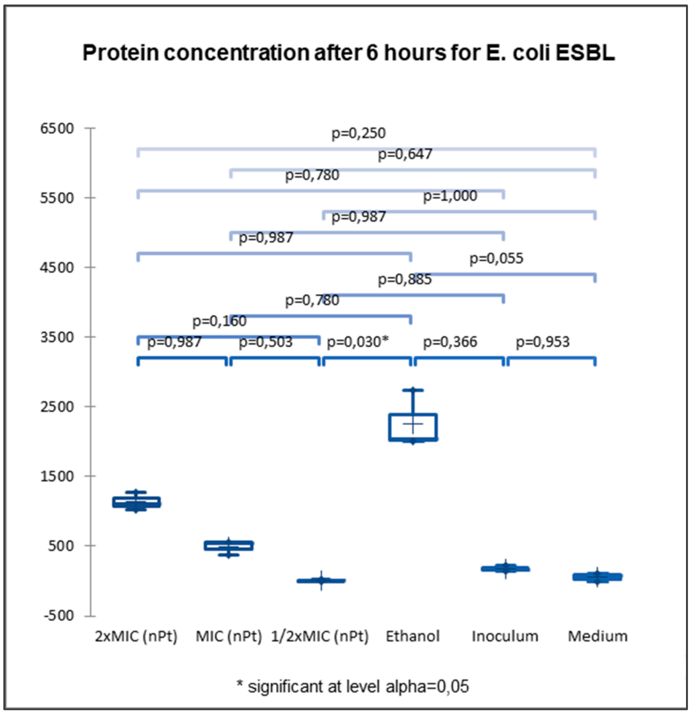

Figure S69. Graphical statistical analysis representation of determined protein concentrations obtained for E coli ESBL+ MFBF 12795 exposed to 2 × MIC, MIC, and 1/2 × MIC of nPt after 6 hours of exposure. P values are shown between comparing variables.

Table S67. Statistical significance between comparing variables for the group of results from Figure S69. If p < 0,05 the difference is considered as significant (Yes), if the p > 0,05 the difference is considered as not significant (No).

Significant differences:

	2xMIC (nPt)	MIC (nPt)	1/2xMIC (nPt)	Ethanol	Inoculum	Medium
2xMIC (nPt)	-	No	No	No	No	No
MIC (nPt)	No	-	No	No	No	No
1/2xMIC (nPt)	No	No	-	**Yes**	No	No
Ethanol	No	No	**Yes**	-	No	No
Inoculum	No	No	No	No	-	No
Medium	No	No	No	No	No	-

2.2.2.5. After 18 hours (of exposure to nPt)

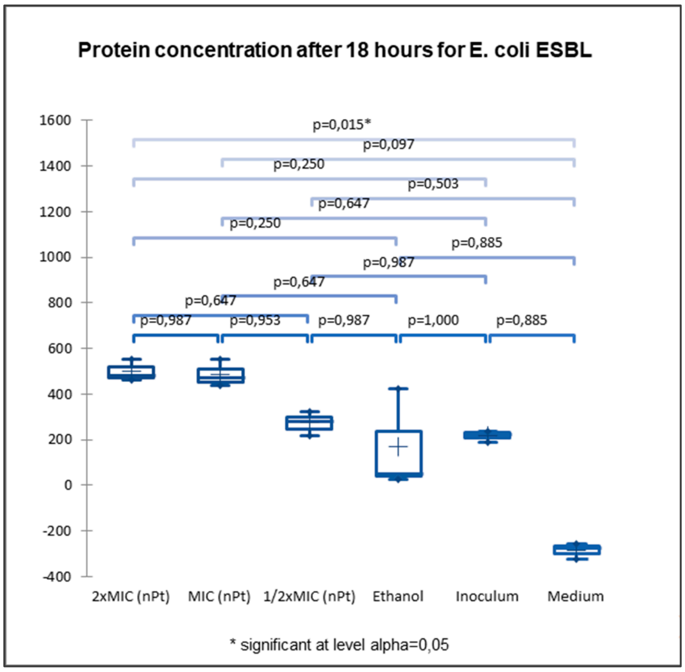

Figure S70. Graphical statistical analysis representation of determined protein concentrations obtained for E coli ESBL+ MFBF 12795 exposed to 2 × MIC, MIC, and 1/2 × MIC of nPt after 18 hours of exposure. P values are shown between comparing variables.

Table S68. Statistical significance between comparing variables for the group of results from Figure S70. If p < 0,05 the difference is considered as significant (Yes), if the p > 0,05 the difference is considered as not significant (No).

Significant differences:						
	2xMIC (nPt)	MIC (nPt)	1/2xMIC (nPt)	Ethanol	Inoculum	Medium
2xMIC (nPt)	-	No	No	No	No	**Yes**
MIC (nPt)	No	-	No	No	No	No
1/2xMIC (nPt)	No	No	-	No	No	No
Ethanol	No	No	No	-	No	No
Inoculum	No	No	No	No	-	No
Medium	**Yes**	No	No	No	No	-

2.2.2.6. After 24 hours (of exposure to nPt)

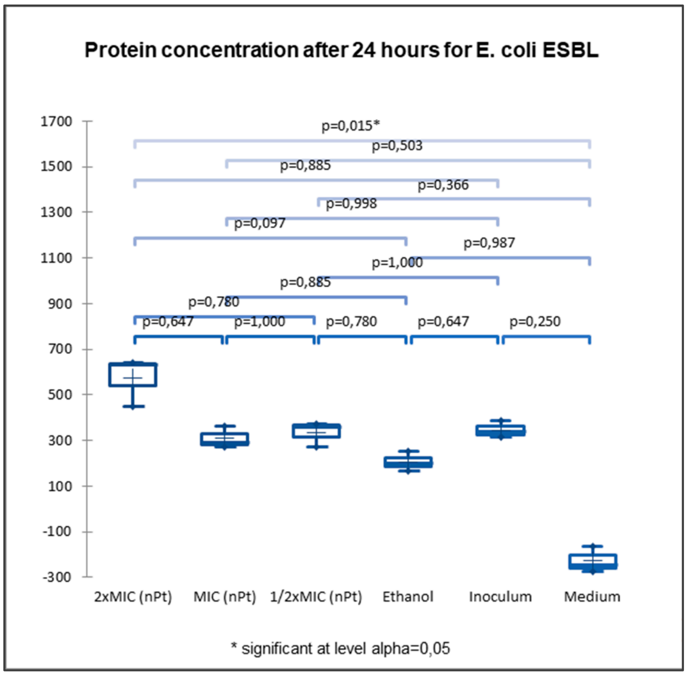

Figure S71. Graphical statistical analysis representation of determined protein concentrations obtained for E coli ESBL+ MFBF 12795 exposed to 2 × MIC, MIC, and 1/2 × MIC of nPt after 24 hours of exposure. P values are shown between comparing variables.

Table S69. Statistical significance between comparing variables for the group of results from Figure S71. If p < 0,05 the difference is considered as significant (Yes), if the p > 0,05 the difference is considered as not significant (No).

Significant differences:

	2xMIC (nPt)	MIC (nPt)	1/2xMIC (nPt)	Ethanol	Inoculum	Medium
2xMIC (nPt)	-	No	No	No	No	**Yes**
MIC (nPt)	No	-	No	No	No	No
1/2xMIC (nPt)	No	No	-	No	No	No
Ethanol	No	No	No	-	No	No
Inoculum	No	No	No	No	-	No
Medium	**Yes**	No	No	No	No	-

2.2.2.7. Total protein concentration statistical count (sum of results of all measurement time points)

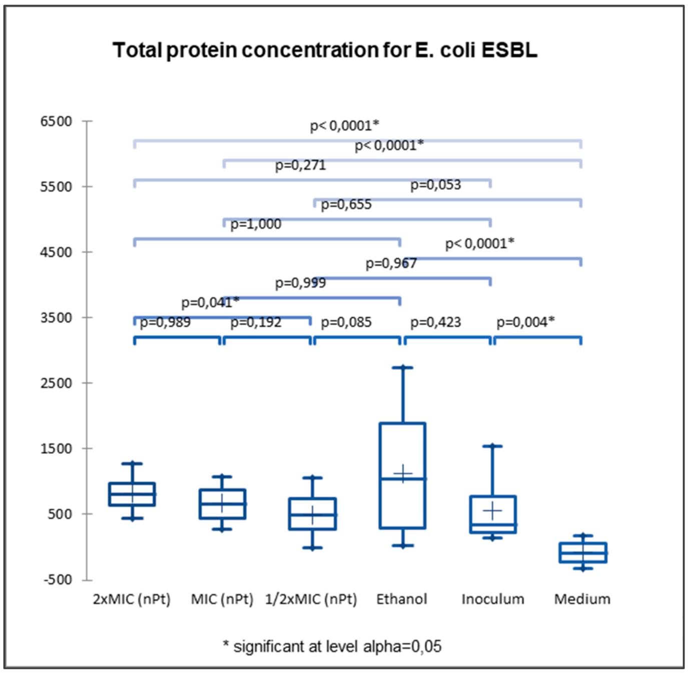

Figure S72. Graphical statistical analysis representation of determined protein concentrations obtained for E coli ESBL+ MFBF 12795 exposed to 2 × MIC, MIC, and 1/2 × MIC of nPt as a sum of results of all measurement time points. P values are shown between comparing variables.

Table S70. Statistical significance between comparing variables for the group of results from Figure S72. If p < 0,05 the difference is considered as significant (Yes), if the p > 0,05 the difference is considered as not significant (No).

Significant differences:

	2xMIC (nPt)	MIC (nPt)	1/2xMIC (nPt)	Ethanol	Inoculum	Medium
2xMIC (nPt)	-	No	**Yes**	No	No	**Yes**
MIC (nPt)	No	-	No	No	No	**Yes**
1/2xMIC (nPt)	**Yes**	No	-	No	No	No
Ethanol	No	No	No	-	No	**Yes**
Inoculum	No	No	No	No	-	**Yes**
Medium	**Yes**	**Yes**	No	**Yes**	**Yes**	-

2.2.3. K. pneumoniae ATCC 700603

2.2.3.1. At 0 time point (of exposure to nPt)

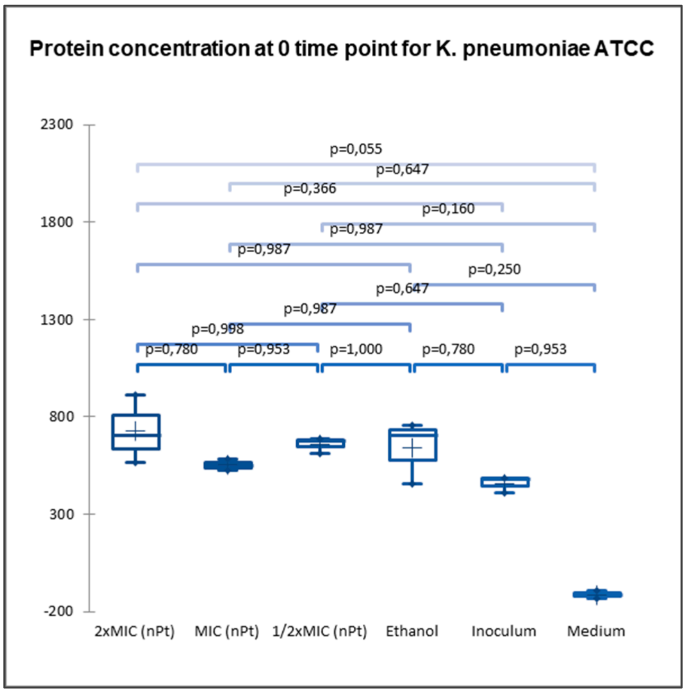

Figure S73. Graphical statistical analysis representation of determined protein concentrations obtained for K. pneumoniae ATCC 700603 exposed to 2 × MIC, MIC, and 1/2 × MIC of nPt at zero time point. P values are shown between comparing variables.

Table S71. Statistical significance between comparing variables for the group of results from Figure S73. If p < 0,05 the difference is considered as significant (Yes), if the p > 0,05 the difference is considered as not significant (No).

Significant differences:						
	2xMIC (nPt)	MIC (nPt)	1/2xMIC (nPt)	Ethanol	Inoculum	Medium
2xMIC (nPt)	-	No	No	No	No	No
MIC (nPt)	No	-	No	No	No	No
1/2xMIC (nPt)	No	No	-	No	No	No
Ethanol	No	No	No	-	No	No
Inoculum	No	No	No	No	-	No
Medium	No	No	No	No	No	-

2.2.3.2. After 1 hour (of exposure to nPt)

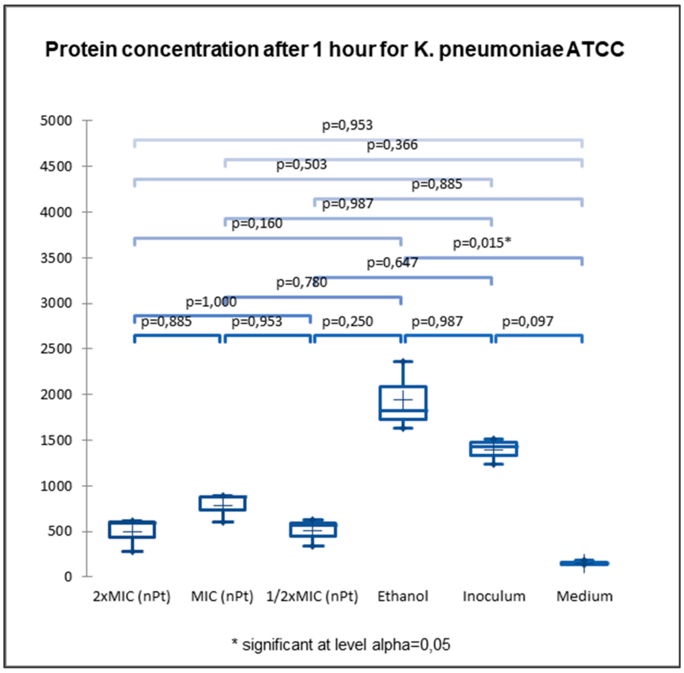

Figure S74. Graphical statistical analysis representation of determined protein concentrations obtained for K. pneumoniae ATCC 700603 exposed to 2 × MIC, MIC, and 1/2 × MIC of nPt after 1 hour of exposure. P values are shown between comparing variables.

Table S72. Statistical significance between comparing variables for the group of results from Figure S74. If p < 0,05 the difference is considered as significant (Yes), if the p > 0,05 the difference is considered as not significant (No).

Significant differences:						
	2xMIC (nPt)	MIC (nPt)	1/2xMIC (nPt)	Ethanol	Inoculum	Medium
2xMIC (nPt)	-	No	No	No	No	No
MIC (nPt)	No	-	No	No	No	No
1/2xMIC (nPt)	No	No	-	No	No	No
Ethanol	No	No	No	-	No	**Yes**
Inoculum	No	No	No	No	-	No
Medium	No	No	No	**Yes**	No	-

2.2.3.3. After 3 hours (of exposure to nPt)

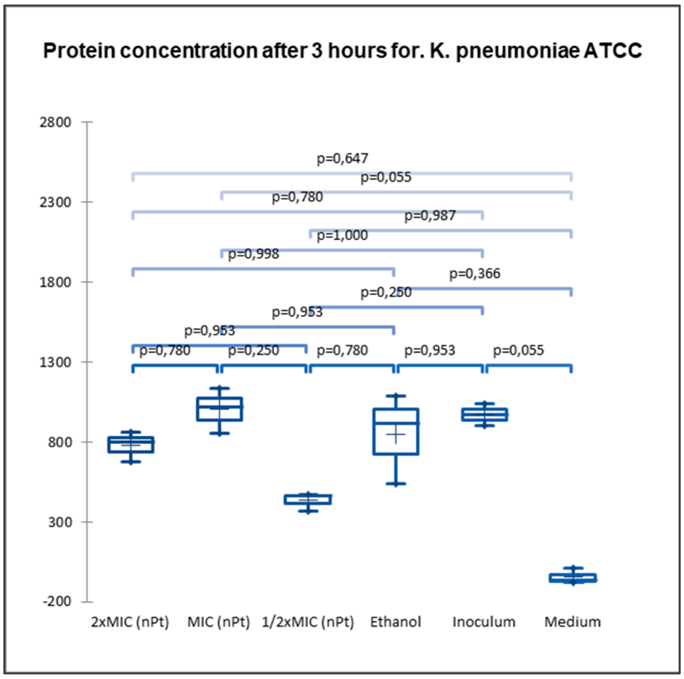

Figure S75S. Graphical statistical analysis representation of determined protein concentrations obtained for K. pneumoniae ATCC 700603 exposed to 2 × MIC, MIC, and 1/2 × MIC of nPt after 3 hours of exposure. P values are shown between comparing variables.

Table S73. Statistical significance between comparing variables for the group of results from Figure S75. If p < 0,05 the difference is considered as significant (Yes), if the p > 0,05 the difference is considered as not significant (No).

Significant differences:

	2xMIC (nPt)	MIC (nPt)	1/2xMIC (nPt)	Ethanol	Inoculum	Medium
2xMIC (nPt)	-	No	No	No	No	No
MIC (nPt)	No	-	No	No	No	No
1/2xMIC (nPt)	No	No	-	No	No	No
Ethanol	No	No	No	-	No	No
Inoculum	No	No	No	No	-	No
Medium	No	No	No	No	No	-

2.2.3.4. After 6 hours (of exposure to nPt)

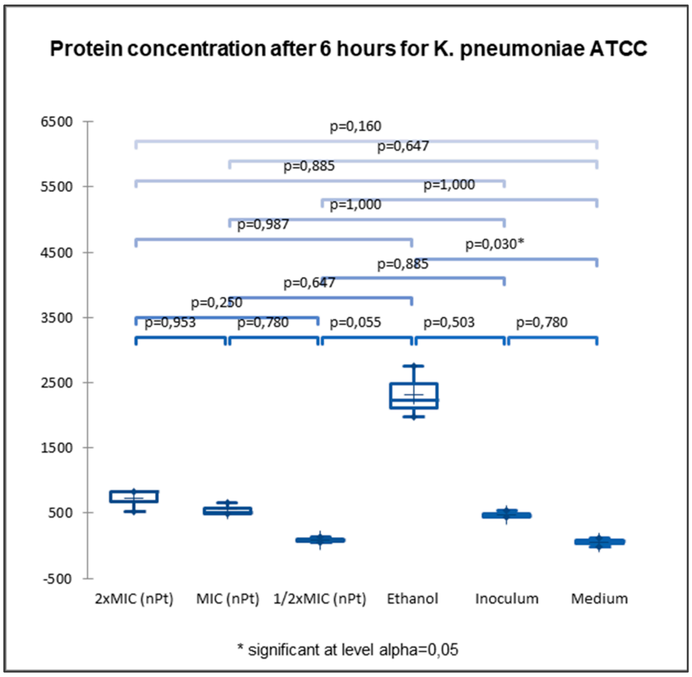

Figure S76. Graphical statistical analysis representation of determined protein concentrations obtained for K. pneumoniae ATCC 700603 exposed to 2 × MIC, MIC, and 1/2 × MIC of nPt after 6 hours of exposure. P values are shown between comparing variables.

Table S74. Statistical significance between comparing variables for the group of results from Figure S76. If p < 0,05 the difference is considered as significant (Yes), if the p > 0,05 the difference is considered as not significant (No).

Significant differences:

	2xMIC (nPt)	MIC (nPt)	1/2xMIC (nPt)	Ethanol	Inoculum	Medium
2xMIC (nPt)	-	No	No	No	No	No
MIC (nPt)	No	-	No	No	No	No
1/2xMIC (nPt)	No	No	-	No	No	No
Ethanol	No	No	No	-	No	**Yes**
Inoculum	No	No	No	No	-	No
Medium	No	No	No	**Yes**	No	-

2.2.3.5. After 18 hours (of exposure to nPt)

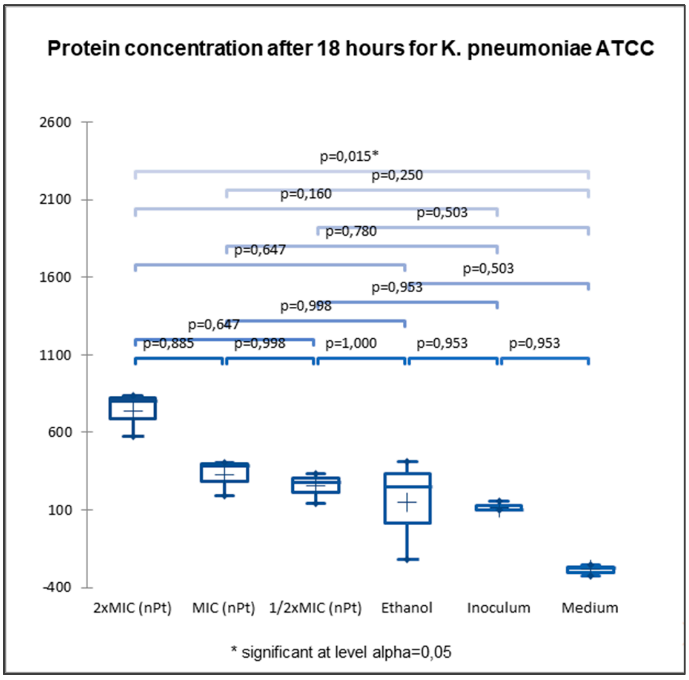

Figure S77. Graphical statistical analysis representation of determined protein concentrations obtained for K. pneumoniae ATCC 700603 exposed to 2 × MIC, MIC, and 1/2 × MIC of nPt after 18 hours of exposure. P values are shown between comparing variables.

Table S75S. Statistical significance between comparing variables for the group of results from Figure S77. If p < 0,05 the difference is considered as significant (Yes), if the p > 0,05 the difference is considered as not significant (No).

Significant differences:						
	2xMIC (nPt)	MIC (nPt)	1/2xMIC (nPt)	Ethanol	Inoculum	Medium
2xMIC (nPt)	-	No	No	No	No	**Yes**
MIC (nPt)	No	-	No	No	No	No
1/2xMIC (nPt)	No	No	-	No	No	No
Ethanol	No	No	No	-	No	No
Inoculum	No	No	No	No	-	No
Medium	**Yes**	No	No	No	No	-

2.2.3.6. After 24 hours (of exposure to nPt)

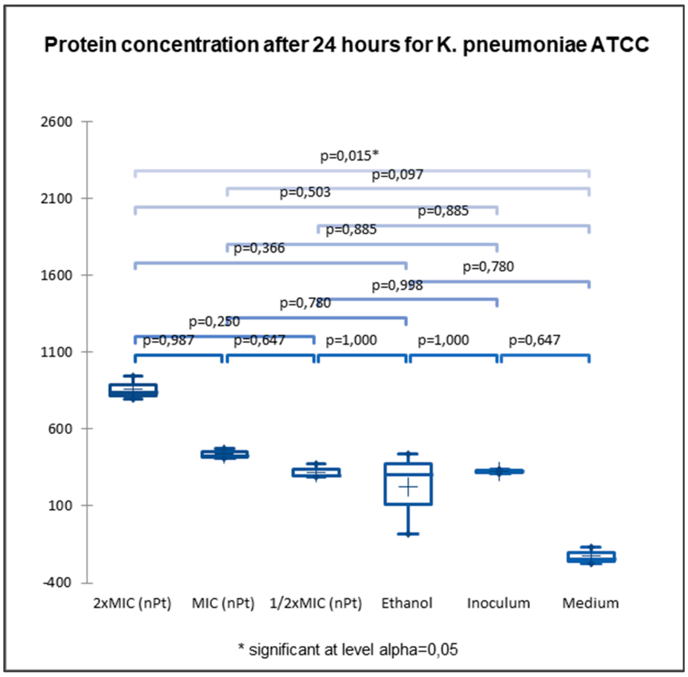

Figure S78. Graphical statistical analysis representation of determined protein concentrations obtained for K. pneumoniae ATCC 700603 exposed to 2 × MIC, MIC, and 1/2 × MIC of nPt after 24 hours of exposure. P values are shown between comparing variables.

Table S76. Statistical significance between comparing variables for the group of results from Figure S78. If p < 0,05 the difference is considered as significant (Yes), if the p > 0,05 the difference is considered as not significant (No).

Significant differences:						
	2xMIC (nPt)	MIC (nPt)	1/2xMIC (nPt)	Ethanol	Inoculum	Medium
2xMIC (nPt)	-	No	No	No	No	**Yes**
MIC (nPt)	No	-	No	No	No	No
1/2xMIC (nPt)	No	No	-	No	No	No
Ethanol	No	No	No	-	No	No
Inoculum	No	No	No	No	-	No
Medium	**Yes**	No	No	No	No	-

2.2.3.7. Total protein concentration statistical count (sum of results of all measurement time points)

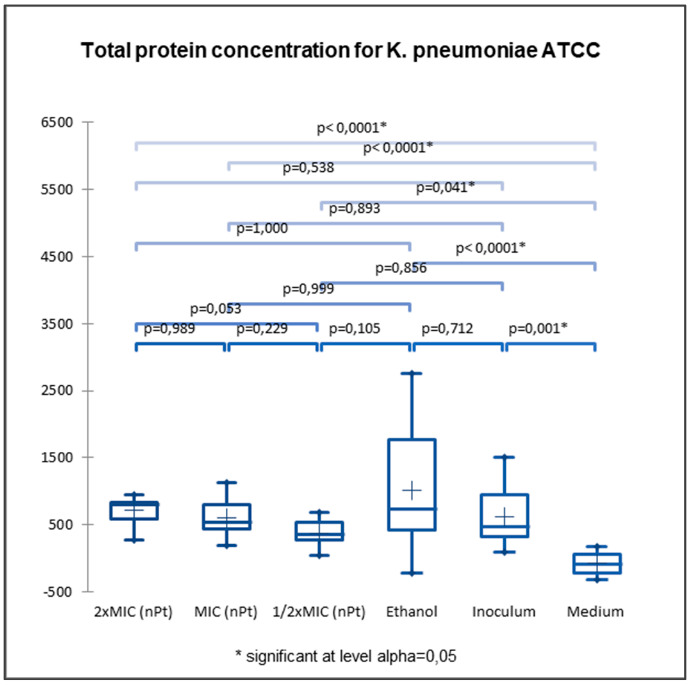

Figure S79. Graphical statistical analysis representation of determined protein concentrations obtained for K. pneumoniae ATCC 70060 exposed to 2 × MIC, MIC, and 1/2 × MIC of nPt as a sum of results of all measurement time points. P values are shown between comparing variables.

Table S77. Statistical significance between comparing variables for the group of results from Figure S79. If p < 0,05 the difference is considered as significant (Yes), if the p > 0,05 the difference is considered as not significant (No).

Significant differences:						
	2xMIC (nPt)	MIC (nPt)	1/2xMIC (nPt)	Ethanol	Inoculum	Medium
2xMIC (nPt)	-	No	No	No	No	**Yes**
MIC (nPt)	No	-	No	No	No	**Yes**
1/2xMIC (nPt)	No	No	-	No	No	**Yes**
Ethanol	No	No	No	-	No	**Yes**
Inoculum	No	No	No	No	-	**Yes**
Medium	**Yes**	**Yes**	**Yes**	**Yes**	**Yes**	-

2.2.4. K. pneumoniae ESBL+ MFBF 10690

2.2.4.1. At 0 time point (of exposure to nPt)

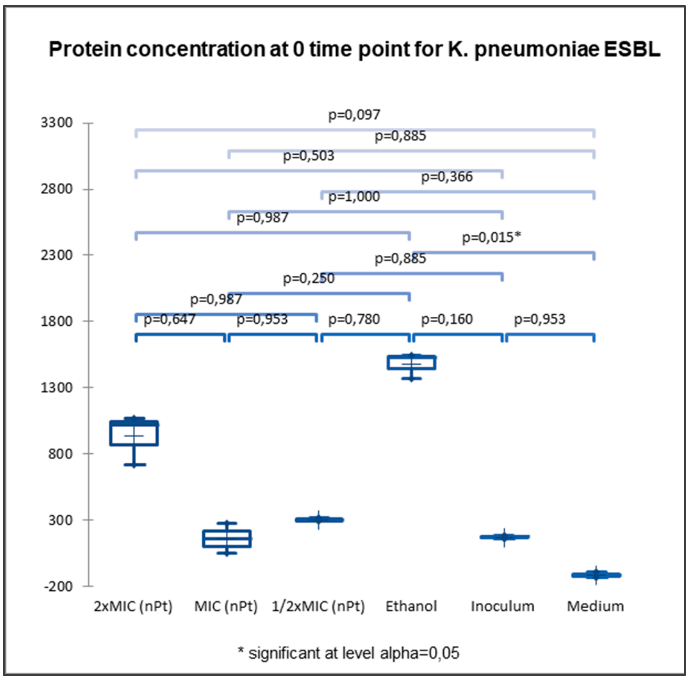

Figure S80. Graphical statistical analysis representation of determined protein concentrations obtained for K. pneumoniae ESBL+ MFBF 10690 exposed to 2 × MIC, MIC, and 1/2 × MIC of nPt at zero time point. P values are shown between comparing variables.

Table S78. Statistical significance between comparing variables for the group of results from Figure S80. If p < 0,05 the difference is considered as significant (Yes), if the p > 0,05 the difference is considered as not significant (No).

Significant differences:						
	2xMIC (nPt)	MIC (nPt)	1/2xMIC (nPt)	Ethanol	Inoculum	Medium
2xMIC (nPt)	-	No	No	No	No	No
MIC (nPt)	No	-	No	No	No	No
1/2xMIC (nPt)	No	No	-	No	No	No
Ethanol	No	No	No	-	No	**Yes**
Inoculum	No	No	No	No	-	No
Medium	No	No	No	**Yes**	No	-

2.2.4.2. After 1 hour (of exposure to nPt)

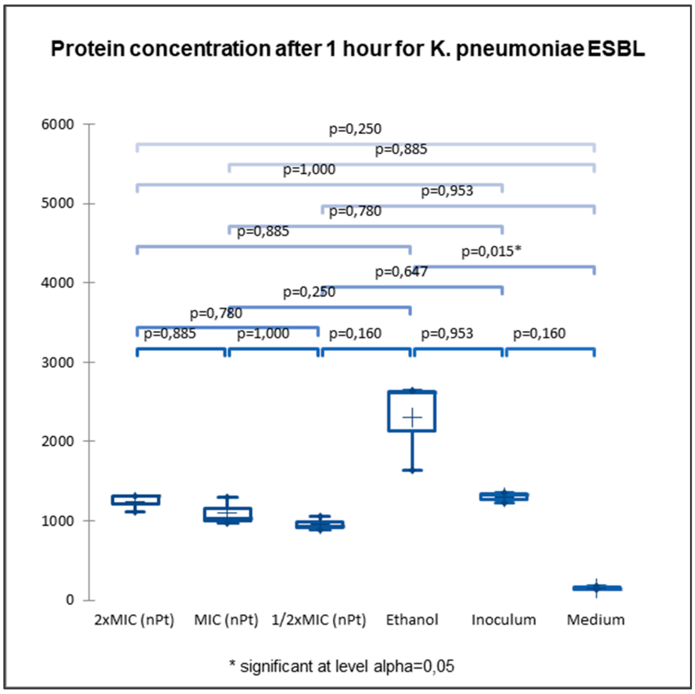

Figure S81. Graphical statistical analysis representation of determined protein concentrations obtained for K. pneumoniae ESBL+ MFBF 10690 exposed to 2 × MIC, MIC, and 1/2 × MIC of nPt after 1 hour of exposure. P values are shown between comparing variables.

Table S79. Statistical significance between comparing variables for the group of results from Figure S81. If p < 0,05 the difference is considered as significant (Yes), if the p > 0,05 the difference is considered as not significant (No).

Significant differences:

	2xMIC (nPt)	MIC (nPt)	1/2xMIC (nPt)	Ethanol	Inoculum	Medium
2xMIC (nPt)	-	No	No	No	No	No
MIC (nPt)	No	-	No	No	No	No
1/2xMIC (nPt)	No	No	-	No	No	No
Ethanol	No	No	No	-	No	**Yes**
Inoculum	No	No	No	No	-	No
Medium	No	No	No	**Yes**	No	-

2.2.4.3. After 3 hours (of exposure to nPt)

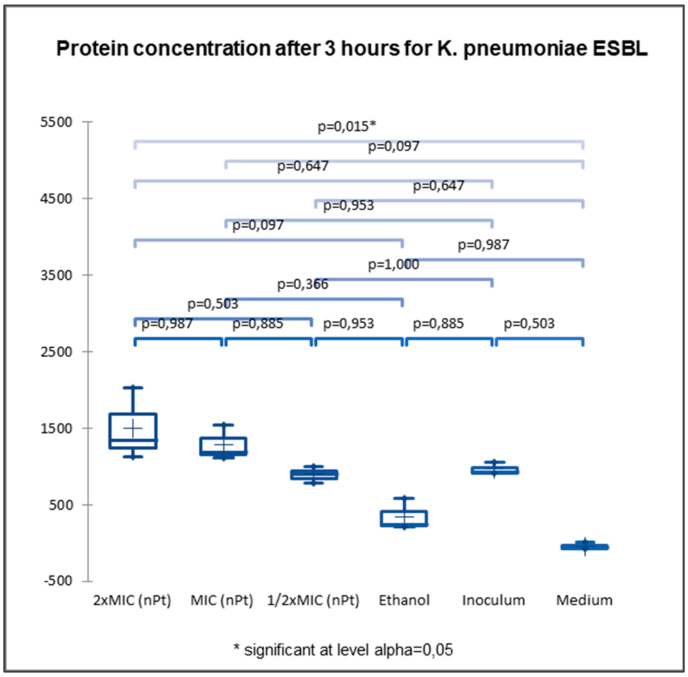

Figure S82. Graphical statistical analysis representation of determined protein concentrations obtained for K. pneumoniae ESBL+ MFBF 10690 exposed to 2 × MIC, MIC, and 1/2 × MIC of nPt after 3 hours of exposure. P values are shown between comparing variables.

Table S80. Statistical significance between comparing variables for the group of results from Figure S82. If p < 0,05 the difference is considered as significant (Yes), if the p > 0,05 the difference is considered as not significant (No).

Significant differences:

	2xMIC (nPt)	MIC (nPt)	1/2xMIC (nPt)	Ethanol	Inoculum	Medium
2xMIC (nPt)	-	No	No	No	No	**Yes**
MIC (nPt)	No	-	No	No	No	No
1/2xMIC (nPt)	No	No	-	No	No	No
Ethanol	No	No	No	-	No	No
Inoculum	No	No	No	No	-	No
Medium	**Yes**	No	No	No	No	-

2.2.4.4. After 6 hours (of exposure to nPt)

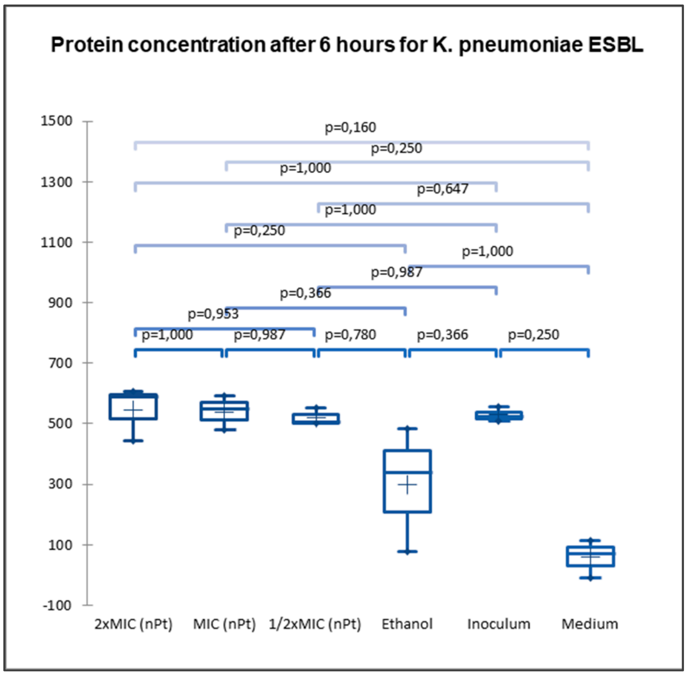

Figure S83. Graphical statistical analysis representation of determined protein concentrations obtained for K. pneumoniae ESBL+ MFBF 10690 exposed to 2 × MIC, MIC, and 1/2 × MIC of nPt after 6 hours of exposure. P values are shown between comparing variables.

Table S81. Statistical significance between comparing variables for the group of results from Figure S83. If p < 0,05 the difference is considered as significant (Yes), if the p > 0,05 the difference is considered as not significant (No).

Significant differences:						
	2xMIC (nPt)	MIC (nPt)	1/2xMIC (nPt)	Ethanol	Inoculum	Medium
2xMIC (nPt)	-	No	No	No	No	No
MIC (nPt)	No	-	No	No	No	No
1/2xMIC (nPt)	No	No	-	No	No	No
Ethanol	No	No	No	-	No	No
Inoculum	No	No	No	No	-	No
Medium	No	No	No	No	No	-

2.2.4.5. After 18 hours (of exposure to nPt)

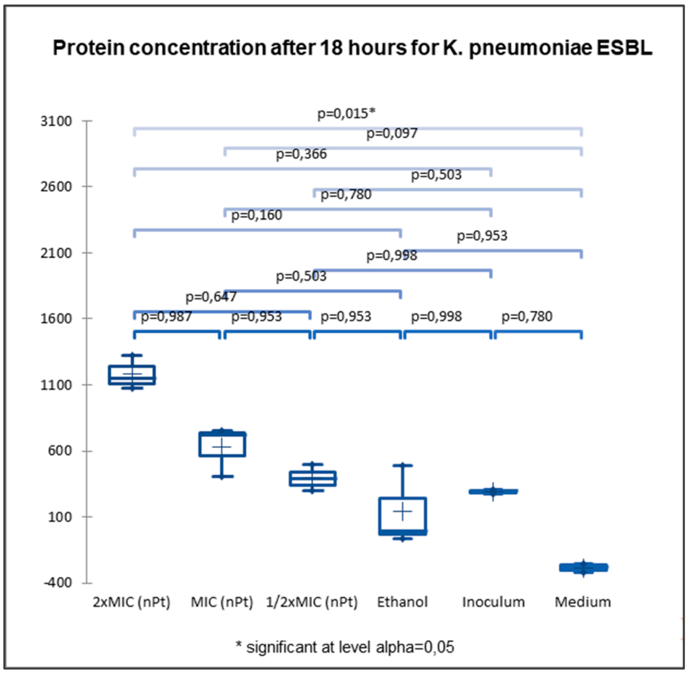

Figure S84. Graphical statistical analysis representation of determined protein concentrations obtained for K. pneumoniae ESBL+ MFBF 10690 exposed to 2 × MIC, MIC, and 1/2 × MIC of nPt after 18 hours of exposure. P values are shown between comparing variables.

Table S82. Statistical significance between comparing variables for the group of results from Figure S84. If p < 0,05 the difference is considered as significant (Yes), if the p > 0,05 the difference is considered as not significant (No).

Significant differences:

	2xMIC (nPt)	MIC (nPt)	1/2xMIC (nPt)	Ethanol	Inoculum	Medium
2xMIC (nPt)	-	No	No	No	No	**Yes**
MIC (nPt)	No	-	No	No	No	No
1/2xMIC (nPt)	No	No	-	No	No	No
Ethanol	No	No	No	-	No	No
Inoculum	No	No	No	No	-	No
Medium	**Yes**	No	No	No	No	-

2.2.4.6. After 24 hours (of exposure to nPt)

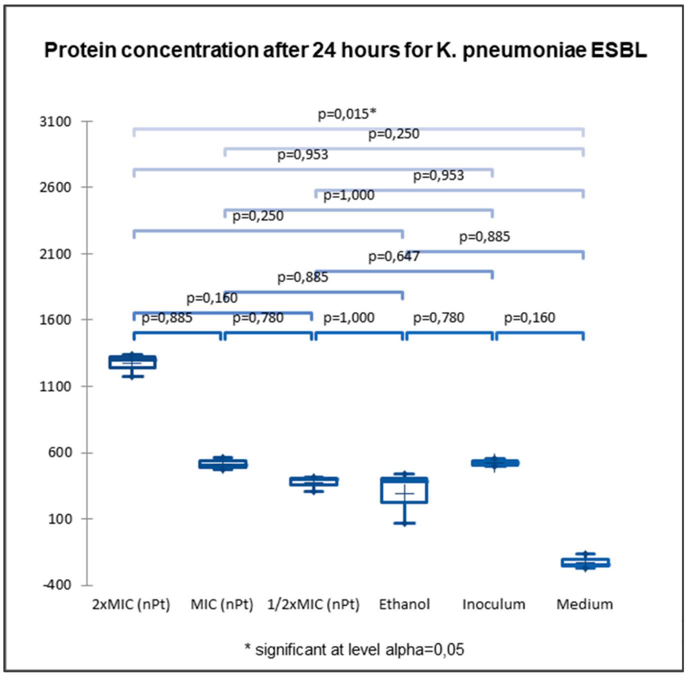

Figure S85. Graphical statistical analysis representation of determined protein concentrations obtained for K. pneumoniae ESBL+ MFBF 10690 exposed to 2 × MIC, MIC, and 1/2 × MIC of nPt after 24 hours of exposure. P values are shown between comparing variables.

Table S83. Statistical significance between comparing variables for the group of results from Figure S85. If p < 0,05 the difference is considered as significant (Yes), if the p > 0,05 the difference is considered as not significant (No).

Significant differences:

	2xMIC (nPt)	MIC (nPt)	1/2xMIC (nPt)	Ethanol	Inoculum	Medium
2xMIC (nPt)	-	No	No	No	No	**Yes**
MIC (nPt)	No	-	No	No	No	No
1/2xMIC (nPt)	No	No	-	No	No	No
Ethanol	No	No	No	-	No	No
Inoculum	No	No	No	No	-	No
Medium	**Yes**	No	No	No	No	-

2.2.4.7. Total protein concentration statistical count (sum of results of all measurement time points)

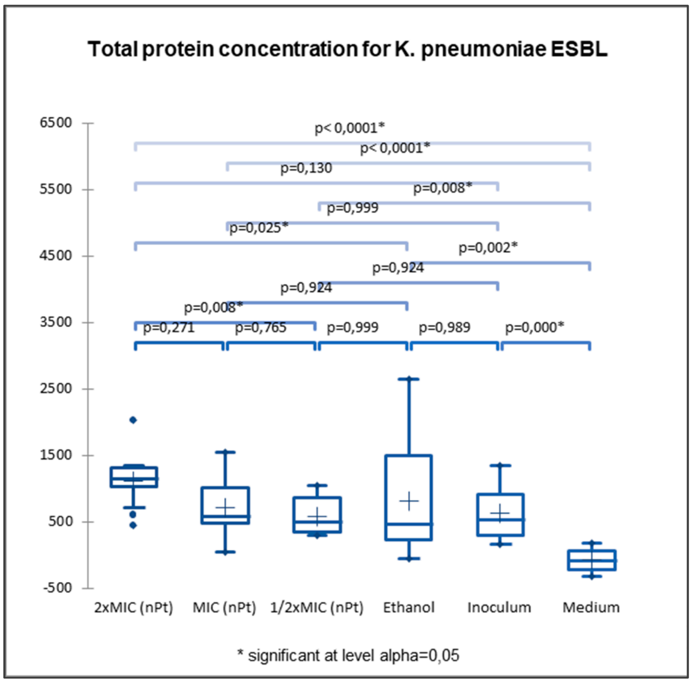

Figure S86. Graphical statistical analysis representation of determined protein concentrations obtained for K. pneumoniae ESBL+ MFBF 10690 exposed to 2 × MIC, MIC, and 1/2 × MIC of nPt as a sum of results of all measurement time points. P values are shown between comparing variables.

Table S84. Statistical significance between comparing variables for the group of results from Figure S86. If p < 0,05 the difference is considered as significant (Yes), if the p > 0,05 the difference is considered as not significant (No).

Significant differences:						
	2xMIC (nPt)	MIC (nPt)	1/2xMIC (nPt)	Ethanol	Inoculum	Medium
2xMIC (nPt)	-	No	**Yes**	**Yes**	No	**Yes**
MIC (nPt)	No	-	No	No	No	**Yes**
1/2xMIC (nPt)	**Yes**	No	-	No	No	**Yes**
Ethanol	**Yes**	No	No	-	No	**Yes**
Inoculum	No	No	No	No	-	**Yes**
Medium	**Yes**	**Yes**	**Yes**	**Yes**	**Yes**	-

2.2.5. Comparison of total protein concentration between tested strains

Statistical test used: Kruskal-Wallis test (nonparametric comparison of n samples)

Statistical significance value (α) = 0,05

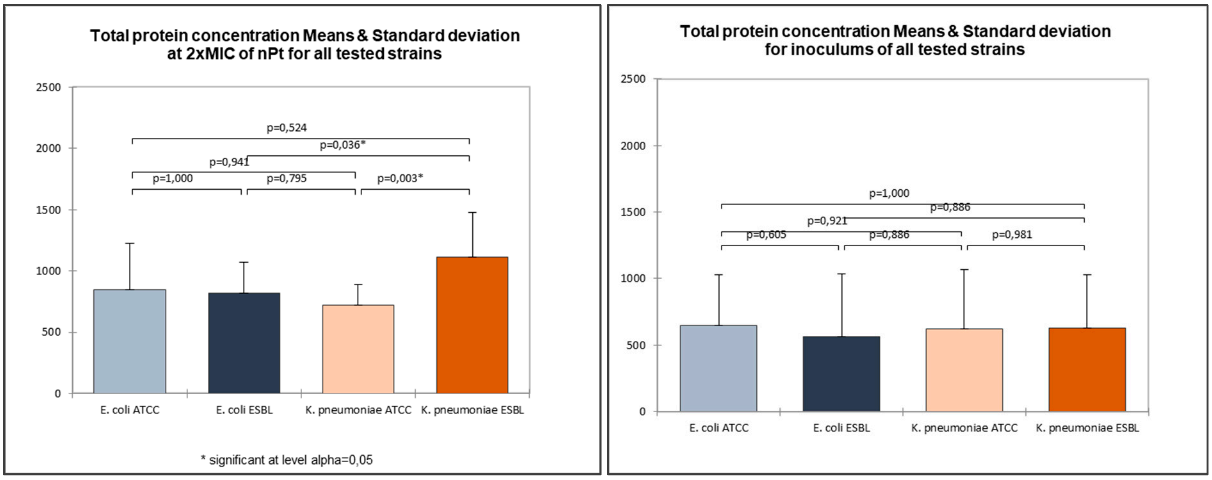

Significant differences:

	E. coli ATCC	E. coli ESBL	K. pneumoniae ATCC	K. pneumoniae ESBL
E. coli ATCC	-	No	No	No
E. coli ESBL	No	-	No	**Yes**
K. pneumoniae ATCC	No	No	-	**Yes**
K. pneumoniae ESBL	No	**Yes**	**Yes**	-

Significant differences:

	E. coli ATCC	E. coli ESBL	K. pneumoniae ATCC	K. pneumoniae ESBL
E. coli ATCC	-	No	No	No
E. coli ESBL	No	-	No	No
K. pneumoniae ATCC	No	No	-	No
K. pneumoniae ESBL	No	No	No	-

Figure S87. Comparison of total protein concentration of all measurements for all tested strains with statistical analysis representation: a) for 2 × MIC of nPt exposure, b) for inoculums (untreated bacteria). P values are shown between comparing variables. If p < 0,05 the difference is considered as significant (Yes), if the p > 0,05 the difference is considered as not significant (No).
